# Distinct evolution of type I glutamine synthetase in *Plasmodium* and its species-specific requirement

**DOI:** 10.1038/s41467-023-39670-4

**Published:** 2023-07-14

**Authors:** Sourav Ghosh, Rajib Kundu, Manjunatha Chandana, Rahul Das, Aditya Anand, Subhashree Beura, Ruchir Chandrakant Bobde, Vishal Jain, Sowmya Ramakant Prabhu, Prativa Kumari Behera, Akshaya Kumar Mohanty, Mahabala Chakrapani, Kapaettu Satyamoorthy, Amol Ratnakar Suryawanshi, Anshuman Dixit, Govindarajan Padmanaban, Viswanathan Arun Nagaraj

**Affiliations:** 1 Infectious Disease Biology, Institute of Life Sciences, Bhubaneswar, 751023 Odisha India; 2 Regional Centre for Biotechnology, Faridabad, 121001 Haryana India; 3 School of Biotechnology, Kalinga Institute of Industrial Technology, Bhubaneswar, 751024 Odisha India; 4 Department of Biotechnology, Manipal School of Life Sciences, Manipal Academy of Higher Education, Manipal, 576104 Karnataka India; 5 Ispat General Hospital, Sector 19, Rourkela, 769005 Odisha India; 6 Department of Medicine, Kasturba Medical College, Mangalore, Manipal Academy of Higher Education, Manipal, 576104 Karnataka India; 7 Department of Cell and Molecular Biology, Manipal School of Life Sciences, Manipal Academy of Higher Education, Manipal, 576104 Karnataka India; 8 Department of Biochemistry, Indian Institute of Science, Bangalore, 560012 Karnataka India

**Keywords:** Enzymes, Parasite biology

## Abstract

Malaria parasite lacks canonical pathways for amino acid biosynthesis and depends primarily on hemoglobin degradation and extracellular resources for amino acids. Interestingly, a putative gene for glutamine synthetase (GS) is retained despite glutamine being an abundant amino acid in human and mosquito hosts. Here we show *Plasmodium* GS has evolved as a unique type I enzyme with distinct structural and regulatory properties to adapt to the asexual niche. Methionine sulfoximine (MSO) and phosphinothricin (PPT) inhibit parasite GS activity. GS is localized to the parasite cytosol and abundantly expressed in all the life cycle stages. Parasite GS displays species-specific requirement in *Plasmodium falciparum* (*Pf*) having asparagine-rich proteome. Targeting *Pf*GS affects asparagine levels and inhibits protein synthesis through eIF2α phosphorylation leading to parasite death. Exposure of artemisinin-resistant *Pf* parasites to MSO and PPT inhibits the emergence of viable parasites upon artemisinin treatment.

## Abstract (web summary)

In a study looking to examine the functional significance of glutamine synthetase (GS), the authors show that in *Plasmodium*, GS has evolved as a distinct type I enzyme with unique biochemical and structural features that complement the parasite niche.

## Introduction

Malaria parasites have lost de novo pathways for amino acid biosynthesis and retained only a few transaminases and enzymes functioning at the junction of nitrogen and carbon metabolism^1–4^. The asexual stage parasites acquire hemoglobin (Hb) from host RBCs and degrade it in the acidic food vacuole (FV) containing various proteases^5^. Hb degradation caters to amino acid requirements and provides space for the parasites to grow. The only amino acid that is absent in human Hb is isoleucine. In vitro cultures of *P. falciparum* (*Pf*) can be maintained continuously by providing isoleucine as the sole amino acid in the culture medium, suggesting that the other amino acids released from Hb degradation are adequate to support asexual growth^6^. Unlike the intracellular development of asexual stages in RBCs, sexual stage development in mosquitoes that spans around 12–18 days is extracellular. Starting from the egress of gametocytes, the zygotes, ookinetes, oocysts, and sporozoites depend primarily on extracellular sources of amino acids that are derived from the ingested blood meal and present in the hemolymph. When the sporozoites invade hepatocytes and undergo exo-erythrocytic schizogony, they may have to depend on host hepatocyte reserves and/or extracellular sources for amino acids^7,8^. Further, sexual and liver stages do not seem to have FV and therefore, lack the machinery for host protein degradation^9^. Malaria parasite undergoes rapid proliferation in the human and mosquito hosts to generate asexual and liver-stage merozoites, and sporozoites. Intriguingly, the parasite accomplishes it by utilizing the amino acids mainly from human and mosquito resources.

Glutamine is required for carbamoyl phosphate and cytidine triphosphate (CTP) synthesis in the de novo pyrimidine pathway of malaria parasite catalyzed by cytosolic carbamoyl phosphate synthetase II and CTP synthetase, respectively^4,10^. Although the parasite lacks de novo purine synthesis and possesses hypoxanthine-guanine phosphoribosyltransferase (HGPRT) to salvage host purines, it has retained guanosine monophosphate synthetase (GMPS) for which, glutamine serves as an amide donor^11,12^. Glutamine is also utilized by glutamine-fructose-6-phosphate transaminase of hexosamine pathway to generate uridine diphosphate N-acetylglucosamine (UDP-GlcNAc) required for glycoprotein, proteoglycan and glycolipid biosynthesis^13^. In vitro metabolic labeling studies of *Pf* asexual and sexual stages carried out with ^13^C-glutamine in the culture medium have suggested a flux of glutamine-derived carbon skeletons into TCA cycle that can possibly lead to the generation of reducing equivalents^14^. Glutamine serves as an amide nitrogen donor for asparagine synthesis as well. Interestingly, glutamine is the most abundant amino acid in human blood with plasma concentrations around 0.5 mM^15^. The asexual stages and gametocytes can also utilize extracellular glutamine as evident from stable isotope labeling studies^14^. In addition, they can acquire glutamine derived from Hb degradation in the FV^6^. Glutamine is also abundant in the mosquito hemolymph^16^ and it represents 40–60% of the total amino acids in human liver tissue^15^. Despite the abundance of glutamine in the host milieu and the ability to access Hb-derived and extracellular glutamine, parasite has retained a putative gene for glutamine synthetase (GS) that is conserved across the *Plasmodium* species infecting humans, primates, rodents and birds^17^. This has prompted us to examine the functional significance of GS in malaria parasites. Here, we show parasite GS is enzymatically active and it belongs to a unique type I enzyme that has evolved in a distinct manner. It is expressed in the cytosol of all the stages of parasite life cycle. By utilizing in vitro cultures of *Pf* for asexual stages and gametocytes, and *P. berghei* (*Pb*) as an in vivo rodent parasite model for the entire life cycle, we show the species-specific differences in GS requirement in the asexual stages of *Plasmodium* and validate them with clinical samples of *Pf* and *P. vivax* (*Pv*) from malaria-infected individuals. Finally, we show the effect of inhibiting GS in artemisinin (ART)-resistant *Pf* strain. All these findings have implications in developing therapeutic strategies for *Pf* - the deadliest human parasite responsible for more than 90% of global malaria infections.

## Results

### *Plasmodium* GS I is enzymatically active

**Fig. 1:**
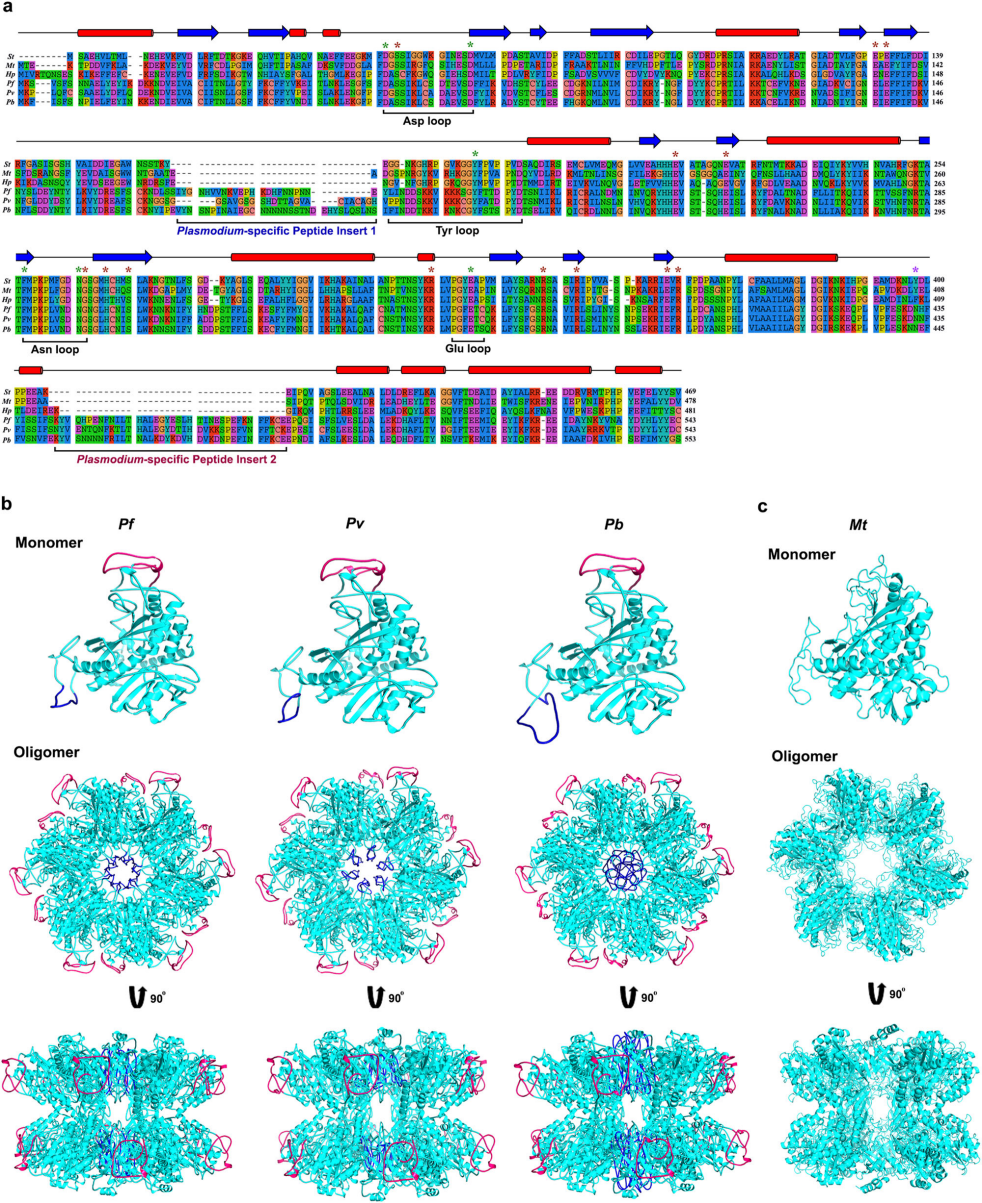
Multiple sequence alignment and homology modeling of *Plasmodia* GS. **a** Multiple sequence alignment of *Plasmodia* GS (*Pf*, *Pv* and *Pb*) with type I GS of *Mt*, *St* and *Hp*. The alignment was carried out with SeaView Version 3.2 (http://pbil.univ-lyon1.fr/software/seaview3). The secondary structures predicted as α-helices and β-strands are represented as red cylinders and blue arrows, respectively. The conserved amino acid residues in the catalytic and flexible loops of ‘bifunnel’ active site - Asp59 and Asp73 present in the aspartate loop, Tyr212 present in the tyrosine loop, Phe287 and Asn296 present in the asparagine loop, and Glu361 present in the glutamate loop are highlighted with green asterisks. The residues that are involved in the substrate and ligand interactions - Ser61, Glu137, Glu139, Glu245, Glu252, Gly297, His301, Ser305, Arg355, Arg373, Arg378, Glu393 and Arg395 are highlighted with brown asterisks. The tyrosine residue that undergoes adenylylation in *Mt* and *St* GS are highlighted with pink asterisk. **b** Monomeric and oligomeric structures of *Pf*, *Pv* and *Pb* GS. *Pv* and *Pb* GS structures were modeled based on the cryoEM structure of *Pf* GS (PDB ID: 6PEW). *Plasmodia* GS-specific peptide inserts are represented in blue (Insert 1) and red (Insert 2). **c** Monomeric and oligomeric structures of *Mt* GS (PDB ID: 2WGS).

GS can be classified into three types: I, II and III based on the sequence and structural conformation (Supplementary Discussion). *Plasmodium* genome has a putative, single-copy gene for GS. Multiple sequence alignment suggested that *Plasmodium* GS is a type I enzyme showing ~50% similarity and ~30% identity with respect to GS I of *Mycobacterium tuberculosis* (*Mt*), *Salmonella typhimurium* (*St*), and *Helicobacter pylori* (*Hp*) (Fig. 1a). The sequence length of *Plasmodia* GS is around 540 amino acids, ~60–70 amino acids more than a typical GS I. This can be attributed to two different peptide inserts representing 174–196 and 443–480 amino acids (numbers represent the positions in *Pf*GS) spanning across the regions that are not directly associated with the substrate-binding and catalytic sites. Further, GS I can be classified into two subtypes based on the regulatory mechanisms - Iα that is regulated through the feedback inhibition by glutamine, adenosine monophosphate (AMP) and other amino acids like serine, alanine and glycine, and Iβ that is additionally inhibited by adenylylation of a tyrosine residue near the active site and not feedback-inhibited by glutamine^18–21^. The characteristic features of GS Iβ are the presence of a specific ~25 amino acid insertion and an adenylylation site with a conserved tyrosine residue (Fig. 1a) that are absent in GS Iα (Supplementary Fig. 1a). Interestingly, the sequence corresponding to 25-amino acid insertion in *Plasmodia* GS is diverged from other organisms and it is flanked by the first peptide insert. Further, *Plasmodia* GS seem to lack a tyrosine residue in the corresponding position of adenylylated tyrosine in *St* and *Mt*GS, flanked by the second peptide insert towards the C-terminus (Fig. 1a). Besides these differences, key amino acid residues of catalytic and flexible loops constituting the ‘bifunnel’ active site existing between the adjacent monomers, and residues interacting with metal and ammonium ions, water molecules, ATP and glutamate are conserved in *Plasmodia* GS^19,20^ (Fig. 1a). Cryo-electron microscopy (cryoEM) of parasite GS has suggested a classical dodecameric GS I structure composed of two hexamers stacked face-to-face^22^. While the first peptide insert that comes in proximity to the pore of the hexamer ring forming dodecamer channel could not be observed in the cryoEM structure, the second peptide insert forms a long loop that folds down in the opposite direction from the active site. Based on the structure of *Pf*GS, we modeled the monomeric and oligomeric structures for *Pb* and *Pv* GS, and the peptide inserts specific for *Plasmodia GS* are represented (Fig. 1b). Our modeled structures of *Plasmodia* GS showed the presence of dodecamer channel with a compact pore arising due to the first peptide insert. For comparison, monomeric and oligomeric structures of *Mt*GS are shown (Fig. 1c) along with the superimposed images of *Pf*, *Pb,* and *Pv* GS monomers with *Mt*GS monomer (Supplementary Fig. 1b). All these evidences suggest that parasite GS belongs to type I enzyme of prokaryotic origin with unique structural features arising out of two peptide inserts that are absent in other GS I. A similar signature is present in GS of *Hepatocystis* (Supplementary Fig. 1c), the closest relative of *Plasmodium* under the phylum Apicomplexa having an analogous life cycle^17^.

**Fig. 2:**
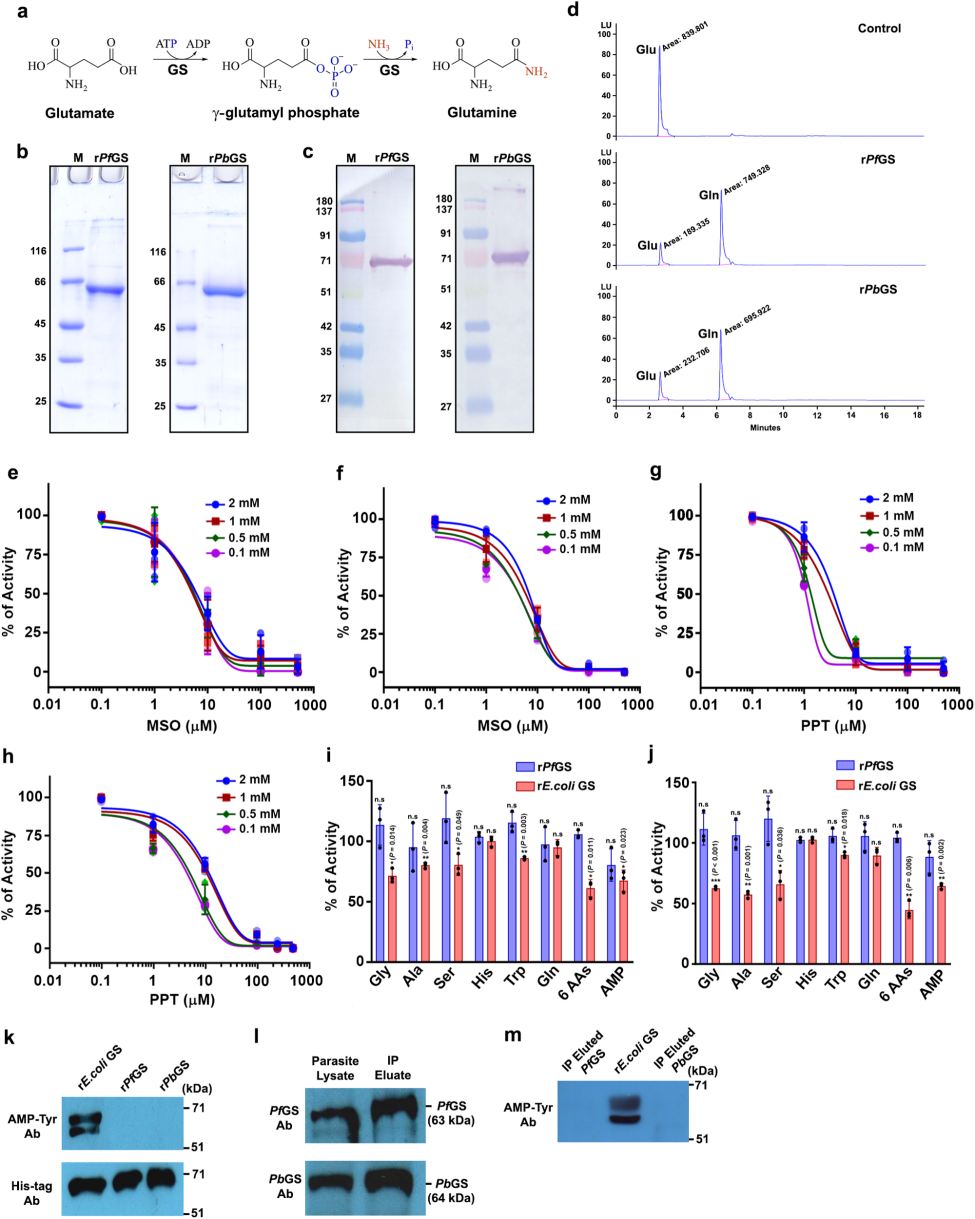
Inhibition and regulation of *Plasmodium* GS. **a** Schematic representation of GS enzymatic reaction. **b,c** Coomassie gel pictures of r*Pf*GS and r*Pb*GS purified using Ni^2+^-NTA resin and their Western blot analysis using anti-his tag antibodies, respectively. Lane M: Protein molecular weight marker (kDa). **d** HPLC chromatogram of r*Pf*GS and r*Pb*GS enzyme assays. **b**–**d** *n* = 3 independent experiments. **e**, **f** Effect of MSO on r*Pf*GS and r*Pb*GS activity, respectively. **g**, **h** Effect of PPT on r*Pb*GS and r*Pb*GS activity, respectively. Percentage of activities (mean ± SD) with respect to the control (without inhibitor) are shown and the assays were independently carried out with 0.1, 0.5, 1.0, and 2.0 mM concentrations of glutamate. *n* = 3 independent protein preparations. **i**, **j** Comparison of the feedback inhibition of r*Pf*GS and r*E.coli* GS in the presence of MgCl_2_ at 1 mM (**i**) and 5 mM (**j**) concentrations of amino acids and AMP. “6AAs” represents the mixture of all the six amino acids. For “6AAs” of 5 mM concentration, tryptophan alone was used at 2.5 mM concentration because of its limited solubility and the rest were used at 5 mM concentration. The percentage of activities with respect to the control (without feedback inhibitor) are shown (mean ± SD; n.s - not significant, *P < 0.05, **P < 0.01, ***P < 0.001, unpaired *t*-test; two-sided). *n* = 3 independent protein preparations. **k** Western blot analysis of r*Pf*GS, r*Pb*GS and r*E.coli* GS adenylylation with anti-AMP-tyrosine antibody. The doublet was observed for r*E.coli* GS could be because of the oligomerization of r*E.coli* GS having ~3 kDa higher molecular weight due to the presence of histidine tag and enterokinase recognition and cleavage sites, with endogenous *E. coli* GS. **l** Western blot analysis of endogenous GS immunoprecipitated from *Pf* and *Pb* parasite lysates. 15 μl of 150 μl parasite lysates and 10 μl of 30 μl immunoprecipitation eluates were used. **m** Western blot analysis of adenylylation in immunoprecipitated *Pf*GS and *Pb*GS. r*E.coli* GS was used as a control. 0.1 μg of eluted protein was used. IP - immunoprecipitation. **k**–**m** *n* = 2 independent experiments. For **e**–**h** individual data points are shown with respective light-shaded colors. Source data are provided as a Source Data file.

Studies on *Plasmodium* biology are widely carried out using *Pf* - a human parasite maintained in vitro, and *Pb* (or *P. yoelii*) - an in vivo rodent parasite model. We sought to understand the functional significance of GS using *Pf* and *Pb*, and checked whether *Pf* and *Pb* GS are enzymatically active. GS catalyzes the synthesis of glutamine by incorporating ammonia into glutamate with concomitant ATP hydrolysis. In the presence of divalent cations such as Mg^2+^ or Mn^2+^, the terminal phosphate from ATP is transferred to glutamate resulting in γ-glutamyl phosphate, which is subsequently attacked by ammonia to form glutamine (Fig. 2a). The purified recombinant (r) *Pf* and *Pb* GS with N-terminal His-tag expressed in *E. coli* showed molecular weight of around ~65 kDa in SDS-PAGE (Fig. 2b, c). HPLC analyzes of purified proteins incubated with glutamate, ammonia, ATP and MgCl_2_ showed the formation of glutamine (Fig. 2d). The specific activity and catalytic efficiency (*K*_*cat*_*/K*_*m*_) were found to be 1.12 ± 0.17 μmol mg^−1^min^−1^ and 1.92 ± 0.29 × 10^4 ^M^−1^S^−1^ for r*Pf*GS, and 1.35 ± 0.47 μmol mg^−1^min^−1^ and 0.96 ± 0.33 × 10^4 ^M^−1^S^−1^ for r*Pb*GS, respectively (Supplementary Fig. 2a, b). Since GS can also exhibit activity in the presence of MnCl_2,_ enzyme assays were performed with 0.5–50 mM MnCl_2_ concentrations. While the optimal activity for parasite GS was obtained with 5 mM MnCl_2_, it was almost 25–30% less when compared with MgCl_2_ suggesting that MgCl_2_ is the preferred metal ion cofactor (Supplementary Fig. 2c). For comparison, we expressed and purified r*E. coli* GS I under identical conditions, and performed the assays (Supplementary Fig. 2d–f). All these data suggest that parasite GS can catalyze the synthesis of glutamine.

### Inhibition and regulation of *Plasmodium* GS activity

L-Methionine sulfoximine (MSO) is a potent irreversible inhibitor of GS. MSO-phosphate generated through the phosphorylation of MSO by GS serves as a transition state analogue that binds non-covalently and stabilizes the flexible loop of GS active site thereby, preventing glutamate entry. In addition, methyl group of MSO-phosphate occupies the ammonium binding site to prevent further reaction^23, 24^. While MSO can inhibit both GS I and II, *K*_*i*_ values reported for GS I are ~10^2^ times lower when compared with GS II^25,26^. Kinetic studies showed that the parasite GS is sensitive to MSO inhibition like other GS I with *K*_*i*_ values of 5.64 ± 0.39 μM and 5.44 ± 1.07 μM for r*Pf* and *Pb*GS, respectively (Fig. 2e, f). Phosphinothricin (PPT; also known as glufosinate), widely used as a broad-spectrum herbicide, is another potent irreversible inhibitor whose mechanism of inhibition is similar to that of MSO^27^. While the sensitivity of bacterial GS to PPT is similar to that of MSO, plant GS is more sensitive to PPT than MSO^26–30^. Parasite GS is equally sensitive to PPT and the respective *K*_*i*_ values obtained for r*Pf* and *Pb*GS were 2.31 ± 1.10 μM and 8.07 ± 3.31 μM (Fig. 2g, h). These data indicate that parasite GS can be inhibited by transition state analogs.

To examine whether parasite GS is regulated by feedback inhibition through end products of glutamine metabolism, we performed enzyme assays with 1 and 5 mM concentrations of glycine, serine, alanine, tryptophan, histidine, glutamine and AMP. *E. coli* GS Iβ is inhibited by alanine, glycine, serine, and tryptophan in the presence of MgCl_2_^31^. *Bs*GS Iα is inhibited almost completely by AMP and glutamine in the presence of MnCl_2_^32^. Similar inhibition by alanine, glycine and serine has been reported for type II rat GS as well^33^. Interestingly, none of the amino acids either tested individually or in a cumulative manner could significantly inhibit r*Pf*GS activity in the presence of MgCl_2_. The inhibition with AMP was also not significant with respect to the control. For comparison, assays were performed with *E. coli* GS. As reported earlier, *E. coli* GS showed 20–30% inhibition for glycine, alanine, serine, and AMP, and 10% inhibition for tryptophan at 1 mM concentration. There was almost 30–40% inhibition for glycine, alanine, serine, and AMP at 5 mM concentration (Fig. 2i, j). Further, glutamine serves as a strong feedback inhibitor for GS 1α, but not for Iβ^32,34^. In agreement, *E. coli* and r*Pf*GS activities were not inhibited by glutamine even at a 5 mM concentration (Fig. 2j). Since the effect of inhibitors can vary with the metal ion cofactors^32^, we performed r*Pf*GS assays in the presence of MnCl_2_ as well. Again, none of the amino acids tested could inhibit the activity, and a moderate inhibition of around 25% was observed only for 5 mM AMP (Supplementary Fig. 3a). Similar results were obtained for r*Pb*GS with respect to amino acids and there was a moderate inhibition by AMP in the presence of MgCl_2_ and MnCl_2_ (Supplementary Fig. 3b, c). Another salient feature of type Iβ GS is its regulation by adenylylation^35,36^. Interestingly, Western analysis using anti-AMP-tyrosine antibody showed that both r*Pf* and r*Pb* GS do not undergo adenylylation, although *E. coli* GS exhibits adenylylation (Fig. 2k). This was also confirmed by performing immunoprecipitation for endogenous GS from *Pf* and *Pb* parasite lysates, wherein, adenylylation could not be detected (Fig. 2l, m). These results suggest that parasite GS is unique and it lacks the characteristic regulatory mechanisms of GS 1α or 1β.

### Functional significance of *Plasmodium*-specific peptide inserts

**Fig. 3:**
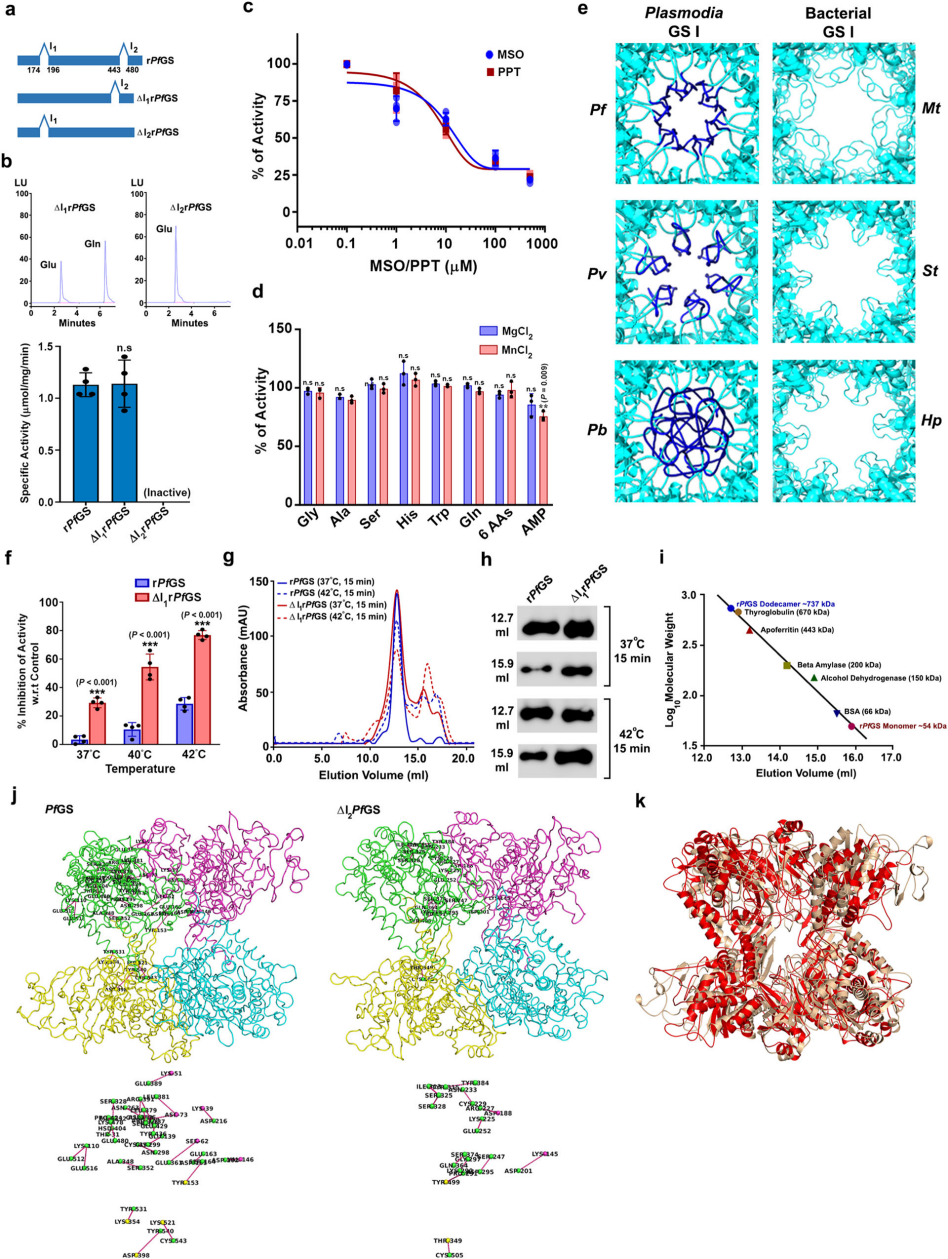
Characterization of r*Pf*GS lacking the first and second peptide inserts. **a** Schematic representations showing deletions of first and second peptide inserts. **b** HPLC chromatograms of ΔI_1_r*Pf*GS and ΔI_2_r*Pf*GS enzyme assays. Specific activities (mean ± SD shown below represent four different protein preparations. Lack of enzyme activity in ΔI_2_r*Pf*GS was also verified with MnCl_2_ and for a prolonged incubation of 6 h. **c** Effect of MSO and PPT on ΔI_1_r*Pf*GS activity. Individual data points are shown with the respective light shaded colors. **d** Feedback inhibition of ΔI_1_r*Pf*GS in the presence of MgCl_2_ and MnCl_2_ at 5 mM concentrations of amino acids and AMP. For **c** and **d** percentage of activities (mean ± SD) with respect to control (without inhibitor/feedback inhibitor) are shown. *n* = 3 different protein preparations. **e** Comparison of *Plasmodia* and bacterial GS I dodecamer channels. *Mt*, *St,* and *Hp* structures were retrieved from PDB. **f** Comparison of r*Pf*GS and ΔI_1_r*Pf*GS thermal stabilities. Percentage of inhibition of the activities (mean ± SD) with respect to unexposed controls are shown. *n* = 3 different protein preparations. Recombinant proteins were exposed to the respective temperatures for one hour before performing assays at 37 °C. **g** Chromatograms showing the dissociation of oligomers in r*Pf*GS and ΔI_1_r*Pf*GS. Protein preparations were exposed to 37 °C or 42 °C for 15 min and subjected immediately to size-exclusion chromatography. **h**, Western analysis of r*Pf*GS (~65 kDa) and ΔI_1_r*Pf*GS (~63 kDa) in 12.7 and 15.9 ml elution volume fractions. **i** Estimation of r*Pf*GS molecular weights eluted at 12.7 and 15.9 ml based on the elution of standard proteins. For **g**–**i** *n* = 2 independent protein preparations. **j** Unique hydrogen bond interactions of *Pf*GS and ΔI_2_r*Pf*GS are shown for subunit A. Four subunits are represented in green (subunit A), pink (subunit B), yellow (subunit C) and cyan (subunit D). Interactions without the subunit background are shown below. **k** Superimposition of MD simulation structures of two adjacent subunits from upper and lower hexamers of *Pf*GS (grey) and ΔI_2_r*Pf*GS (red). (n.s - not significant, **P < 0.01, ***P < 0.001, unpaired t-test; two-sided). Source data are provided as a Source Data file.

To understand the functional significance of *Plasmodium* GS-specific peptide inserts, we performed independent deletions for first (ΔI_1_r*Pf*GS) and second peptide inserts (ΔI_2_r*Pf*GS) of r*Pf*GS (Fig. 3a; Supplementary Fig. 3d, e), and examined their activities. ΔI_1_r*Pf*GS behaved like r*Pf*GS in terms of specific activity (Fig. 3b), inhibition by MSO and PPT (Fig. 3c), and lack of feedback inhibition by amino acids (Fig. 3d). Since the first peptide insert occupying hexamer pore of the dodecameric channel renders it more compact than GS I of other organisms (Fig. 3e), we were interested in examining the stability of ΔI_1_r*Pf*GS. While the activity of ΔI_1_r*Pf*GS was comparable with r*Pf*GS when exposed to higher concentrations of urea or NaCl (Supplementary Fig. 3f, g), ΔI_1_r*Pf*GS was thermally less stable. Exposure of ΔI_1_r*Pf*GS to increased temperatures in the febrile range of 37 °C to 42 °C for one hour could lead to visible precipitation and almost 80% reduction in its activity in comparison with ~30% reduction observed for r*Pf*GS (Fig. 3f). Size-exclusion chromatography examining the oligomeric status showed the dissociation of oligomers when ΔI_1_r*Pf*GS was exposed to 37 °C or 42 °C for 15 min (Fig. 3g–i). Interestingly, deletion of the second peptide insert (ΔI_2_r*Pf*GS) led to loss of enzyme activity under various conditions that were tested (Fig. 3b). For additional insights, we performed all-atom molecular dynamics (MD) simulations for the two adjacent subunits constituting the active site from upper and lower hexamers of *Pf*GS and ΔI_2_*Pf*GS. A comparison of intra-chain and inter-chain hydrogen bonds of one subunit suggested 25 and 12 interactions that were unique for *Pf*GS and ΔI_2_*Pf*GS, respectively (Fig. 3j). This also included some of the residues that are directly associated with the enzyme activity (Supplementary Data 1). Interestingly, of the 25 unique interactions in *Pf*GS, only two of them were from the second peptide insert. The analyzes of MD simulations showed changes in the overall protein conformation (Fig. 3k) with deviations in C_α_ backbone, residue flexibility, hydrogen bond formation, and solvent accessible surface area (SASA) (Supplementary Fig. 3h–k). As observed for ΔI_2_r*Pf*GS, r*Pf*GS lacking both the inserts (ΔI_1_I_2_r*Pf*GS) was also found to be inactive (Supplementary Fig. 3l). These results suggest that the evolution of the second peptide insert has occurred with compensatory structural changes in *Pf*GS that affect *Pf*GS activity when the second peptide insert was deleted.

### *Plasmodium* GS is cytosolic and expressed in the entire life cycle

**Fig. 4:**
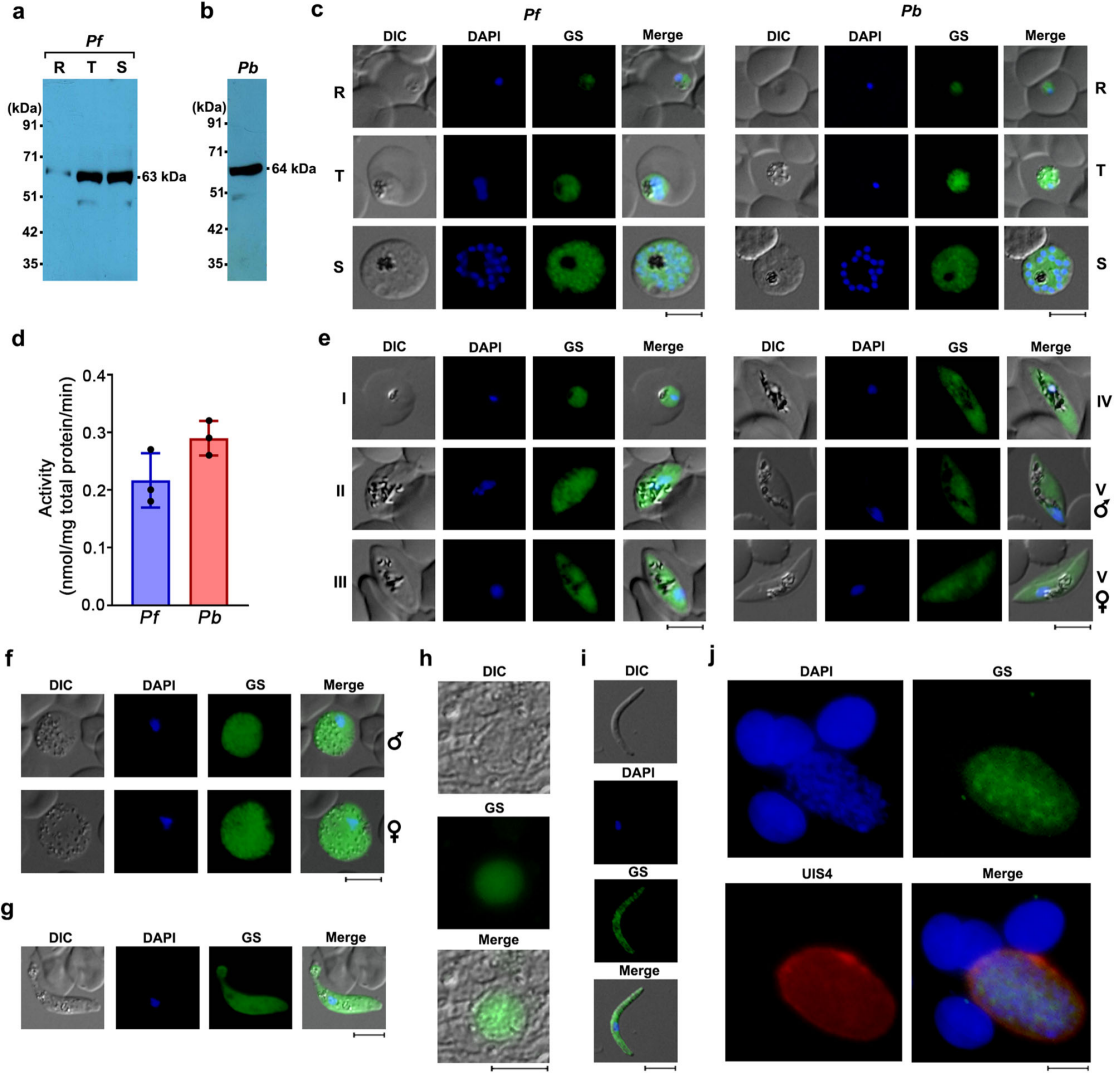
Expression of GS in the life cycle of *Plasmodia*. **a** Western analysis of GS expression in the lysates of *Pf* rings, trophozoites and schizonts. Equal number of rings (R), trophozoites (T) and schizonts (S) were used from 10 ml of tightly synchronized cultures. **b** Western analysis of GS expression in *Pb* parasite lysate. 50 μg of total protein was used. **c** Immunofluorescence analysis of GS expression in *Pf* and *Pb* rings (R), trophozoites (T) and schizonts (S). Scale bar = 5 μM. For **a**–**c**, *n* = 3 independent experiments. **d** GS activity in the parasite lysates of *Pf* and *Pb*. The activity (mean ± SD) was determined with respect to the total protein. *n* = 3 independent preparations. **e** Immunofluorescence analysis of GS expression in *Pf* gametocytes (Stage I-V). Scale bar = 5 μM. **f** Immunofluorescence analysis of GS expression in *Pb* gametocytes. Scale bar = 5 μM. **g**–**i** Immunofluorescence analysis of GS expression in *Pb* ookinete, oocyst and sporozoite, respectively. Scale bar for ookinete and sporozoite = 5 μM. Scale bar for oocyst = 20 μM. **j**, Immunofluorescence analysis of GS expression in *Pb* exo-erythrocytic stage. UIS4 antibody was used to identify the exo-erythrocytic stage. Scale bar = 20 μM. All the images were captured using 60x/100x objective. Oocyst image was captured using 20x objective. For **e**–**j** *n* = 2 independent experiments. Source data are provided as a Source Data file.

We examined the expression and localization of native GS, and its activity in the parasite. Western analyzes carried out for *Pf* lysates having equal number of synchronized rings, trophozoites and schizonts using *Pf*GS antibodies showed GS expression in all the asexual stages (Fig. 4a). Similarly, GS expression could be detected in the lysates of *Pb* asexual stages (Fig. 4b). Immunofluorescence analyzes showed an abundant cytosolic localization wherein, the fluorescence signal was observed all over the parasite in rings, trophozoites and schizonts (Fig. 4c). This was in agreement with the absence of any predictable signal sequence in the parasite GS^17^. Enzyme assays to assess the functionality of native GS in asexual stages showed a GS activity of around 0.21 and 0.29 nmol mg^−1^ total protein min^−1^ for *Pf* and *Pb* lysates, respectively (Fig. 4d). Further, GS expression was detected in *Pf* and *Pb* gametocytes (Fig. 4e, f). Given the safety constraints associated with performing sexual stage development of *Pf* in mosquitoes, we utilized *Pb* to examine GS expression in these stages. Immunofluorescence analyzes for the sexual stage development of *Pb* in *Anopheles stephensi* mosquitoes showed GS expression in ookinete, oocyst and sporozoite stages (Fig. 4g–i). GS expression could also be detected for in vitro exo-erythrocytic stages of *Pb* when the sporozoites from infected mosquitoes were allowed to infect immortalized human hepatocyte cell line HC-04 (Fig. 4j). All these evidences suggest that native parasite GS is cytosolic and active, and abundantly expressed in all the stages of the parasite life cycle.

### GS is required for the optimal development of *Pf* asexual and gametocyte stages

**Fig. 5:**
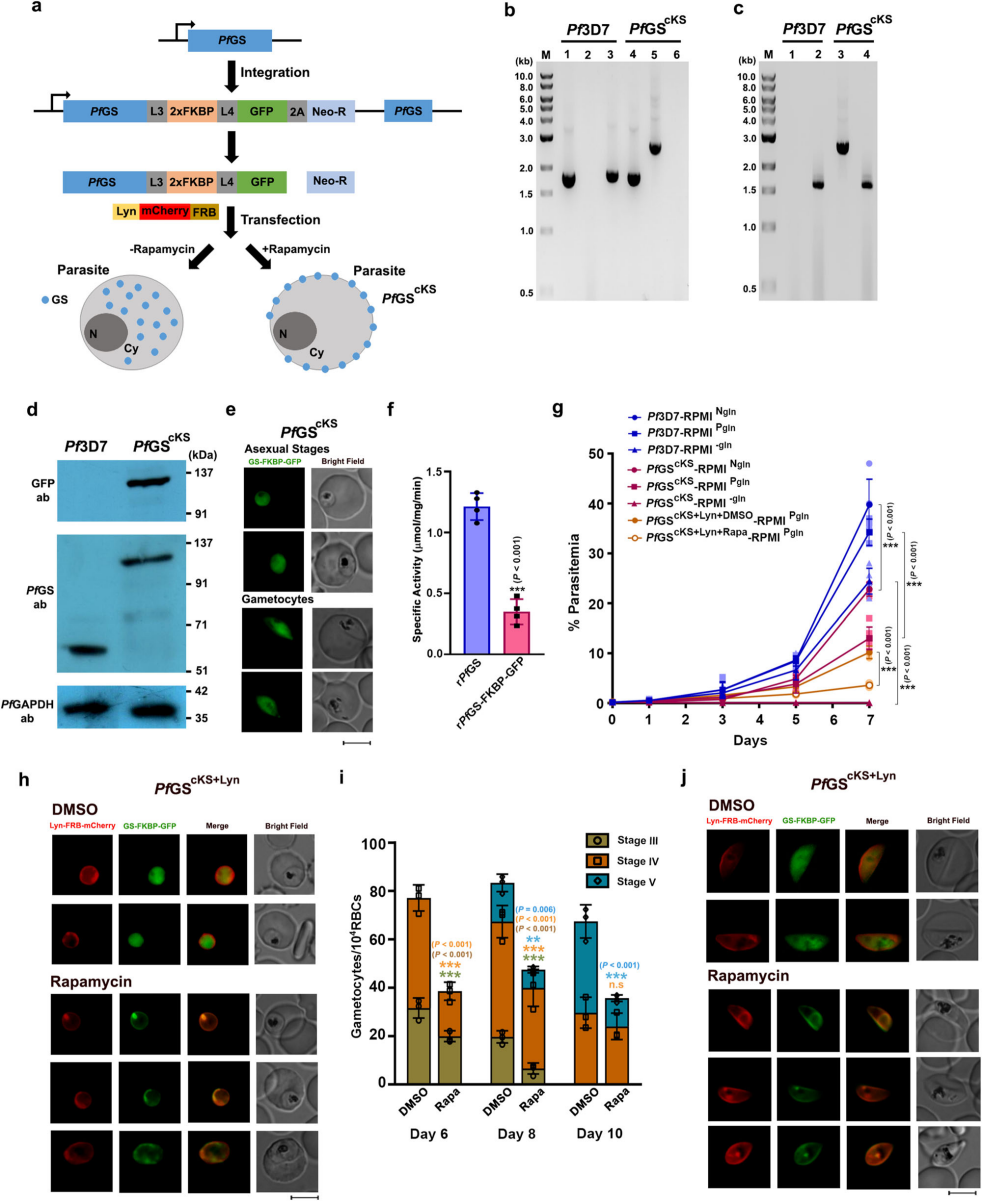
Conditional knock sideways of GS in *Pf*. **a** Schematic representation of conditional knock sideways approach to mislocalize *Pf*GS. **b** Genomic DNA PCR confirmation for *Pf*GS^cKS^ parasites. Lane 1 and 4: 1.76 kb product amplified with GS-specific forward and reverse primers. Lane 2 and 5: 2.64 kb product amplified with GS-specific forward and GFP-specific reverse primers to confirm the in-frame fusion. Lane 3 and 6: 1.82 kb product amplified with GS-specific forward and 3’ UTR-specific reverse primer to confirm the integration. Lane M: 1 kb ladder. **c** RT-PCR confirmation for *Pf*GS^cKS^ parasites. Lane 1 and 3: 2.51 kb product amplified with GS-specific forward and GFP-specific reverse primers. Lane 2 and 4: 1.63 kb product amplified with GS-specific forward and reverse primers. Lane M: 1 kb ladder. **d** Western blot confirmation for GS-FKBP-GFP fusion in *Pf*GS^cKS^ parasites. Upper panel: Confirmation of 120 kDa fusion protein in *Pf*GS^cKS^ parasites with GFP antibody. Middle panel: Confirmation with *Pf*GS antibody. Lower panel: Parasite GAPDH as a loading control. **e** Live imaging of GS-FKBP-GFP localization in *Pf*GS^cKS^ parasites. Images were captured using 100x objective. Scale bar = 5 μM. For **b**–**e** *n* = 3 independent experiments. **f** Specific activity of r*Pf*GS-FKBP-GFP fusion protein in comparison with r*Pf*GS. (mean ± SD; **P < 0.001, unpaired t-test; two-sided). *n* = 4 independent assays performed with two different protein preparations. **g** Asexual stage growth analysis of *Pf*3D7 and *Pf*GS^cKS^ parasites in RPMI^Ngln^, RPMI^Pgln^ and RPMI^-gln^ medium, and *Pf*GS^cKS+Lyn^ parasites in RPMI^Pgln^ medium. (mean ± SD; ***P ≤ 0.001, Two-way ANOVA). Rapa - rapamycin. *n* = 4 independent experiments. **h** Live fluorescence analysis of GS mislocalization in rapamycin-treated *Pf*GS^cKS+Lyn^ asexual stages. Images were captured using 100x objective. Scale bar = 5 μM. **i** Analysis of gametocyte maturation in cKS-induced *Pf*GS^cKS+Lyn^ parasites in RPMI^Pgln^ medium. (mean ± SD; n.s - not significant, **P < 0.01, ***P < 0.001, Two-way ANOVA) Rapa - rapamycin. *n* = 3 independent experiments. **j** Live fluorescence analysis of GS mislocalization in rapamycin-treated *Pf*GS^cKS+Lyn^ gametocytes, respectively. Images were captured using 100x objective. Scale bar = 5 μM. *n* = 3 independent experiments. Source data are provided as a Source Data file.

Our next interest was to understand the significance of GS in asexual and gametocyte stages of *Pf*. Repeated attempts to generate GS knockout (KO) in *Pf* using conventional double crossover recombination, selection-linked integration and CRISPR-Cas9 approaches turned out to be unsuccessful. Therefore, we generated a conditional, mislocalizing, knock sideways (cKS) 3D7 strain for GS (*Pf*GS^cKS^) through selection-linked integration approach^37^ wherein, GS was fused in-frame with FKBP and GFP through linkers, followed by a neomycin selectable marker separated by skip peptide (Fig. 5a). The integration was confirmed by PCR analyzes for the genomic DNA and RNA isolated from *Pf*GS^cKS^ parasites (Fig. 5b, c). Western analysis carried out with GFP and GS antibodies confirmed the expression of 120 kDa fusion protein in *Pf*GS^cKS^ parasites with levels comparable to parental 3D7 strain (Fig. 5d). Since the expression of GS fused with FKBP-GFP in *Pf*GS^cKS^ parasites is driven by native promoter, it helped us to verify the GS localization results obtained with polyclonal antibodies. Live imaging showed GFP fluorescence all over the parasite reconfirming the cytosolic localization of GS (Fig. 5e). To check the effect of FKBP-GFP fusion on *Pf*GS activity, we purified r*Pf*GS-FKBP-GFP and performed enzyme assays. While r*Pf*GS-FKBP-GFP was capable of forming glutamine, its specific activity was almost 70% less in comparison to r*Pf*GS (Fig. 5f; Supplementary Fig. 4a, b). In agreement, *Pf*GS^cKS^ parasites displayed ~60% reduction of growth in RPMI-1640 medium containing physiological levels (0.5 mM) of glutamine (RPMI^Pgln^) when compared with *Pf*3D7 parasites, on day 7 representing three asexual cycles. Similar results were also obtained with normal RPMI-1640 medium containing 2 mM glutamine (RPMI^Ngln^) (Fig. 5g). Moreover, *Pf*3D7 cultures could be maintained continuously for several days in a glutamine-free RPMI-1640 medium (RPMI^-gln^) and we examined it for over 90 days (Supplementary Fig. 4c). Flow cytometry analysis performed at 48 h intervals for five successive cycles showed only a 15–20% decrease in the parasite multiplication rate for each cycle (Supplementary Fig. 4d), suggesting that glutamine derived from endogenous GS activity and Hb degradation are adequate to support the parasite growth. Interestingly, unlike *Pf*3D7, *Pf*GS^cKS^ parasites failed to grow in RPMI^-gln^ and no viable parasites could be detected after 4 days of glutamine removal (Fig. 5g; Supplementary Fig. 4e). All these results suggest that intervention of endogenous GS activity affects the growth of *Pf* asexual stages, highlighting its significance in *Pf*.

To perform GS mislocalization, *Pf*GS^cKS^ strain was transfected with a plasmid that expressed plasma membrane-targeting Lyn peptide fused with FRB and mcherry. After selecting the transfected *Pf*GS^cKS^ parasites (*Pf*GS^cKS+Lyn^) with blasticidin, GS was knocked sideways from cytosol to the plasma membrane by the addition of rapamycin (Fig. 5a). This was evident from the change in the localization of GFP signal (Fig. 5h; Supplementary Fig. 4f). Since the active sites of GS are formed by adjacent monomers, membrane anchoring driven by rapamycin-induced dimerization of GS-FKBP-GFP with Lyn-FRB-mCherry would disrupt the oligomerization of GS and abrogate GS activity. The rapamycin-induced mislocalization of GS from its site of action caused further reduction in *Pf*GS^cKS+Lyn^ parasite growth in RPMI^Pgln^, leading to almost 90% decrease in comparison with *Pf*3D7 parasites (Fig. 5g). Further, rapamycin alone did not have any effect on *Pf*3D7 and *Pf*GS^cKS^ asexual stage growth (Supplementary Fig. 4g). Western analysis and enzyme assays carried out for the cytosol and membrane fraction of rapamycin-induced *Pf*GS^cKS+Lyn^ parasites confirmed the mislocalization of GS and the loss in GS activity due to mislocalization (Supplementary Fig. 4h, i). Since GS expression was also detectable in the sexual stages, we examined its significance in the sexual stages of *Pf*GS^cKS+Lyn^ maintained in RPMI^Pgln^. Treatment of cultures with rapamycin on day three post-induction of gametocytes led to almost 70% reduction in the formation of mature, stage V gametocytes in comparison with untreated control (Fig. 5i, j). Again, rapamycin alone had no effect on gametocyte formation (Supplementary Fig. 4j). All these findings demonstrate the requirement of endogenous GS for the development of *Pf* asexual stages and gametocytes, signifying that Hb-derived and extracellular glutamine are inadequate for the optimal growth of *Pf*. We generated another transgenic line (*Pf*GS^HA-DD^) to perform conditional knockdown wherein, GS was fused with HA-tagged destabilization domain (HA-DD)^38^. Like *Pf*GS^cKS^ parasites, *Pf*GS^HA-DD^ parasites displayed decreased growth in RPMI^Pgln^ and could not survive in RPMI^-gln^. However, *Pf*GS^HA-DD^ parasites did not support GS knockdown, probably due to the abundant GS expression, and its complex oligomeric nature limiting the exposure of DD to Shield-1 and proteasomal degradation (Supplementary Fig. 5a–e, Supplementary Discussion).

### GS is dispensable for the entire life cycle of *Pb*

**Fig. 6:**
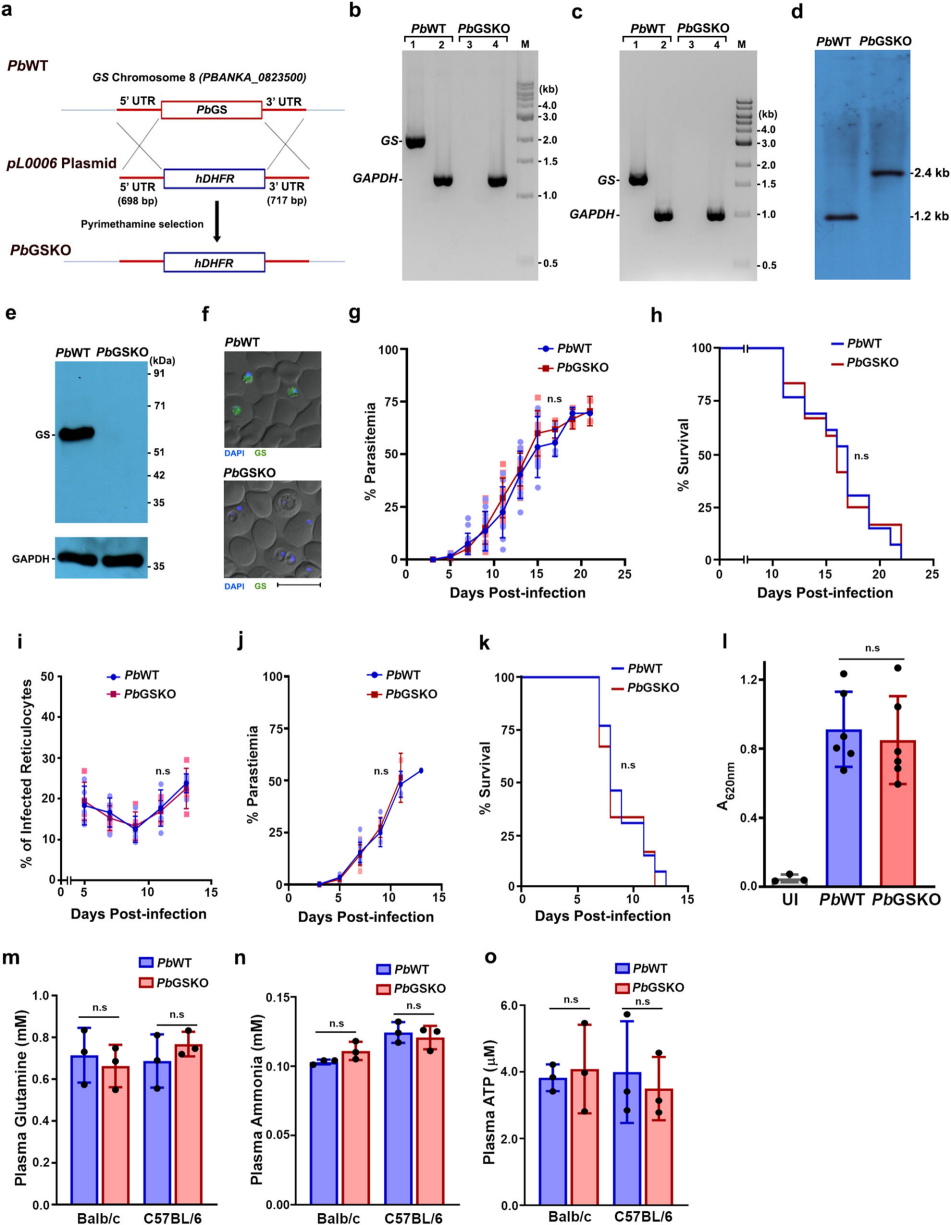
Characterization of *Pb*GSKO in the asexual stages. **a** Double crossover recombination strategy utilized for the generation of *Pb*GSKO parasites. **b** Genomic DNA PCR confirmation for GS deletion in *Pb*. Lane 1 and 3: GS amplification (2.04 kb). Lane 2 and 4: *Pb*GAPDH amplification (1.25 kb). Lane M: 1 kb ladder. **c** RT-PCR confirmation for GS deletion. Lane 1 and 3: GS amplification (1.66 kb). Lane 2 and 4: GAPDH amplification (1.01 kb). Lane M: 1 kb ladder. **d** Southern blot analysis to confirm GS deletion. **e** Western blot confirmation of GS deletion. 50 μg total protein was loaded. GAPDH was used as control. **f** Immunofluorescence confirmation for GS deletion. Scale bar = 10 μM. For **b**–**f** *n* = at least 2 independent experiments. **g** Growth analysis of *Pb*WT (*n* = 13) and *Pb*GSKO (*n* = 13) in Balb/c mice. 10^5^ parasites were used to initiate infections. (mean ± SD; n.s - not significant, Two-way ANOVA). **h** Mortality curves of mice infected with *Pb*WT (*n* = 13) and *Pb*GSKO (*n* = 12) parasites in Balb/c mice. (n.s - not significant, log-rank (Mantel-Cox) test). **i** Percentage of infected reticulocytes in parasitized red cells of *Pb*WT (*n* = 5) and *Pb*GSKO-infected mice (*n* = 5). (mean ± SD; n.s - not significant, Two-way ANOVA). **j** Growth analysis of *Pb*WT (*n* = 13) and *Pb*GSKO (*n* = 12) in C57BL/6 mice. 10^5^ parasites were used to initiate infections. (mean ± SD; n.s - not significant, Two-way ANOVA). **k** Mortality curves of mice infected with *Pb*WT (*n* = 13) and *Pb*GSKO (*n* = 12) parasites in C57BL/6 mice. (n.s - not significant, log-rank (Mantel-Cox) test). **l** Quantification of Evans blue extravasation in the brain samples of mice infected with *Pb*WT and *Pb*GSKO parasites (*n* = 3). (mean ± SD; n.s - not significant, unpaired t-test; two-sided). UI - uninfected mouse. **m**–**o** Estimation of plasma glutamine (**m**), ammonia (**n**) and ATP (**o**) in *Pb*WT- and *Pb*GSKO-infected Balb/c and C57BL/6 mice (*n* = 3). (mean ± SD; n.s - not significant, unpaired t-test; two-sided). Source data are provided as a Source Data file.

We then examined the essentiality of GS in *Pb*. In contrast to *Pf*, GS could be deleted in *Pb* through double crossover recombination (Fig. 6a) as verified by PCR with genomic DNA and total RNA isolated from *Pb*GSKO parasites (Fig. 6b, c), and by Southern analysis (Fig. 6d). This was also confirmed at the protein level by Western and immunofluorescence analyzes (Fig. 6e, f). Interestingly, the growth of GSKO parasites in Balb/c mice (mouse model of anemia in rodent parasite infections) was similar to that of wildtype (WT) parasites and there was no significant difference in the mortality of GSKO-infected mice (Fig. 6g, h). The mortality due to anemia started around day 12 and almost 80% of the mice did not survive beyond day 20. There was also no significant difference in RBC versus reticulocyte preference of *Pb*GSKO parasites (Fig. 6i). Since host glutamine metabolism has been implicated in cerebral malaria (CM)^39^, we examined whether GS deletion in *Pb* has any impact on cerebral pathogenesis in CM-susceptible C57BL/6 mice. Again, no significant differences could be observed in the parasite growth or CM mortality (Fig. 6j, k). Almost 70% of WT- or GSKO-infected mice succumbed to CM within day 10 when the blood parasitemia was around 20% and the rest died of anemia. The infected mice showed typical symptoms of CM such as coma, ataxia, paralysis etc., and the loss of blood brain-barrier integrity could be observed in Evans blue extravasation assays (Fig. 6l). Plasma levels of glutamine were comparable between WT- and GSKO-infected mice suggesting that GS deletion did not affect the host extracellular glutamine (Fig. 6m). For each molecule of glutamine produced by GS, one molecule of ammonia and ATP are utilized. Considering the neurotoxicity of ammonia and the ability of ATP to act as a danger signal in vertebrate host^40, 41^, we examined the plasma levels of ammonia and ATP in GSKO-infected mice and the levels were comparable with WT-infected mice (Fig. 6n, o). These results suggest that GS is dispensable for the asexual stage development of *Pb*, and GS deletion does not alter the outcome of anemia or CM pathogenesis in mice.

**Fig. 7:**
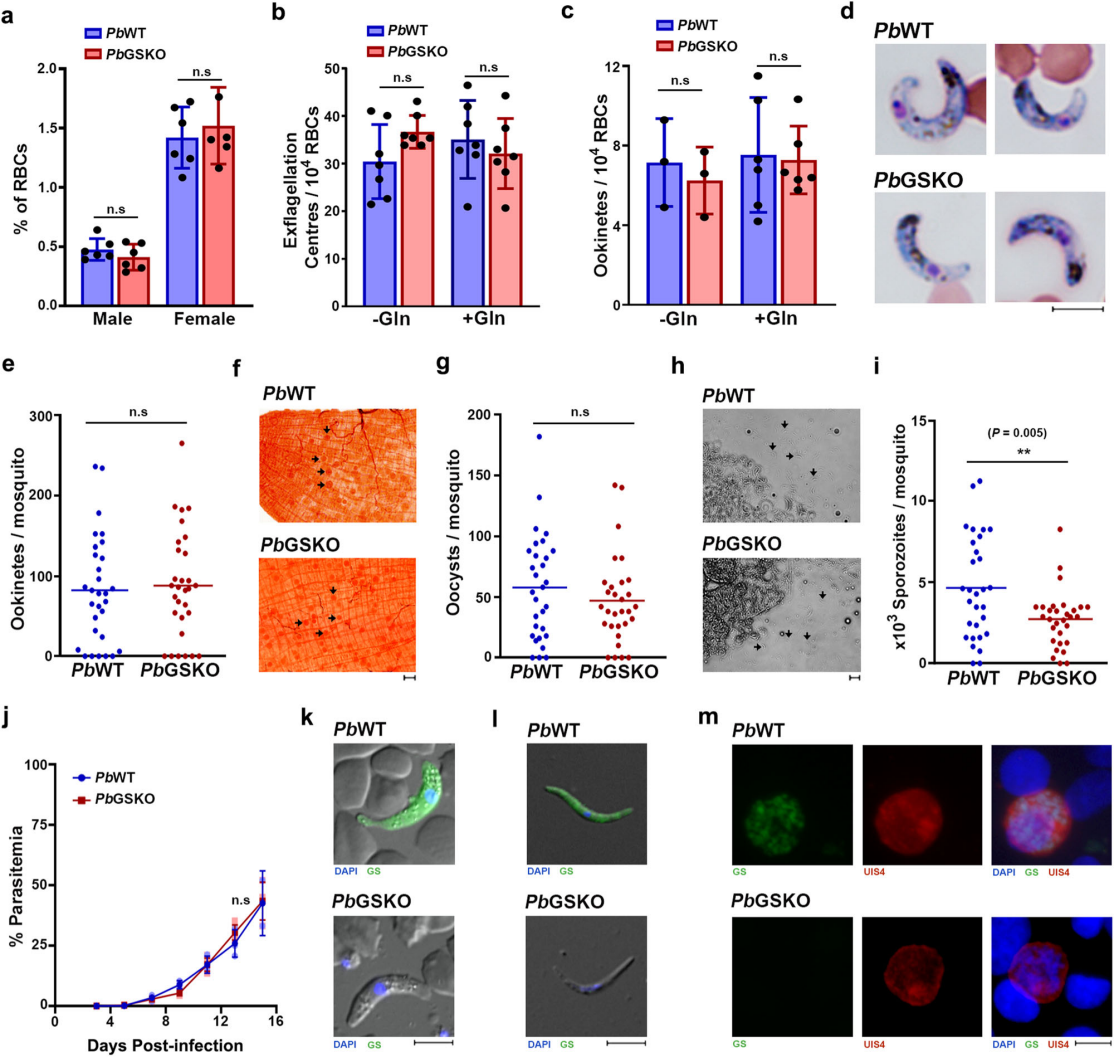
Sexual and exo-erythrocytic stage development of *Pb*GSKO parasites. **a** Number of male and female gametocytes observed in Giemsa-stained smears of Balb/c mice (*n* = 6) prepared on day 8 post-infection. **b** Number of exflagellation centers observed in glutamine-free (Gln^-^) and glutamine-containing (Gln^+^) exflagellation medium for the blood collected on day 8 post-infection. *n* = 7 different Balb/c mice. **c** In vitro ookinete formation in glutamine-free (Gln^-^) and glutamine-containing (Gln^+^) medium. *n* = 3 different Balb/c mice. For **a**–**c** (mean ± SD; n.s - not significant, unpaired t-test; two-sided). **d** Giemsa-stained images for in vivo ookinetes observed in smears prepared from the blood bolus collected at 21 h post-fed mosquito guts. Images were captured using 100x objective. Scale bar = 5 μM. **e** In vivo ookinete formation in the mosquito guts dissected at 21 h post-feeding. **f** Mercurochrome staining for the day 10 post-fed mosquito guts. Black arrows indicate oocysts. Images were captured using 20x objective. Scale bar = 20 μM. **g** In vivo oocyst formation in the mosquito guts dissected on day 10 post-feeding. **h** Bright field images of salivary glands from day 16 post-fed mosquitoes. Black arrows indicate sporozoites. Scale bar = 20 μM. **i** In vivo sporozoite formation in the mosquito salivary glands dissected on day 17 post-feeding. For **d**–**i** *n* = 30 mosquitoes from three independent batches. (n.s - not significant, **P < 0.01, unpaired t-test; two-sided) **j** Growth curve analysis performed for Balb/c mice infected with sporozoites assessing the ability of *Pb*GSKO sporozoites to undergo exo-erythrocytic stage development. Appearance of blood stage parasites was monitored by Giemsa smears prepared form peripheral blood. *n* = 3. (mean ± SD; n.s - not significant, Two-way ANOVA). **k**–**m** Immunofluorescence analysis of *Pb*GSKO ookinete (**k**), sporozoite (**l**) and exo-erythrocytic stage (**m**) with *Pb*GS antibodies. *n* = 2 independent experiments. Scale bar for ookinete and sporozoite = 5 μM. UIS4 antibody was used to identify the exo-erythrocytic stages. Scale bar for exo-erythrocytic stage = 20 μM. Images were captured using 60x/100x objective. Source data are provided as a Source Data file.

Our next interest was to understand its essentiality in the sexual and liver stage development of *Pb*. The number of male and female gametocytes, exflagellation centers observed for male gametocytes and ookinetes formed in vitro were comparable between WT and GSKO parasites (Fig. 7a–c). To assess the contribution of extracellular glutamine, we examined the male gametocyte exflagellation and ookinete formation of GSKO parasites in vitro in medium lacking glutamine. Interestingly, GSKO parasites could undergo exflagellation and give rise to ookinetes in the absence of extracellular glutamine (Fig. 7b, c) suggesting that glutamine reservoir generated in the gametocytes through Hb digestion and/or extracellular uptake was adequate to support until ookinete formation that spans for a duration of 21 h. These findings could not be extended for oocyst and sporozoite development due to the lack of robust in vitro culture techniques. In vivo assessment of the sexual stage development in mosquitoes showed no significant differences in the number of ookinetes (Fig. 7d, e) and oocysts present in the gut (Fig. 7f, g). However, there was a significant 40% reduction in the sporozoites of salivary glands from GSKO-infected mosquitoes in comparison with WT-infected mosquitoes (Fig. 7h, i). These findings suggest that glutamine derived from mosquito hosts could support a major portion of *Pb*GSKO sexual stage development albeit a significant decrease in sporozoites was observed towards the end. Further, we examined the ability of GSKO sporozoites to undergo exo-erythrocytic stage development in the liver. Intravenous injection of 2 × 10^4^ sporozoites into naïve mice showed that GSKO sporozoites could complete exo-erythrocytic stage development as evidenced from the appearance of blood-stage infections. The pre-patent period was comparable between WT and GSKO-infected mice and blood stage parasites were detectable on day 5. There was no significant difference in the growth of sporozoite-derived GSKO asexual stages (Fig. 7j). These results were also confirmed by direct blood-feeding experiments. The absence of GS was verified by performing immunofluorescence for GSKO ookinetes and sporozoites isolated from the mosquitoes (Fig. 7k, l), and exo-erythrocytic stages grown in vitro using HC-04 cell line, using *Pb*GS antibodies (Fig. 7m). All these results suggest the dispensable nature of GS in the entire life cycle of *Pb*.

### MSO and PPT inhibit the growth of *Pf*, but not of *Pb*

**Fig. 8:**
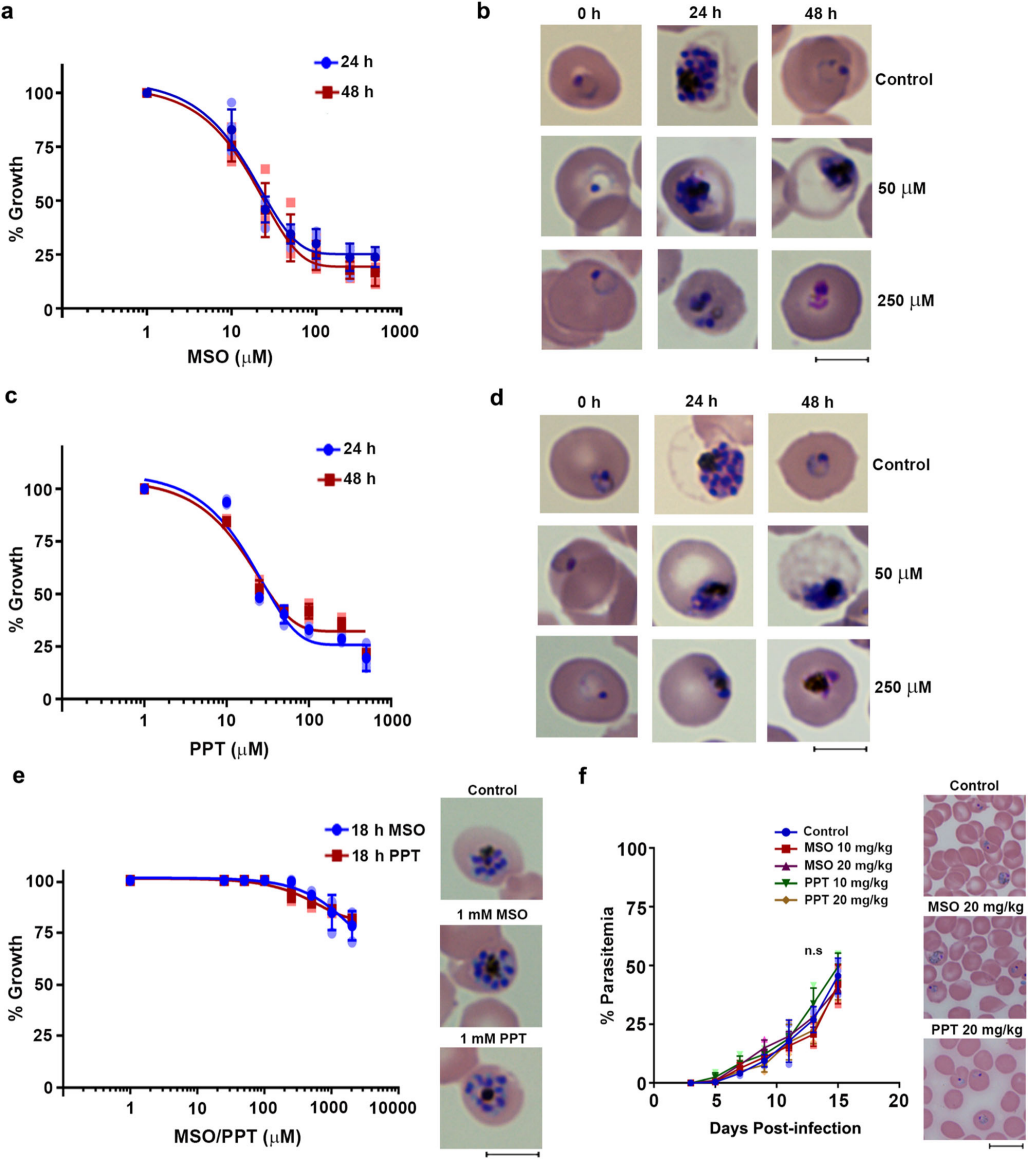
Differential Inhibition of *Pf* and *Pb* parasites by MSO and PPT. **a** Effect of MSO on in vitro cultures of *Pf* in RPMI^-gln^ medium. *n* = 4 independent experiments. **b** Giemsa-stained images of *Pf* parasites treated with MSO. **c** Effect of PPT on in vitro cultures of *Pf* in RPMI^-gln^ medium. *n* = 3 independent experiments. **d** Giemsa-stained images of *Pf* parasites treated with PPT. **e** Effect of MSO and PPT on in vitro single-cycle cultures of *Pb* maintained in RPMI^-gln^ medium. Giemsa-stained images of MSO and PPT-treated *Pb* schizonts are provided for 1 mM concentration. A growth assessment was carried out based on ^3^H-hypoxanthine uptake and verified by Giemsa-stained smears. *n* = 3 independent experiments. **f** Effect of MSO and PPT on in vivo *Pb* growth in Balb/c mice. *Pb* infections were initiated by injecting 10^5^ parasites intraperitoneally on day 0. Mice were treated with the respective doses of MSO and PPT for four consecutive days starting from day 4. *n* = 3 different mice. Giemsa-stained images of MSO and PPT-treated *Pb* parasites are provided for 20 mg/kg treatment. Images for Giemsa-stained parasites were captured using 100x objective. Scale bar = 5 μM. For **a**, **c**, **e**, and **f** the data represent mean ± SD. Source data are provided as a Source Data file.

The results of transgenic *Pf* and *Pb* parasites were further validated by examining the effect of MSO and PPT on in vitro growth of *Pf*, and in vitro and in vivo growth of *Pb*. It is known that the presence of glutamine in culture medium can compete with the cellular uptake of MSO and PPT because of its structural similarity^42–44^. Therefore, we preferred to examine the effect of MSO and PPT on in vitro growth of *Pf* in the presence and absence of glutamine. MSO and PPT addition could inhibit the growth of *Pf* asexual stages in the presence and absence of glutamine. The IC_50_ values for MSO and PPT inhibiting *Pf* growth in RPMI^-gln^ medium were in the range of ~25 μM and Giemsa-stained smears showed the presence of stressed, arrested, pyknotic and dead parasites (Fig. 8a–d). In RPMI^Pgln^, the IC_50_ values obtained for MSO and PPT were almost 10 times higher (Supplementary Fig. 5f, g). On the contrary, MSO and PPT did not inhibit the growth of *Pb* in single-cycle in vitro cultures. Even at a 2 mM concentration of MSO, there was only a slight inhibition of around 20% in RPMI^-gln^ (Fig. 8e), and there was no inhibition in the presence of glutamine (Supplementary Fig. 5h). Similar effect was observed in vivo when *Pb*WT-infected mice were treated with a dosage of MSO and PPT, as high as 20 mg/kg (a non-lethal, sub convulsive dose) per day for four days starting from day 4 post-infection. The growth of *Pb*WT in the treated mice was similar to that of untreated control with no significant changes in the mortality of mice (Fig. 8f). Besides confirming the requirement of GS in *Pf*, the data obtained with the chemical inhibition studies indicate the effect of targeting GS for *Pf* infections.

### Targeting GS affects the protein synthesis in *Pf*

**Fig. 9:**
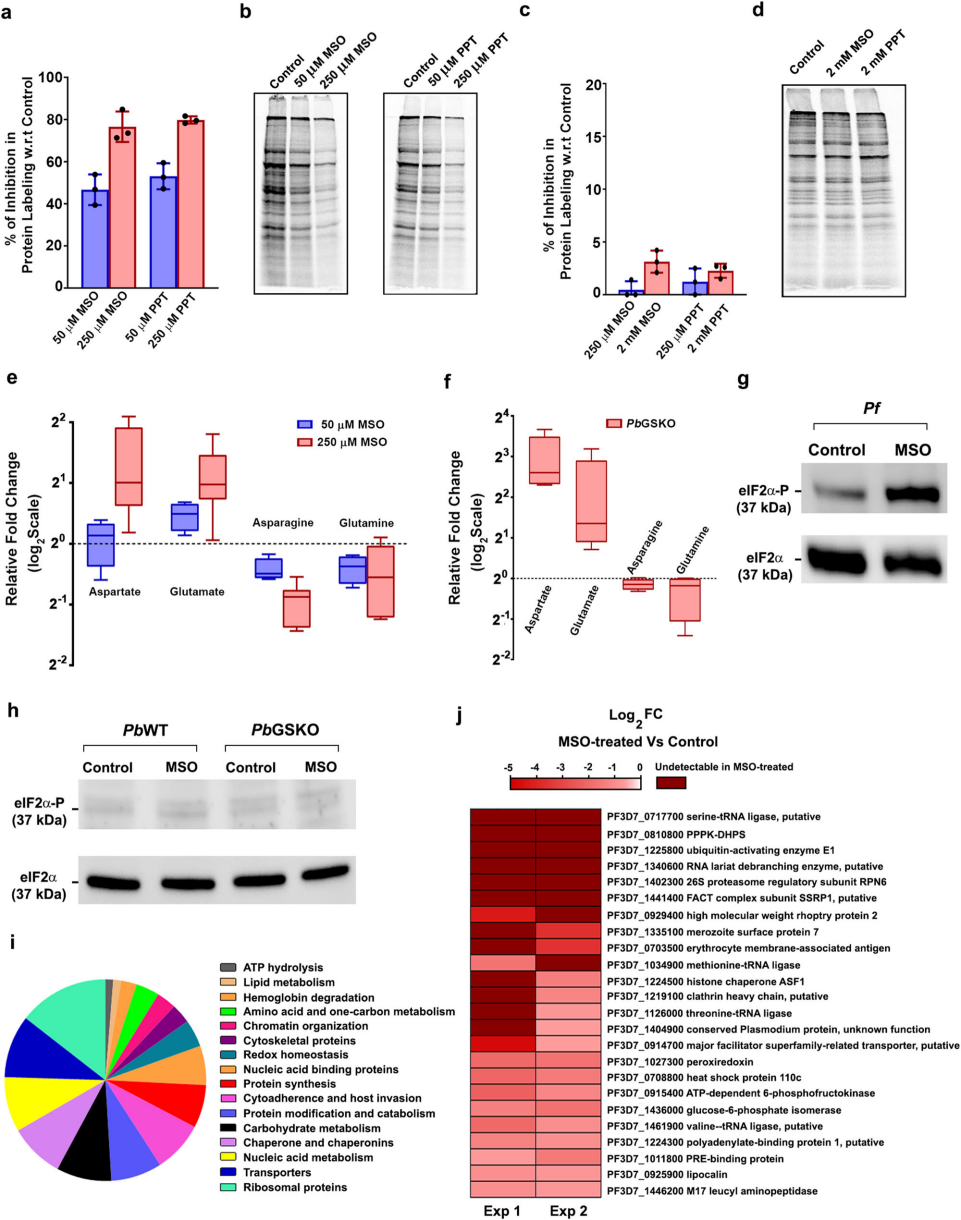
Targeting GS in *Pf* affects asparagine levels and protein synthesis. **a**, **c** In vitro metabolic labeling of *Pf* (**a**) and *Pb* (**c**) cultures with [^35^S]-Methionine and -Cysteine. Percentage of inhibition (mean ± SD) for treated parasites based on ^35^S counts with respect to solvent control is shown. **b**, **d** SDS-PAGE analysis of protein labeling for *Pf* (**b**) and *Pb* (**d**) parasites. Phosphorimager scan was performed after overnight exposure. *n* = 3 independent experiments. **e** Quantification of aspartate, glutamate, asparagine and glutamine levels in *Pf* cultures treated with 50 (*n* = 4) and 250 μM (*n* = 6) MSO. In vitro experiments were carried out in RPMI^-gln^ medium. **f** Quantification of aspartate, glutamate, asparagine and glutamine levels in *Pb*GSKO parasites (*n* = 4). For **e** and **f** relative fold changes of the amino acids with respect to control are plotted after normalizing them with the levels of serine, threonine, histidine, arginine and tyrosine. Box and whisker plots display minimum/maximum points (whiskers), 25^th^/75^th^ percentile (boxes) and median (center line). **g**, **h** Western analysis of total and phosphorylated eIF2α levels in *Pf* and *Pb* parasites, respectively. 10 ml of synchronized *Pf* cultures having rings were treated in vitro with 250 μM MSO for 6 h in RPMI^-gln^ medium. Shorter treatment of *Pf* rings was preferred since eIF2α phosphorylation occurs at late asexual stages. *n* = 4 independent experiments. For *Pb*, infected mouse blood containing rings was incubated in vitro with 250 μM MSO for 6 h in RPMI^-gln^ medium. *n* = 2 independent experiments. **i** Functional classification of downregulated proteins in MSO-treated *Pf* parasites. **j** List of downregulated asparagine-rich proteins in MSO-treated *Pf* parasites. Proteins containing ≥10% asparagine or at least one asparagine repeat with 5 or more asparagine residues were considered asparagine-rich. For **i** and **j** proteins identified in both the untreated controls of two independent experiments and either undetectable or significantly downregulated (≥1.5 fold) in MSO-treated *Pf* parasites are represented. List of other downregulated proteins is provided in Supplementary Fig. 7a. Source data and silver-stained gels representing phosphorimager scans are provided as a Source Data file.

To gain insights on *Pf*-specific requirement of GS, we examined the metabolic labeling of proteins using [^35^S]-Methionine and -Cysteine in in vitro cultures of *Pf* and *Pb*. Interestingly, MSO and PPT treatments for only 12 h could inhibit protein synthesis in *Pf*, but not in *Pb*. While a significant 40–50% inhibition in protein synthesis of *Pf* was observed at 50 μM MSO and PPT, the inhibition was close to 80% at 250 μM in RPMI^-gln^ medium (Fig. 9a, b). In RPMI^Pgln^ medium, MSO and PPT could lead to ~50% inhibition in protein synthesis at 1 mM concentration (Supplementary Fig. 5i, j). In case of *Pb*, no such inhibition was observed even in RPMI^-gln^ medium at 2 mM MSO and PPT (Fig. 9c, d). For further analysis, we performed our studies with MSO in RPMI^-gln^ medium. Examination of free glutamine levels in *Pf* parasites treated with MSO in RPMI^-gln^ medium with respect to the untreated parasites, and *Pb*GSKO parasites isolated from mice with respect to *Pb*WT parasites showed a similar decrease in the glutamine levels (Fig. 9e, f). However, the free asparagine levels were reduced to the extent of 1.5–2.0 fold in *Pf* parasites treated with MSO with respect to the untreated control (Fig. 9e), and almost remained unaltered in *Pb*GSKO parasites in comparison with *Pb*WT parasites (Fig. 9f). Since *Pf* proteins are rich in asparagine^45,46^, we examined whether GS inhibition by MSO in *Pf* can lead to eIF2α phosphorylation - a molecular signature of amino acid deprivation leading to the inhibition of protein synthesis. Interestingly, a short-term exposure of in vitro cultures to 250 μM MSO for 6 h could lead to prominent phosphorylation of eIF2α in *Pf* (Fig. 9g), and no such phosphorylation could be detected either in *Pb* WT or GSKO parasites (Fig. 9h). To investigate the reflection of eIF2α phosphorylation on *Pf* proteome, we performed proteomics analysis for MSO-treated *Pf*3D7 parasites. Around 150 *Pf* proteins associated with various metabolic and cellular functions, cytoadherence and host invasion, Hb degradation, etc., were significantly reduced or undetectable in MSO-treated *Pf*3D7 parasites from two independent experiments. This, in turn, represented >40% of the total proteins identified in the control, suggesting an overall decrease in protein synthesis (Supplementary Fig. 6a; Fig. 9i, j; Supplementary Data 2). This also included important asparagine-rich proteins such as tRNA ligases, components of RNA processing and protein degradation pathways, lipocalin associated with hemozoin formation and antimalarial drug sensitivity^47^, heat shock protein 110c essential for stabilizing the asparagine repeat-rich parasite proteins^46^ etc. (Fig. 9j; Supplementary Data 2). Only ten proteins were found to be significantly upregulated in MSO-treated *Pf*3D7 parasites (Supplementary Fig. 6b; Supplementary Data 2).

Given the primitive role of TCA cycle and mitochondrion in the asexual stages of *Plasmodium*, the other major functions of glutamine that could be considered for growth inhibition are nucleotide and hexosamine biosynthesis. To examine the effect of MSO treatment on nucleotide biosynthesis, we performed ^32^P-orthophosphoric acid and [^35^S]-Methionine and -Cysteine radiolabelling of in vitro *Pf* cultures, and assessed RNA and protein synthesis in parallel for synchronized rings and trophozoites. While both the stages showed inhibition in protein and RNA synthesis, inhibition in protein synthesis was comparatively higher (Supplementary Fig. 7a, b). The levels of nucleotides were comparable between untreated and MSO-treated *Pf* parasites (Supplementary Fig. 7c). In addition, we supplemented *Pf* cultures with orotic acid (OA) and glucosamine (GlcN) - the respective committed precursors of pyrimidine nucleotide and hexosamine biosynthesis. OA supplementation up to 30 μM and GlcN supplementation up to 2 mM concentrations are well tolerated in *Pf* cultures without much inhibition of parasite growth^48,49^. However, the supplementation of *Pf* cultures with OA or GlcN independently, and the combination of both could not rescue MSO inhibition by more than 20% even in 24 h and 48 h (Supplementary Fig. 8a, b). The supplementation of OA and GlcN at concentrations above 30 μM and 2 mM, respectively, did not rescue further and there was actually a mild growth inhibition. Since MSO can also inhibit γ-glutamylcysteine synthetase to certain extent^26^, enzyme catalyzing the first step in glutathione synthesis, we examined the levels of glutathione in MSO-treated *Pf* parasites. However, no significant differences could be observed between treated and untreated *Pf* parasites (Supplementary Fig. 8c), and similar results were obtained for *Pb*WT and *Pb*GSKO parasites (Supplementary Fig. 8d), suggesting that the inhibition or deletion of GS does not affect the levels of glutathione in *Pf* or *Pb* parasites, respectively. More importantly, the experiments of chemical inhibition and cKS were all carried out in RPMI^-gln^ or RPMI^Pgln^ medium containing ~5 times higher asparagine levels (378 μM) than plasma (40–80 μM), suggesting that the extracellular asparagine could not compensate for the endogenous asparagine deficit in *Pf* caused by GS inhibition. Even at a very high extracellular concentration of 5 mM, asparagine could not restore the growth inhibition of MSO in *Pf* (Supplementary Fig. 8e) and rescue GS-mislocalized *Pf*GS^cKS^ parasite growth (Supplementary Fig. 8f). This in turn correlates with the absence of detectable transporters for aspartate and asparagine in apicomplexan parasites^4^. All these findings suggest that *Pf*-specific requirement of GS could be due to a unique functional requirement of glutamine for the synthesis of asparagine that is crucial for asparagine-rich proteins in *Pf*, but not in *Pb*.

### Effect of GS inhibitors on *P. vivax* (*Pv*) and ART-resistant *Pf*Cam3.I^R539T^ parasites

**Fig. 10:**
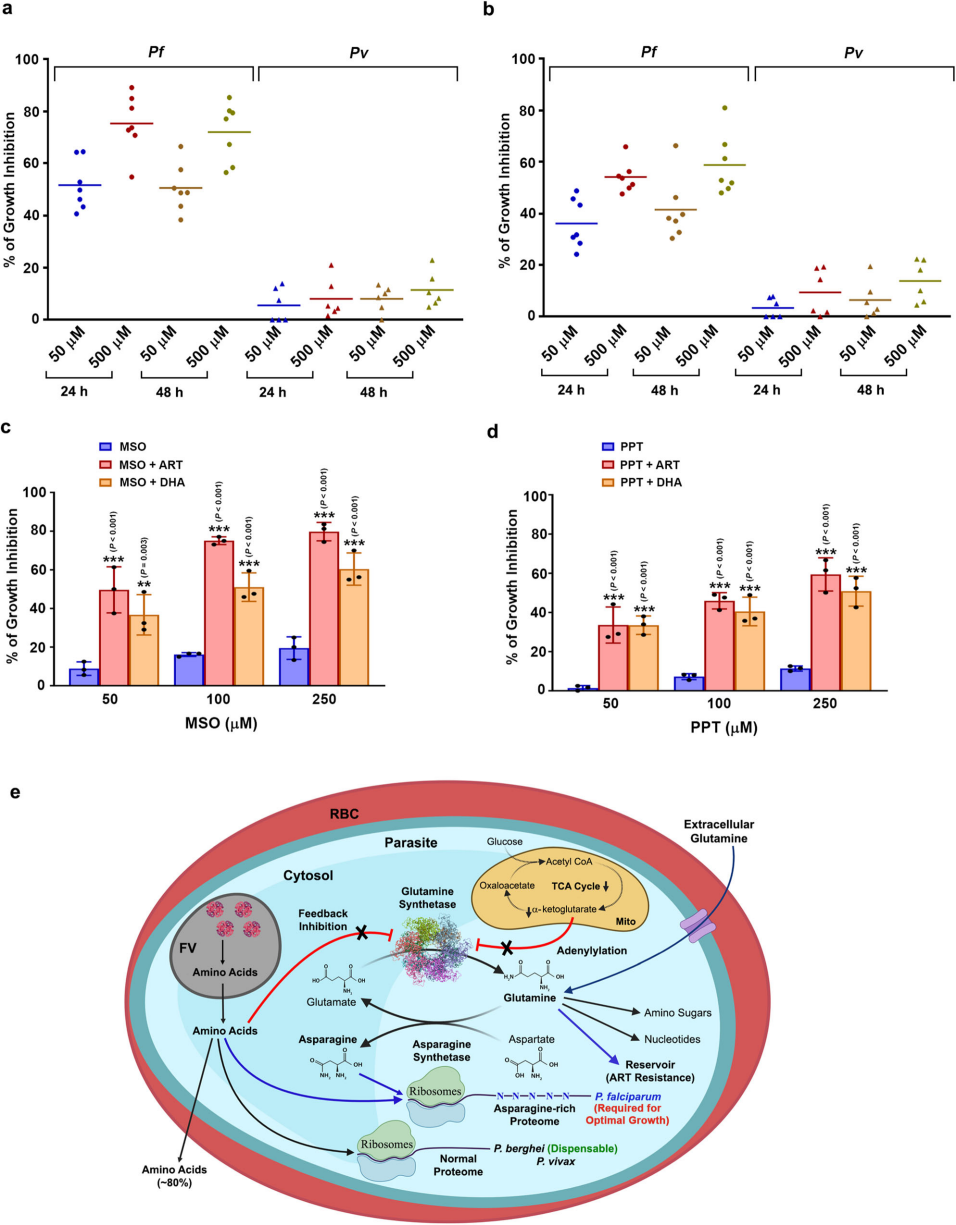
Effect of MSO and PPT on *Pf* and *Pv* clinical isolates and ART-resistant *Pf*Cam3.I^R539T^ strain. **a** Effect of MSO on in vitro growth of *Pf* (*n* = 7) and *Pv* (*n* = 6) clinical isolates in RPMI^-gln^. **b** Effect of PPT on in vitro growth of *Pf* (*n* = 7) and *Pv* (*n* = 6) clinical isolates in RPMI^-gln^. A growth assessment was carried out based on ^3^H-hypoxanthine uptake and verified by Giemsa-stained smears. **c** Effect of ART/DHA and MSO combination on the growth of ART-resistant *Pf*Cam3.I^R539T^ parasites in RSA were performed with RPMI^-gln^ medium. The percentage of growth inhibition of ART/DHA and MSO combination treatment was determined at 96 h with respect to ART/DHA-treated parasites. The percentage of growth inhibition of MSO treatment alone was determined at 96 h with respect to untreated parasites. **d** Effect of ART/DHA and PPT combination on the growth of ART-resistant *Pf*Cam3.I^R539T^ parasites in RSA were performed with RPMI^-gln^ medium. The percentage of growth inhibition of ART/DHA and PPT combination treatment was determined at 96 h with respect to ART/DHA-treated parasites. The percentage of growth inhibition of PPT treatment alone was determined at 96 h with respect to untreated parasites. Growth assessment was carried out based on ^3^H-hypoxanthine uptake and verified by Giemsa-stained smears and flow cytometry. (mean ± SD; **P < 0.01, ***P < 0.001, Two-way ANOVA) *n* = 3 independent experiments. **e** Model depicting the distinct evolution of *Plasmodium* GS and its significance in *P. falciparum*. Lack of feedback inhibition by amino acids and absence of adenylylation in *Plasmodium* GS are represented. Blue arrows highlight the requirement of GS in supporting *Pf* asparagine-rich proteome and the role of glutamine as a reservoir of nitrogen source in ART-resistance. FV- food vacuole; Mito - mitochondrion; RBC - red blood cell. The model was created with BioRender.com.

We next sought to examine the requirement of GS in *Pf* clinical samples and investigate whether the non-essentiality of GS in *Pb* could be corroborated with *Pv* - another human parasite whose proteins are not asparagine-rich. For this, we collected the blood samples from *Pf*- and *Pv*-infected patients and incubated them in in vitro cultures without or with glutamine and assessed their growth in the presence of MSO and PPT. MSO and PPT could inhibit the growth of *Pf* clinical samples to the extent that was observed for *Pf*3D7 strain. However, as observed for *Pb*, MSO, and PPT caused only a ~ 10% inhibition in the growth of *Pv* clinical samples (Fig. 10a, b; Supplementary Fig. 9a, b). This in turn suggested that GS requirement is restricted to *Pf* mainly due to the asparagine-rich nature of its proteins containing asparagine repeats. It has been shown that GS levels are upregulated in ART-resistant *Pf* parasites during ART exposure favoring them to synthesize glutamine for nitrogen storage and prepare for starvation^50^. Therefore, our next interest was to examine whether inhibiting GS by MSO can be effective in ART-resistant strain. For this, we performed ring-stage survival assay (RSA) using tightly synchronized rings of ART-resistant *Pf*Cam3.I^R539T^ strain. ART or dihydroartemisinin (DHA; 700 nM) exposure for 6 h led to viable parasites in *Pf*Cam3.I^R539T^ cultures after 72 h, but not in ART-sensitive *Pf*3D7 cultures. Interestingly, the exposure of ART or DHA in combination with MSO for 6 h led to ~50–80% reduction in viable parasites at 50–250 μM concentrations of MSO in RPMI^-gln^ in vitro cultures with respect to the treatment with ART or DHA alone. The exposure of *Pf*Cam3.I^R539T^ rings to MSO alone for 6 h could only lead to 10–20% inhibition with respect to the untreated control (Fig. 10c). A similar inhibition pattern was observed for RPMI^Pgln^ in vitro cultures wherein, 50% inhibition was observed at ~500 μM MSO (Supplementary Fig. 9c). PPT could also reduce the viability of *Pf*Cam3.I^R539T^ rings in combination with ART or DHA, although PPT was slightly less effective than MSO (Fig. 10d; Supplementary Fig. 9d). These data suggest the effect of inhibiting GS in ART-resistant *Pf* strain.

## Discussion

GS is an ancient ubiquitous enzyme known for extensive evolutionary adaptations and diverse regulatory mechanisms^51, 52^. It plays a pivotal role in the nitrogen metabolism of prokaryotes and eukaryotes. Glutamine, the product of GS, regulates protein turnover and homeostasis, apoptosis, autophagy, pH homeostatis, cell signaling etc. It is required for nucleotide and hexosamine biosynthesis. Glutamine is also a gluconeogenic and lipogenic precursor, and it serves as an anaplerotic carbon source for TCA cycle controlling cell growth, proliferation and function^15^. A plethora of functions played by glutamine renders it a versatile and the most abundant amino acid, not only in humans but also in mosquitoes. While the coevolution of malaria parasite with human and mosquito hosts, and the parasitic niche that is conducive for acquiring amino acids from the host, have resulted in the loss of de novo pathways for amino acid biosynthesis, the parasite has surprisingly retained a gene for GS. We demonstrate that *Plasmodium* GS is enzymatically active and it has evolved as a unique type I GS that cannot be classified under α or β subtypes. It is neither regulated by adenylylation nor feedback inhibited by the end products of glutamine metabolism that controls GS1β activity (Fig. 10e). In agreement with this, the three key proteins essential for GS adenylylation and deadenylylation cycle - adenylyltransferase (ATase;*glnE*), bifunctional uridylyltransferase/uridylyl-removing enzyme (UTase/UR;*glnD*) and the signal transduction protein pII^36^ are also absent in the malaria parasite^17^. Further, parasite GS is not feedback inhibited by glutamine that strongly inhibits GS Iα activity. AMP is the only metabolite that showed moderate inhibition.

We propose that parasite GS has evolved to adapt to the asexual stage metabolism (Fig. 10e). The asexual stages acquire up to 75% of host Hb and degrade it in the FV, releasing amino acids in millimolar concentrations that are far more than the requirement for protein synthesis. The parasite utilizes approximately one-fifth of Hb-derived amino acids and effluxes the surplus. This includes amino acids such as glycine, serine, and alanine that inhibit GS of other organisms^53–56^. Therefore, the absence of feedback inhibition by amino acids could have rendered parasite GS withstanding its activity in the cytosol where there is a continuous release of amino acids from FV. Likewise, the lack of regulation by adenylylation could be a metabolic adaptation. α-ketoglutarate, a TCA cycle intermediate, is a key allosteric regulator of GS and low intracellular concentrations of α-ketoglutarate inactivate GS by bringing out adenylylation through pII/ATase^36^. It is known that the asexual stages support their rapid growth and proliferation by continuously deriving ATP through glycolysis and the flux of carbon skeletons from glucose into the TCA cycle is minimal^14^. Most of the TCA cycle enzymes could be deleted without any significant effect on asexual stages. However, TCA cycle is essential for mosquito stage development^57^. The lack of regulation by adenylylation could have provided the flexibility for parasite GS to function in all the life cycle stages irrespective of its energy metabolism being supported by glycolysis or oxidative phosphorylation. It is worthwhile to mention that *Hp* lacking adenylylation of GS is also known for mixed acid fermentation pathways involving alternate electron acceptors and reduced TCA cycle activity^58^. The preference of parasite GS for Mg^2+^ over Mn^2+^ could also be correlated with the abundant Mg^2+^ levels (39–59 μg/g) in RBCs than Mn^2+^ (0.009–0.033 μg/g), and the presence of multiple putative magnesium transporters in the parasite genome^17^.

Another interesting feature of *Plasmodia* GS is the presence of two characteristic peptide inserts. We show that the first peptide insert occupying dodecamer channel contributes to the stability of *Pf*GS at febrile temperatures. *Plasmodium* parasites show extensive adaptations to febrile temperatures, with *Pf* heat shock protein 110 stabilizing the asparagine repeat-rich parasite proteins and *Pf*AP2-HS transcription factor protecting the parasites^46,59^. Unlike the first peptide insert, deletion of second insert present near the active site renders parasite GS completely inactive. It would be of interest to examine the compensatory structural rearrangements that have occurred in *Plasmodium* GS to accommodate this insert and their impact on GS activity. It is also possible that this insert is responsible for the lack of feedback inhibition by amino acids. Further structural studies are required to understand its conformational flexibility in the presence of substrates, transition-state analogs, and feedback inhibitors. Altogether, our findings highlight the distinct evolution of parasite GS and shed insights on unique biochemical and structural features of parasite GS that complement the metabolic signature and febrile niche of asexual stage parasites.

Another important finding of this study is the recognition of species-specific differences in the requirement of *Plasmodium* GS. By combining reverse genetics with chemical inhibition studies and by correlating the results with clinical samples, we show that GS is required for optimal *Pf* growth, but non-essential for *Pb* and *Pv*. GS deletion in *Pb* does not affect the asexual stage parasite growth or the disease outcome in mice. *Pb*GSKO parasites can complete the transmission cycle and the only significant effect is a ~ 40% reduction in sporozoite formation. The dispensable nature of *Pb*GS is also reported in high-throughput barcoded *Pb* mutant studies^60,61^. More importantly, GS deletion does not alter the intracellular levels of asparagine in *Pb*, and treatment of *Pb*WT parasites with MSO or PPT does not affect protein synthesis. In contrast, chemical inhibition of *Pf* with MSO and PPT shows significant reduction in the asparagine levels and a clear-cut inhibition of protein synthesis within 12 h, suggesting the requirement of GS for asparagine and protein synthesis in *Pf*. Our in vitro studies with MSO and PPT are performed in the presence and absence of glutamine, since glutamine is known to compete with their uptake. We have also substantiated the requirement of GS in *Pf* asexual and sexual stage development by cKS approach. For essential metabolic enzymes that are abundantly expressed, even 90–95% knockdown does not lead to significant growth defects, and 5–10% of enzyme activity from the leftover protein seems to be adequate for normal parasite growth^62^. In case of *Pf*GS^cKS^, there was almost 90% growth inhibition suggesting the important role played by GS in *Pf*. This is in agreement with the low mutant fitness score (MFS) of *Pf*GS observed in piggyBac mutagenesis^17^. Despite being predicted as non-essential with high mutagenesis index score, low MFS suggests the relatively low abundance of GS mutants in the pool, indicating a high fitness cost of the GS mutation. We have extended our findings on *Pb* to *Pv* by demonstrating that in vitro treatment of MSO and PPT does not cause significant growth inhibition in *Pv* clinical samples. While the results obtained with *Pb* do not suggest significant differences in the RBC versus reticulocyte preference upon GS deletion, the contribution of reticulocyte tropism to the refractoriness of MSO and PPT inhibition in *Pv*, if any, needs further investigations.

Glutamine is limited in Hb^6^ representing ~1.3% of the total amino acids present in α and β chains of Hb, in comparison to ~4–5% of glutamate or aspartate. Our findings clearly suggest that Hb-derived and extracellular glutamine are inadequate to support optimal *Pf* growth in asexual and sexual stages when the endogenous GS activity is compromised. With a very high AT content of 80.6% in its genome, *Pf* has a distinct evolutionary path derived from *Laverania* subgenus infecting African Great Apes. The rest of the human parasites - *vivax*, *malariae*, *ovale* and *knowlesi* represent *Plasmodium* (*non-Laverania*) subgenus and their AT content of genome varies between 60–75%. The 23 Mb AT-rich genome of *Pf* encodes more than 5000 proteins and the unique feature of *Pf* proteins is their asparagine-rich nature. Many *Pf* proteins expressed in different developmental stages have asparagine repeats with an average length of 37 residues that span across 30% of the proteome^46^. While AT-rich genome of *Pf* could be attributed to asparagine-rich nature, encoded mainly by AAT, such repeats are not present in other species like *malariae* and *ovale* with a reasonably high AT content. The evolutionary significance of asparagine-rich *Pf* proteome remains unclear. However, asparagine repeats present in the Low Complexity Region (LCR) are visualized to act like ‘sponges’ for asparaginyl-tRNA, rendering a rate-limiting function in *Pf* protein synthesis^46^. Despite having a high requirement of asparagine, *Pf* can be maintained in vitro in an amino acid-free RPMI supplemented only with isoleucine. Therefore, *Pf* should have a biosynthetic machinery to support its high asparagine requirement for protein synthesis. While our results show that *Pf*GS supplies glutamine as an amide donor for asparagine synthesis, they do not exclude the requirement of glutamine for other metabolic pathways as evident from the decreased RNA labeling in MSO-treated *Pf*. Requirement of glutamine for other metabolic pathways may eventually become prominent at prolonged duration of MSO treatment although inhibition of protein synthesis occurs early. Our results also suggest that *Pb* and *Pv* parasites can satisfy glutamine requirement for protein synthesis and other metabolic pathways in the absence of endogenous GS activity through hemoglobin degradation and/or extracellular sources. Deletion or inhibition of endogenous GS does not have a significant impact on *Pb* or *Pv* growth. In summary, asparagine-rich proteome imposes a selective burden in *Pf* for glutamine besides its requirement for other metabolic pathways.

Finally, we show the effect of inhibiting GS in ART-resistant *Pf* strain. The exposure of ART-resistant *Pf*Cam3.I^R539T^ strain to the combination of ART/DHA with MSO/PPT for a short duration of 6 h leads to a substantial decrease in the parasite survival. Since ART-resistant parasites depend on glutamine as a nitrogen reservoir^50^ and exhibit fitness loss in amino acid and nutrient limitation conditions^63,64^, we propose that the asparagine-rich nature of *Pf* proteins and the requirement of glutamine as a nitrogen source for asparagine and protein synthesis can be explored for ART-resistance. GS is being examined as a target for *Mt* infections and structural analogues of MSO, PPT, and ATP have been evaluated for their potential to inhibit *Mt*GS^24,36^. Targeting GS is also being attempted for cancer therapy since glutamine metabolism plays an important role in cancer cells and GS levels are upregulated in certain types of cancer^65^. We believe that the unique structural features of *Plasmodium* GS and its unusual type I nature can serve as a platform for developing *Plasmodium*-specific GS inhibitors. The other challenges such as the efficient uptake of GS inhibitors and their efficacy at clinically relevant doses with favorable therapeutic index have to be addressed. Asparagine synthetase (AS) mutation also seems to impose a high fitness cost in *Pf* as evidenced by low MFS^17^ and therefore, *Pf*AS can be explored as a target. We have already shown that AS can be deleted in *Pb*^8^ and it would be of interest to examine its requirement in *Pf*. Like GS, AS is being explored for cancer therapy, and adenylated sulfoximines and methylsulfoximines are shown to be potential inhibitors of AS^66^. While an antimalarial for all the species of *Plasmodium* is preferable, the inclusion of a *Pf*-specific drug in the existing ACTs would be helpful to address ACT failure and fulfil the requirement of an additional partner in ACTs for *Pf* infections^67^. In such combinations, the primary drugs will clear the other species. Nevertheless, the reason for why other *Plasmodium* species lacking asparagine-rich proteins have retained GS and whether *Pf*GS can be targeted for the mosquito and liver stages require further studies. The metabolic transactions in *Pf* that facilitate the channeling of glutamine for protein synthesis need to be addressed.

## Methods

### Ethics statement

The studies involving mice were approved by Institutional Animal Ethics Committee (ILS/IAEC-69-AH/AUG-16) and the experiments were carried out according to the national guidelines framed by “The Committee for the Purpose of Control and Supervision of Experiments on Animals (CPCSEA)”. *Pf* and *Pv* clinical samples were collected with the approvals from Institutional Ethics Committee (IEB)/ Institutional Review Board (IRB) of the Institute of Life Sciences (94/HEC/19), Bhubaneswar, and KMC Hospital (IEC:248/2019), Mangalore.

### Homology modeling of *Plasmodium* GS

Three-dimensional structures of *Pv* and *Pb* GS are not available. Therefore, homology modeling of *Pv* and *Pb* GS were carried out using MODELLER v10.1. based on the cryo-EM structure of *Pf*GS (PDB ID: 6PEW). *Pv* and *Pb* GS showed 74.22% and 68.42% identity with *Pf*GS, respectively. We performed alignment of *Pv* and *Pb* GS with the available three-dimensional structures of *Pf* (PDB ID: 6PEW), *St* (PDB ID: 1F1H), *Mt* (PDB ID: 2WGS), and *Hp* GS (PDB ID: 5ZLP) using PROMALS3D. Ten models were built for *Pv* and *Pb* GS, and they were subsequently optimized using the variable target function method (VTFM) with conjugate gradient (CG) algorithm and then refined using molecular dynamics (MD) with simulated annealing (SA). The best models for *Pv* and *Pb* GS were chosen based on the molpdf scores. A close inspection of the generated structures revealed that the loops present in the first peptide inserts of *Pv* (Gly174-His196) and *Pb* GS (Va174-Ser206) were having knotting with the same loop of other monomeric units in the final dodecameric structure. Therefore, loop modeling and loop refinement were also performed using MODELLER v10.1. by generating 100 loop models to remove knotting. Each of the generated loops was incorporated in a monomeric unit that was superimposed on the dodecameric structure generated previously. Each of the generated structures was carefully analyzed to check for any loop entanglement, and the model that had no loop entanglement or bumping with a nearby monomeric unit was selected. The final model generated was then superimposed on *Pf*GS to examine the differences in C_α_ backbone of the model using PyMOL Molecular Graphics System, Version 1.2r3pre, Schrödinger, LLC. A similar procedure was also followed to model the first peptide insert of *Pf*GS that could not be observed in the cryo-EM structure.

### Cloning, over-expression and purification of recombinant *Pf*, *Pb,* and *E. coli* GS

cDNA sequences of *Pf*GS (PF3D7_0922600) and *Pb*GS (PBANKA_0823500) were retrieved from PlasmoDB (https://plasmodb.org/plasmo/app). cDNA sequence of *E. coli* GS (CAD6154619.1) was retrieved from NCBI GenBank (https://www.ncbi.nlm.nih.gov/genbank/). Total RNA from *Pf*, *Pb* or *E. coli* was isolated using RNeasy Mini Kit (Qiagen, 74104) according to the manufacturer’s protocol. cDNA synthesis was carried out with 1 μg of total RNA using RevertAid Reverse Transcriptase (Thermo Fisher Scientific, EP0442), followed by PCR with Phusion High-Fidelity DNA Polymerase (New England Biolabs, M0530). The following were the forward (F) and reverse (R) primers used: *Pf*GS (F): 5’-GCCAGGATCCATGAAGTCCGTGAGTTTTTCAAATAATGC-3’; *Pf*GS (R): 5’-GCCCAGATCTCTAACATTCATAATATAAGTGATAATCATAAGCG-3’; *Pb*GS (F): 5’-GCCAGGATCCATGAAATTTATCAGTTTTTCGAACCCAATCG-3’; *Pb*GS (R): 5’-GCCCAGATCTTTAAGATCCATAATACATGATAAATTCAGAAGG-3’; *E. coli* GS (F): 5’-GCAACTCGAGATGTCCGCTGAACACGTACTGACG-3’; and *E. coli* GS (R): 5’- GCAAAAGCTTTTAGACGCTGTAGTACAGCTCAAACTCTAC-3’ (Sigma-Aldrich). The restriction sites used for cloning are underlined. cDNA products were digested with the respective restriction enzymes and cloned into pRSETA plasmid (Thermo Fisher Scientific). Recombinant protein expressions were carried out in *E. coli* Rosetta2DE3pLysS strain (Novagen). In brief, *E. coli* Rosetta2DE3pLysS cells transformed with recombinant plasmids were grown to an A_600_ of 1.0 at 30 °C and the protein induction was carried out at 18 °C for 12 h using 1 mM isopropyl-β-D-thiogalactoside (IPTG) (MP Biomedicals, 11IPTG0001). The recombinant proteins fused with 6xHis tag were purified using Ni^2+^-NTA Agarose resin (Qiagen, 30210). In brief, the bacterial cell pellets expressing the recombinant proteins were resuspended in lysis buffer containing 50 mM Tris pH 8.0, 500 mM NaCl, 20% glycerol, 0.01% Triton X-100, 1 mM dithiothreitol and protease inhibitors, sonicated and centrifuged at 43,000 *g* for 1 h. The supernatant was separated and loaded onto a column packed with Ni^2+^-NTA resin and washed sequentially with lysis buffer containing 1, 10, and 50 mM imidazole. The recombinant proteins were then eluted with lysis buffer containing 150 mM imidazole. The purified protein was then dialyzed against 50 mM Tris pH 8.0, 50 mM NaCl and 20% glycerol. Protein estimation was carried out using Pierce BCA Protein Assay Kit (Thermo Fisher Scientific, 23225). The total yield of recombinant GS was around 0.25–0.50 mg per litre of bacterial culture.

### Enzyme assays

*Plasmodium* and *E. coli* GS enzyme assays were carried out by performing HPLC analysis for glutamine formation as well as by quantifying the release of inorganic phosphate (P_i_)^68,69^. The enzyme assays were carried out at 37 °C for 1 h in a total volume of 25 μl and the assay mixtures contained 50 mM Tris buffer pH 8.0 with 4 mM glutamate, 50 mM NaCl, 50 mM NH_4_Cl, 10 mM ATP and 50 mM MgCl_2_ and 0.5–1.0 μg of recombinant protein. The amino acids were extracted by vortexing the assay mixtures with 50 μl of water and 375 μl of acetonitrile, followed by incubation in ice for 30 min and centrifugation at 20,000 *g* for 20 min at 4 °C. The supernatant was collected, lyophilized and dissolved in 50 μl of water. 2.5 μl of the sample was subjected to pre-column derivatization with OPA reagent (Agilent, 5061–3335) by programming the auto-sampler and the separation of amino acids was carried out in Agilent 1260 Infinity HPLC System (Agilent Technologies) using a Poroshell 120 HPH-C18 column (4.6 mm × 100 mm X 2.7 μm) as per the manufacturer’s protocol^70^. Mobile phase consisted of Solvent A: 10 mM Na_2_HPO_4_, and 10 mM Na_2_B_4_O_7_, pH 8.2 and solvent B: methanol: acetonitrile: water (45:45:10 v/v). OPA-derivatized amino acids were detected using 1260 Infinity II Fluorescence Detector with excitation of 340 nm and emission of 450 nm. Amino acid standards (Agilent, 5061–3330) were used to determine the retention time. The standard curves were generated for glutamine and the assay products were also confirmed by spiking with glutamine. For every molecule of glutamine formed, one molecule of ATP is hydrolyzed to ADP and P_i_. Therefore, the release of P_i_ was quantified as an indirect way of measuring glutamine synthesis and the activities were found to be comparable with HPLC assays. For P_i_ assays, the assay mixtures incubated at 37 °C for 1 h were diluted with water to 200 μl, followed by the addition of 30 μl of phosphate reagent of Phosphate Assay Kit (Abcam, ab65622). After vortexing, the absorbance was measured at 650 nm as per the manufacturer’s protocol. The enzyme assays for r*Pf*GS-FKBP-GFP were carried out at 37 °C for 3 h. The enzyme activity of recombinant proteins was also verified after cleaving the 6xHis tag with enterokinase (New England Biolabs, P8070S). For enzyme assays carried out with parasite lysates of *Pf* or *Pb*, parasite pellets isolated by saponin lysis were resuspended in 200 µl of 50 mM Tris pH 7.5 containing 5 mM MgCl_2_, 2 mM dithiothreitol and protease inhibitors, followed by a brief sonication for 15 s. The lysates were then centrifuged at 20,000 *g* for 20 min and the supernatants were separated. The assays were performed with the supernatants as described for the recombinant proteins with the inclusion of 200 µM phosphoenolpyruvate (Sigma-Aldrich, P0564) and 6 Units of pyruvate kinase (Sigma-Aldrich, P7768) to ensure the regeneration of ATP in the parasite lysates^71^. The reaction mixtures were incubated at 37 °C for 6 h and the amino acids were extracted with acetonitrile as mentioned above for HPLC assays. For inhibition studies of recombinant proteins with MSO (M5379, Sigma) and PPT (45520, Sigma), recombinant proteins were pre-incubated with the respective inhibitors, ATP & MgCl_2_ at 37 °C for 20 min, followed by the addition of NH_4_Cl and glutamate, and subsequent incubation at 37 °C for 1 h. Feedback inhibition assays were carried out with 1 mM and 5 mM concentrations of glycine, alanine, serine, histidine, tryptophan, glutamine and AMP. For the mixture of all six amino acids at 5 mM concentrations, tryptophan alone was used at 2.5 mM concentration due to its limited solubility. Individual glutamate- and protein-omitted reactions were used as controls for all the assays.

### Adenylylation analysis of recombinant and endogenous *Pf* and *Pb*GS

To examine the adenylylation of recombinant *Pf* and *Pb* GS, *E. coli* Rosetta2DE3pLysS strain transformed with the respective recombinant plasmid was grown at 30 °C for 6 h to an A_600_ of 0.4-0.5 in LB medium containing 10 mM glutamine, followed by induction with 1 mM IPTG at 30 °C for another 6 h. Recombinant *Pf* and *Pb*GS protein inductions were successful at 30 °C, except for the total yield of soluble protein was approximately less by one third in comparison with protein induction at 18 °C. Recombinant *E. coli* GS protein induction was also carried out at identical conditions and the purification of all the three recombinant proteins was carried out in a similar fashion as mentioned above. Western blot analysis was carried out with AMP-Tyr specific mouse monoclonal antibodies (1:1000 dilution; clone 1G11)^72^. In brief, nitrocellulose membrane was blocked with 1X Roti-Block (Carl Roth, A151.2) for 3 h, followed by overnight incubation with primary antibodies in 1X Roti-Block containing 1 mM MnCl_2_ at 4 °C. After developing, the same blot was stripped and reprobed with mouse monoclonal his-tag antibody (1:4000 dilution; Sigma-Aldrich, H1029). To examine the adenylylation of endogenous GS, parasite lysates were prepared by resuspending *Pf* or *Pb* pellets in 50 mM Tris pH 8.0 containing 100 mM NaCl, 2% glycerol, 0.1 % Triton X-100 and Halt protease inhibitor (Thermo Fisher Scientific, 78438), followed by sonication and centrifugation at 20,000 *g* at 4 °C. The endogenous GS present in the supernatants was immunoprecipitated by allowing it to bind with parasite GS-specific IgG purified from polyclonal sera raised against recombinant parasite GS using protein A magnetic beads (Thermo Fisher Scientific, 88845) and cross-linked with AminoLink Plus coupling resin using Pierce Co-Immunoprecipitation Kit (Thermo Fisher Scientific, 26149). The bound endogenous GS was eluted from cross-linked IgG using Elution buffer pH 2.8 as per the manufacturer’s protocol. Western analysis for endogenous GS adenylylation was carried out as mentioned for recombinant proteins and recombinant *E. coli* GS was used as control in all these experiments.

### Generation of ΔI_1_r*Pf*GS, ΔI_2_r*Pf*GS and ΔI_1_I_2_r*Pf*GS recombinant plasmids

To generate ΔI_1_r*Pf*GS and ΔI_2_r*Pf*GS recombinant plasmids, the two fragments on either side of the respective peptide inserts were PCR amplified individually and cloned sequentially into pRSETA plasmid. For ΔI_1_r*Pf*GS plasmid construct, the following forward (F) and reverse (R) primers were used: *Pf*GS(F): 5’-GCCAGGATCCATGAAGTCCGTGAGTTTTTCAAATAATGC-3’ and ΔI_1_*Pf*GS (R): 5’-GCAACTCGAGACTCGATAAATCATTTTTACAAGAAAATGA-3’ to amplify the first fragment; ΔI_1_*Pf*GS(F): 5’-GCAACTCGAGATTAATGATGATAGTAAAAAAGTAAAGAAAAAA-3’ and ΔI_1_/I_2_*Pf*GS(R): 5’-GCAAGGTACCCTAACATTCATAATATAAGTGATAATCATAAGC-3’ to amplify the second fragment. For ΔI_2_r*Pf*GS plasmid construct, the following forward (F) and reverse (R) primers were used: *Pf*GS(F): 5’-GCCAGGATCCATGAAGTCCGTGAGTTTTTCAAATAATGC-3’ and ΔI_2_*Pf*GS(R): 5’-GCAACTGCAGCGAAAATATGCTTGAAATGTAGAAATGATT-3’ to amplify the first fragment; ΔI_2_*Pf*GS(F): 5’- GCAACTGCAGGAACCACAAGGTATATCTTTTTCTCTTGTCG-3’ and ΔI_1_/I_2_*Pf*GS (R): 5’- GCAAGGTACCCTAACATTCATAATATAAGTGATAATCATAAGC-3’ to amplify the second fragment. The restriction sites used for cloning are underlined. To generate ΔI_1_I_2_r*Pf*GS recombinant plasmid having deletions for both the inserts, three fragments were sequentially cloned. The following forward (F) and reverse (R) primers were used: *Pf*GS (F): 5’-GCCAGGATCCATGAAGTCCGTGAGTTTTTCAAATAATGC-3’ and ΔI_1_*Pf*GS (R): 5’-GCAACTCGAGACTCGATAAATCATTTTTACAAGAAAATGA-3’ for the first fragment; ΔI_1_*Pf*GS (F): 5’-GCAACTCGAGATTAATGATGATAGTAAAAAAGTAAAGAAAAAA-3’ and ΔI_2_*Pf*GS (R): 5’-GCAACTGCAGCGAAAATATGCTTGAAATGTAGAAATGATT-3’ for the second fragment; ΔI_2_*Pf*GS (F): 5’- GCAACTGCAGGAACCACAAGGTATATCTTTTTCTCTTGTCG-3’ and ΔI_1_/I_2_*Pf*GS (R): 5’- GCAAGGTACCCTAACATTCATAATATAAGTGATAATCATAAGC-3’ for the third fragment. ΔI_1_r*Pf*GS and ΔI_2_r*Pf*GS recombinant plasmids were further subjected to site-directed mutagenesis for removing the additional amino acids that were introduced into the GS sequence due to the restriction sites used for cloning. For this, PCR reactions were performed using the following set of primers: 5’- TCATTTTCTTGTAAAAATGATTTATCGAGTTATTTAATTAATGATGATAGTAAAAAAGTAAAGAAA-3’ and 5’- TTTCTTTACTTTTTTACTATCATCATTAATTAAATAACTCGATAAATCATTTTTACAAGAAAATGA-3’ for ΔI_1_r*Pf*GS; 5’-TCAAGCATATTTTCGGAACCACAAGGTATATCTTTTTCTCTTGTCGAAAGTTTAGATGCTTTGGA-3’ and 5’-TATACCTTGTGGTTCCGAAAATATGCTTGAAATGTAGAAATGATTATCTTTACTTTCAAATGGAA-3’ for ΔI_2_r*Pf*GS. All the primers were procured from Sigma-Aldrich. The purified PCR products were then subjected to *DpnI* restriction digestion, followed by transformation. DNA sequencing was carried out to ensure the precise deletion of the peptide inserts in the respective recombinant plasmids. Recombinant protein expressions were carried out as described for r*Pf*GS.

### Analytical size-exclusion chromatography

The oligomeric status of purified r*Pf*GS and ΔI_1_r*Pf*GS was analyzed by analytical size-exclusion chromatography performed with AKTA pure protein purification system using Superose 6 10/300 GL column (Cytiva, 17-5172-01) at 4 °C. The recombinant proteins (0.15 mg) were exposed to 37 °C or 42 °C for 15 min, and subjected immediately to size-exclusion chromatography at a flow rate of 0.15 ml/min in buffer containing 25 mM Tris pH 8.0, 300 mM NaCl and 5% glycerol. Elution fractions of 300 μl volume were collected for the entire run of 3 h and the appropriate fractions showing the eluted protein peaks were analyzed by Western blot using anti-his tag antibodies.

### MD simulations

To check the dynamic behavior of *Pf*GS and ΔI_2_*Pf*GS, MD simulations were performed. Since the active site is formed between two adjacent subunits, we used four subunits - two each from upper and lower hexamers of the dodecameric structure. This strategy was preferred due to the difficulties experienced with the stability of dodecameric *Pf*GS in MD simulations. The preliminary topologies and coordinates for the protein complexes were generated in VMD 1.9.3 using CHARMM34 force field. MD simulations were done using NAMD 2.9. The structures of *Pf*GS and ΔI_2_*Pf*GS were solvated in a rectangular water box (TIP3P) with a buffering distance of 10 Å. Ions (Na^+^ and Cl^-^) were added to ensure the electro-neutrality of the solvated system. Prior to the simulation, each of the system was properly minimized with a stepwise minimization protocol. The water molecules and ions were minimized first, followed by hydrogen atoms and side chains of the protein complex. The side chains were minimized for 100000 steps while the backbone atoms and the bond lengths of hydrogen atoms were kept fixed. Thereafter, all the atoms were allowed to relax freely and the whole system was energy-minimized for 100000 steps with nominal restraints on C_α_ and backbone atoms (10 kcal/mol) to prevent any abrupt change in the structure. Subsequently, an equilibration protocol was followed where the system was heated gradually from 0–310 K in steps of 30 K with a canonical ensemble (NVT). At each step, a 20-picosecond (ps) simulation was run to allow the system to adjust to the temperature. Once the system attained 310 K, isobaric and isothermic ensemble (NPT) was applied for a period of 100 ps with a constant pressure of 1.0 bar using Langevin dynamics. Finally, the applied restraints on C_α_ atoms were removed and the system was equilibrated for 1 ns at 310 K using Langevin piston coupling algorithm. During the whole simulation, the Particle Mesh Ewald (PME) sum algorithm was used to calculate the long-range electrostatic interactions with fixed periodic boundary conditions. The covalent interactions involving hydrogens were constrained using SHAKE algorithm. The production run was done for a time period of 100 ns. The analyzes of the MD trajectories were performed to examine the structure and dynamic behavior of the complexes during MD. The trajectories were analyzed for RMSD, RMSF, hydrogen bonds, and SASA.

### In vitro maintenance of *P. falciparum* cultures

Routine maintenance of *Pf*3D7 cultures were carried out at 37 °C in RPMI-1640 medium (Gibco, 23400-013) containing 0.5% AlbuMAX-II (Thermo Fisher Scientific, 11021037) using O^+ve^ RBCs of 5% hematocrit under 90% N_2_, 5% O_2_ and 5% CO_2_. The parasites were subcultured with fresh RBCs when the culture parasitemia reached around 2–5%. Synchronization of *Pf* cultures was carried out with 5% D-sorbitol (w/v) (Sigma-Aldrich, 240850). Parasites were isolated by treating the infected RBC pellet with equal volume of 0.15% saponin in PBS (w/v) (Sigma-Aldrich, S4521) followed by centrifugation at 10,000 *g* for 10 min at 4 °C. The isolated parasites were washed thoroughly with ice cold PBS for four times to remove the carry-over of proteins from RBC lysates^73^. ART-resistant *Pf*Cam3.I^R539T^ strain was also maintained in a similar fashion. *Pf* strains were cryopreserved by storing them under liquid nitrogen in storage medium containing 28% glycerol, 3% D-sorbitol and 0.65% NaCl and the glycerolyte stocks were revived following the standard protocol recommended by Malaria Research and Reference Reagent Resource Center (MR4). The experiments involving gametocyte formation were carried out with freshly revived glycerolyte stocks that were not passaged for more than five asexual cycles. Giemsa-stained smears were routinely prepared to examine the parasite growth and parasitemia. Parasite growth assessment for experiments was carried out by Giemsa-stained smears, flow cytometry and ^3^H-hypoxanthine uptake^74^. Flow cytometry was performed by staining the infected RBCs with 0.5X SYBR Green I (Thermo Fischer Scientific, S7563) in HBSS buffer pH 7.4 containing 2% FBS (Gibco, 10270106) for 30 min at 37 °C. The data was acquired using BD LSRFortessa (BD). ^3^H-hypoxanthine uptake assays were performed by adding ^3^H-hypoxanthine (American Radiolabeled Chemicals, Inc., ART 0266) (5 μCi per ml culture volume) to the cultures. The labeled cultures were then washed with RPMI-1640 medium, lysed in water, and harvested on glass fibre filters using FilterMate cell Harvester (PerkinElmer). After washing with water, the glass fibre filters were dried and placed in Ultima Gold XR scintillation cocktail (PerkinElmer, 6013111). The radioactive counts were measured using MicroBeta^2^ Microplate Counters (PerkinElmer). Glutamine-free RPMI was procured from Thermo Fisher Scientific (Gibco, 42401-018) and for experiments carried out at physiological concentrations of glutamine, glutamine-free RPMI-1640 was reconstituted with 0.5 mM glutamine (Sigma-Aldrich, G8450). Gametocyte induction for *Pf*3D7 cultures was carried out by crash method as described by treating them with 50 mM N-acetylglucosamine for 72 h when the parasitemia reached around ~6%^75^.

### Routine propagation of *P. berghei* in mice

*Pb* ANKA strain was routinely propagated in 7–8 weeks old male/female Balb/c mice. The experiments were initiated by injecting 10^5^ parasites through intraperitoneal route and the parasite growth was monitored by examining the Giemsa-stained smears prepared from tail vein blood under light microscope. For cerebral malaria experiments, 7–8 weeks old C57BL/6 male/female mice were used and the infections were initiated by injecting 10^5^ parasites. The sex of the mice did not alter the asexual parasite growth, gametocyte development, animal mortality, and disease pathogenesis, and the results were reproducible in both male and female mice. Balb/c and C57BL/6 mice were bred in-house and maintained at the animal house facility of the Institute of Life Sciences, Bhubaneswar, under standard conditions of 25 +/−3 °C temperature, 40–50% relative humidity, and 12 h light / 12 h dark cycle. The development of cerebral pathogenesis in mice was monitored by examining the neurological symptoms such as ataxia, paralysis, convulsion, and coma, and the associated early mortality within day 10 when the peripheral blood parasitemia was around 20%. The integrity of blood-brain barrier was assessed by quantifying the Evans blue extravasation in the brain of infected mice after one hour of injecting 200 μl of 2% Evans blue in PBS intravenously. Evans blue from the brain samples was extracted by incubating them in formamide at 37 °C for 48 h and measuring the absorbance at 620 nm^76^. The isolation of *Pb* parasites from infected RBCs was carried out by saponin lysis.

### Maintenance of *Anopheles stephensi* mosquitoes

*A. stephensi* mosquito colony was reared under standard insectary conditions maintained at 27 °C and 75–80% humidity supported with a 12 h light and dark photo-cycle as described^8,73^. Adult female mosquitoes of 5–7 days old were blood fed on anesthetized Balb/c male/female mice of 8–12 weeks old for the production of eggs. The eggs were collected on damp filter paper and placed for hatching. The larvae were reared in water containing 0.03% (w/v) sea salt (Sigma-Aldrich, S9883), 0.01–0.02% (w/v) yeast powder and liver powder at a density of one larva/ml following the standard procedures. The pupae were separated and kept inside the cages for adult emergence. The adult mosquitoes were fed with 10% sucrose (Merck, 107687) solution containing 0.05% paraminobenzoic acid (Sigma-Aldrich, A9878).

### Immunofluorescence analysis

Immunofluorescence analysis for asexual stages, gametocytes, ookinetes, and sporozoites was carried out by fixing the cells with 4% paraformaldehyde and 0.0075% glutaraldehyde, followed by permeabilization with 0.1% Triton X-100 and subsequent treatment with 0.1 M glycine. Blocking was performed with PBS containing 2% BSA for 3 h. The incubation for primary antibodies was carried out in the same blocking buffer for 6 h, followed by the addition of secondary antibodies for 3 h^77^. For the oocyst, the mosquito gut was fixed with 4% paraformaldehyde and permeabilized with 0.1% Triton-X-100, followed by blocking in 4% BSA^78^. For exo-erythrocytic stages, HC-04 cells infected with sporozoites were fixed with 4% paraformaldehyde, permeabilized with 0.01% Triton X-100 and blocked with PBS containing 1% BSA^79^. Parasite GS-specific polyclonal sera were used at 1:250 dilution. Anti-UIS4 antibody (Origene, AB0042-200) was used at 1:1000 dilution. FITC-conjugated donkey anti-mouse IgG (Thermo Fisher Scientific, A24501) was used at 1:250 dilution. Rabbit anti-goat AF594 (Thermo Fisher Scientific, A-11080) was used at 1:400 dilution. Images were captured with 20x/60x/100x objectives using Olympus IX83 microscope with DP73 high-performance camera.

### Generation of *Pf*GS^cKS^ parasites and performing conditional knock sideways

For generating *Pf*GS^cKS^ parasites, C-terminal portion of *Pf*GS representing 1245–2069 bp (without stop codon at the end) was cloned into pSLI-2xFKBP-GFP plasmid for in-frame fusion with FKBP and GFP^37^. The following were the forward (F) and reverse (R) primers used: *Pf*GS^cKS^ (F): 5’-GCATGCGGCCGCTAAATATTCTATCATAATGATCCTTCTACTTTC-3’ and *Pf*GS^cKS^ (R): 5’-CGATCCTAGGACATTCATAATATAAGTGATAATCATAAGC-3’ (Sigma-Aldrich). The restriction sites are underlined. 50 μg of the plasmid construct was nucleofected into purified mature schizonts of *Pf*3D7 using P3 primary cell 4D-Nucleofector™ X Kit L (Lonza, V4XP-3024), and the parasites having episomal plasmid were selected after 24 h with 4 nM WR99210 (Jacobus Pharmaceutical Company Inc.). When the parasitemia reached around 2%, the selection of the integrated parasites was carried out by the addition of 400 μg G418 per ml of culture volume (Gibco, 1181031). The in-frame fusion of GS with FKBP and GFP in the selected parasites was confirmed by genomic DNA PCR, RT-PCR, Western and fluorescence analyses. To mislocalize *Pf*GS by conditional knock sideways, *Pf*GS^cKS^ parasites were nucleofected with 50 μg of mislocalizer pLyn-FRB-mCherry plasmid, followed by selection with 2 μg of Blasticidin-S (Thermo Fisher Scientific, R21001) per ml of culture volume. The presence of mislocalizer plasmid was confirmed by examining the mCherry fluorescence in live parasites and its membrane localization. The mislocalization of *Pf*GS was induced by the addition of 250 nM rapamycin (Sigma-Aldrich, R0395) and verified by the translocation of GFP fluorescence from the parasite cytosol to the plasma membrane in live parasites. For experiments involving gametocytes, *Pf*GS^cKS^ cultures were synchronized and gametocytes were induced by the crash method. When the culture parasitemia reached around 6%, 50 mM N-acetyl-D-glucosamine (Sigma-Aldrich, A3286) was added along with 250 nM rapamycin, and continued for four days after which, the addition of N-acetyl-D-glucosamine was stopped. The mislocalization of *Pf*GS in the gametocytes was also confirmed by fluorescence analysis and the formation of different stages of gametocytes was quantified by examining morphology and counting their numbers in Giemsa-stained smears.

### Generation of *Pf*GS^HA-DD^ parasites for conditional knockdown

For generating *Pf*GS^HA-DD^ parasites, C-terminal portion of *Pf*GS representing 909–2069 bp (without stop codon at the end) was amplified and cloned into pHADD plasmid^38^ for in-frame fusion with HA and destabilization domain (DD). The following forward and reverse primers were used: 5’- GCCACCGCGGGATGATAGTAAAAAAGTAAAGAAAAAATCCGG-3’ and 5’-GCCCGGTACCACATTCATAATATAAGTGATAATCATAAGCG-3’ (Sigma-Aldrich). The C-terminal portion of *Pf*GS with in-frame fusion of HA and DD was then amplified with the following forward and DD-specific reverse primers: 5’-GCAAGCGGCCGCGATGATAGTAAAAAAGTAAAGAAAAAATCCGG-3’ and 5’-GCAAGTCGACAGGTTCCGGTTTTAGAAGCTCCACA-3’ (Sigma-Aldrich). The restriction sites are underlined. The resultant fragment was cloned into pSLI-TGD plasmid replacing the GFP present in the plasmid. The plasmid was then nucleofected into purified mature schizonts of *Pf3*D7. Parasites having episomes were selected after 24 h with 4 nM WR99210 (Jacobus Pharmaceutical Company Inc.), followed by selection with G418 (400 μg/ml) for parasites having proper integration. The in-frame fusion of GS with HA and DD was confirmed by genomic DNA PCR, RT-PCR and Western analyzes. *Pf*GS^HA-DD^ cultures were continuously maintained in the presence of 0.5 μM Shield-1 since transfection (Takara Bio, 632189) and the extent of GS degradation was examined by the removal of Shield-1.

### Generation of *Pb*GSKO parasites

Deletion of GS in *Pb* ANKA parasites was carried out by double crossover recombination. In brief, PCR was carried out to amplify the 5’- and 3’-UTRs of GS from *Pb* genomic DNA. The following were the forward (F) and reverse (R) primers used: 5’-UTR (F): 5’-GCCAGGGCCCGCATGCTCCGAAATTGATACTCTGCATG-3’; 5’-UTR (R): 5’-GCCCAGATCTTTTGAAAAATAGGAAAAGTAAATAATATATGATGCG-3’; 3’-UTR (F): 5’-GCCAGGTACCCCAAAAGCACATACACTGTAATTTGCACTC-3’; and 3’-UTR (R): 5’-GCCCGCGGCCGCTATTCAAATGATTAAGAGGTGGCATATAATAAATG-3’ (Sigma-Aldrich). The resultant 5’- and 3’-UTR fragments were digested with *ApaI* and *BglII*, and *KpnI* and *NotI*, respectively, and cloned into *pL0006* plasmid. The plasmid construct was then digested with *ApaI* and *NotI*, followed by the purification of linear product containing hDHFR selection marker flanked on either side by 5’- and 3’-UTRs of *Pb*GS. The linear product was nucleofected into purified mature schizonts of *P. berghei* ANKA using P5 primary cell 4D-Nucleofector™ X Kit L. (Lonza, V4XP-5024). The nucleofected parasites were injected intravenously into 7-8 weeks old naïve BALB/c mice, followed by pyrimethamine selection (70 mg/L in drinking water). The selected parasites were then subjected to limiting dilution and *Pb*GSKO phenotype was verified with two independent clones. Targeted deletion of GS in *Pb*GSKO parasites was confirmed by genomic DNA PCR and RT-PCR using *Pb*GS-specific primers, and by Southern and Western analyzes. For Southern analysis, genomic DNA isolated from *Pb*WT and *Pb*GSKO parasites were digested with *EcoRI* and *AflII*, resolved on 0.7% agarose gel and transferred to nylon membrane. Digoxigenin (DIG) labeled probe for *Pb*GS 3’-UTR was prepared by PCR amplification with 3’-UTR forward and reverse primers using DIG DNA Labeling Kit (Roche, 11093657910). Hybridization of the DIG-labeled probe and its detection were carried out using DIG Luminescent Detection Kit (Roche, 11363514910) as per the manufacturer’s protocol.

### Sexual and liver stage development studies of *P. berghei*

To initiate the sexual stage development of *Pb* in *A. stephensi* mosquitoes, adult female mosquitoes of 5–7 days old were allowed to feed on mice infected with *Pb*WT or *Pb*GSKO parasites on day 8 post-infection when the blood parasitemia was around 5–10% with at least two exflagellation centers per field. The fully engorged mosquitoes were segregated and maintained at 19 °C and 75–80% humidity. At 20 h post-feeding, the blood bolus was collected from the infected mosquitoes to examine the ookinete formation and quantify the number of ookinetes. On day 10 post-feeding, the infected mosquito guts were dissected out and subjected to mercurochrome staining for determining the number of oocysts formed. On day 17 post-feeding, the salivary glands of the infected mosquitoes were dissected to examine the number of sporozoites present. In vivo liver stage development was assessed by injecting sporozoites intravenously into naïve Balb/c mice and examining for the appearance of blood-stage infections. For in vitro exflagellation analysis, 2.5 μl of tail vein blood was collected in the exflagellation medium of RPMI-1640 pH 8.0 containing 100 μM xanthurenic acid (Sigma-Aldrich, D120804) and 10% heat-inactivated FBS and incubated at 19 °C for ~12 min, followed by immediate counting of exflagellation centers by light microscopy. In vitro ookinete formation analysis was performed by examining the Giemsa-stained smears prepared from the blood that was collected in exflagellation medium and incubated at 19 °C for 22 h^8,73^. For in vitro exo-erythrocytic stage development studies, salivary glands were dissected under aseptic conditions and sporozoites were collected in DMEM/F-12 medium (Gibco, 12500039) containing 10% heat-inactivated FBS with penicillin-streptomycin (Gibco, 15140122). The isolated sporozoites were added to HC-04 cells (MRA-975) of 60–70% confluency, followed by the removal of culture supernatant after 3 h of sporozoite addition. The cells were then washed with medium and maintained at 37 °C for 52 h in a CO_2_ incubator to examine the formation of exo-erythrocytic stages^79^.

### MSO and PPT inhibition studies in *Pf* and *Pb*

In vitro inhibition, studies were carried out either in RPMI-1640 lacking L-glutamine (RPMI^-gln^) or RPMI-1640 medium containing physiological concentrations (0.5 mM) of L-glutamine (RPMI^Pgln^). MSO/PPT treatment was carried out in synchronized *Pf* cultures containing late rings and early trophozoites and the parasite growth was monitored for 24 h and 48 h by examining Giemsa-stained smears and by performing flow cytometry and ^3^H-hypoxanthine uptake. ^3^H-hypoxanthine was added to the cultures after 3 h of MSO/PPT addition. For in vitro *Pb* experiments, synchronized infections were initiated in naïve Balb/c mice and the infected blood was collected around 08:00 h in the morning when the parasites were predominantly in the early rings. After removing the plasma and buffy coat, the infected RBCs were washed either with glutamine-free or glutamine-containing RPMI-1640 with 10% FBS. The infected RBCs were resuspended in the respective RPMI-1640 medium to 5% hematocrit and MSO/PPT was added. ^3^H-hypoxanthine addition was carried out after 3 h and MSO/PPT treatment was performed for 18 h at 37 °C with intermittent mixing of the cultures at 1 h intervals. Since *Pb* has a 24 h life cycle in comparison with 48 h of *Pf* and the released *Pb* merozoites do not efficiently invade fresh RBCs in vitro, prolonged labeling studies could not be performed. Orotic acid (Sigma-Aldrich, O2750) and D-glucosamine hydrochloride (Sigma-Aldrich, G4875) supplementations for in vitro *Pf* cultures were carried out by adding them along with MSO/PPT. For in vivo treatment of *Pb*WT-infected mice with MSO/PPT, the inhibitors were dissolved in saline and injected intraperitoneally for four consecutive days at 10:00 h starting from day 4 post-infection. The parasite growth was examined by measuring the peripheral blood parasitemia in Giemsa-stained smears prepared from tail vein blood.

### Protein and RNA labeling

In vitro protein labeling studies for MSO/PPT treated *Pf* and *Pb* cultures were carried out by adding 100 μCi of ^35^S-Methionine and Cysteine mix (Invivo ProTwin Label (LCS-8), BRIT) to 4 ml culture volume at 3 h post-addition of MSO/PPT. After 9 h of radiolabelling, parasites were isolated by saponin lysis, resuspended in 50 mM Tris pH 8.0 containing 100 mM NaCl, 2% glycerol, and 0.1 % Triton X-100, and lysed by sonication. The lysates were resolved on 10% SDS-PAGE gel and phosphorimager scanning was carried out to examine the radiolabelling of proteins using Amersham Typhoon 5 Biomolecular Imager. In parallel, 10 μl of the lysates were spotted on Whatman filter paper grade I, dried and washed subsequently with hot and cold 10% (w/v) trichloroacetic acid to remove free amino acids and charged-tRNAs, followed by diethyl ether to remove lipids. The filter papers were then placed in Ultima Gold XR scintillation cocktail and the radioactive counts were measured using MicroBeta^2^ Microplate Counters. For in vitro RNA-labeling studies, *Pf* cultures were treated with MSO/PPT followed by the addition of 100 µCi ^32^P-orthophosphoric acid (LCP-32, BRIT) to 4 ml culture volume at 3 h post-addition of MSO/PPT. After 9 h of radiolabelling, parasites were isolated by saponin lysis, and total RNA was extracted using RNeasy Mini Kit (Qiagen, 74104) according to the manufacturer’s protocol. Total RNA was then resolved on 1.2% agarose gel and transferred to a nylon membrane. The membrane was dried and phosphorimager scanning was carried out using Amersham Typhoon 5 Biomolecular Imager. In parallel, 10 µl of the RNA was spotted on glass fibre filter and placed in an Ultima Gold XR scintillation cocktail, and the radioactive counts were measured using MicroBeta^2^ Microplate Counters. All these experiments were performed with identical parasitemia and hematocrit between the untreated and treated groups by equally splitting the infected cultures or infected-mouse blood.

### Estimation of aspartate, glutamate, asparagine, and glutamine levels in *Pf* and *Pb* parasites

The levels of aspartate, glutamate, asparagine, and glutamine in *Pf* and *Pb* pellets treated with MSO were estimated by HPLC as well as by LC-MS/MS. 10 ml of synchronized *Pf* cultures having ~2% parasitemia were treated with MSO for 12 h when the parasites were predominantly in late rings and early trophozoites, followed by saponin lysis to isolate the parasites. *Pb*WT and *Pb*GSKO parasites were also isolated by saponin lysis from infected mice when the blood parasitemia was around 5%. Parasite pellets were then extracted with 10 volumes of 50% methanol in water (v/v), followed by lyophilisation and solubilizing the lyophilized extracts with 50 μl of water^80^. For HPLC, the extracts were derivatized with OPA, separated on Poroshell 120 HPH-C18 column (4.6 mm × 100 mm X 2.7 μm) using Agilent 1260 Infinity HPLC System as mentioned for the recombinant protein assays. The peak areas of the MSO-treated samples with respect to the untreated controls or *Pb*GSKO samples with respect to *Pb*WT samples were used to measure the fold changes of aspartate, glutamate, asparagine, and glutamine. To compensate the variations that arise because of the changes in parasite yield, the data were normalized with the average fold changes of the peak areas obtained for at least five different amino acids - arginine, serine, histidine, threonine, and tyrosine that showed unambiguous separation in HPLC. For LC-MS/MS, the parasite extracts were derivatized with 6-aminoquinolyl-N-hydroxysuccinimidyl carbamate (AQC), cleaned up with SPE and dried under vacuum, followed by reconstitution with 50 μl of 0.5% acetonitrile containing 0.1% formic acid. 10 μl of the sample was used for LC-MS/MS analysis carried out in Acquity C18 column (1.8 μm, 2.1 mm × 100 mm) using Dionnex Ultimate3000 UHPLC system coupled with Q Exactive mass spectrometer (Thermo Fisher Scientific). Mobile phase consisted of Solvent A: 10 mM ammonium acetate containing 0.1% formic acid and solvent B: acetonitrile containing 0.1% formic acid. Mass spectrometry was performed under spray voltage of 4000/2500 V with vaporizer temperature of 250 °C, sheath gas, and auxillary gas flow rate of 30 and 10 Arb, respectively. The acquisition was performed in Parallel Reaction Monitoring mode at 35,000 resolution with a normalized collision energy of 25 eV. The calibration curves were prepared for the individual amino acids and the samples were spiked with internal standards containing deuterated amino acids. The fold changes of aspartate, glutamate, asparagine, and glutamine were calculated based on their relative abundances in MSO-treated and untreated controls or *Pb*GSKO and *Pb*WT samples, after normalizing with the average fold changes of the relative abundancies obtained for arginine, serine, histidine, threonine and tyrosine.

### eIF2α phosphorylation in *Pf* and *Pb* parasites

*Pf*3D7 cultures were subjected to two rounds of tight synchronization within a span of 3 h in the previous cycle, followed by one additional synchronization in the subsequent cycle immediately after RBC invasion. After 3 h post-invasion, ring stage parasites were treated for 6 h with 250 μM MSO in RPMI^-gln^ medium. For eIF2α phosphorylation analysis in *Pb*WT and GSKO parasites, infected mouse blood of similar parasitemia predominantly containing the ring stages was incubated in vitro with 250 µM MSO for 6 h in RPMI^-gln^ medium. The parasite pellets were then prepared by saponin treatment and lysed with 0.5x PBS containing 0.5% Triton X-100, Halt protease inhibitor and PhosSTOP (Roche, 4906845001). Western blot analysis was performed with phosphorylated eIF2α (#3398, Cell Signaling Technology) and total eIF2α (#9722, Cell Signaling Technology) antibodies.

### Proteomics analyzes

To examine the proteome of MSO-treated *Pf* parasites, proteins were extracted from the untreated and MSO-treated parasite pellets of two independent experiments. For each experiment, two different sets of untreated and MSO-treated cultures synchronized for late ring stages were treated with 50 μM MSO for 12 h. The untreated and MSO-treated parasite pellets isolated by saponin treatment were pooled separately during solubilization with 6 M urea in 25 mM ammonium bicarbonate buffer. The solubilized protein samples were then treated with DTT and iodoacetamide, and the urea concentration was decreased to 0.6 M by diluting with 25 mM ammonium bicarbonate. In-solution trypsin digestion was carried out overnight at 37 °C for 300 μg total protein using TPCK-treated trypsin (Sigma-Aldrich, 4352157). LC-MS/MS was performed with microflow reverse-phase LC in Eksigent Ekspert Nano LC 425 system (SCIEX) that was directly connected to a tandem quadrupole time-of-flight SCIEX TripleTOF 5600 + ESI-mass spectrometer. SCIEX Micro Trap Cartridge (Chrome XP; C18-CL, 5-μm, 120-Å pore size) was used to concentrate the samples. 0.1% (v/v) formic acid and 2% (v/v) acetonitrile in water was used to wash the trap cartridge, followed by the separation of the concentrated peptides using a SCIEX capillary reverse-phase column (ChromeXP, 3C18-CL-120, 3 μm, 120 Å and 0.3 × 150 mm). The flow rate was set at 5 μl/min and the run was carried out using the following solvents: solvent A − 98% water and 2% acetonitrile containing 0.1% formic acid (v/v), and solvent B − 98% acetonitrile and 2% water containing 0.1% formic acid (v/v). The gradient followed was 2 to 50% of solvent B for 28 min, followed by 50 to 90% of solvent B for 1 min, sustaining 90% of solvent B for 3 min, and then 90 to 5% of solvent B for 0.5 min with a final re-equilibration with 2% of solvent B for 2.5 min. Mass spectra and tandem mass spectra were recorded in positive-ion and high-sensitivity mode with a full scan resolution of 35,000 (full width at half maximum). The following parameters were used for ion source: IonSpray Voltage Floating (ISVF) = 5500; Ion Source Gas 1 (GS1) = 25; Ion Source Gas 1 (GS2) = 22; Curtain Gas Flow (CUR) = 30. The precursor ions were fragmented with nitrogen gas in a collision cell. 100 fmol beta-galactosidase digest (SCIEX) was used to calibrate TOF MS and TOF MS/MS spectra. The peptide spectra were recorded over a mass/charge (m/z) range of 350 to 1250, and MS/MS spectra were recorded over an m/z range of 150 to 1600 in data-dependent acquisition (DDA) mode. Analyst TF1.7.1. software was used for data acquisition and DDA was performed to obtain MS/MS spectra for the 15 most abundant parent ions following each survey MS1 scan (250-ms acquisition time per MS1 scan and 50-ms acquisition time per MS/MS). Dynamic exclusion features were set to an exclusion mass width of 50 mDa and an exclusion duration of 6 s. Paragon algorithm (ProteinPilot Software Version 5.0.2, SCIEX) was used to annotate MS/MS data against the reference proteomes of *Plasmodium falciparum* (UP000001450, Taxonomy: 36329; UP000030688, Taxonomy: 57266; UP000054282, Taxanomy: 57267; UP000054289, Taxonomy: 137071; UP000030673, Taxonomy: 5843; UP000019103, Taxonomy: 57270; UP000232684, Taxonomy: 5843) available at Uniprot (https://www.uniprot.org/) with the following parameters: TripleTOF 5600 instrument; alkylation of cysteines by iodoacetamide; trypsin enzyme digestion; ID Focus on biological modifications and the detected protein threshold [Conf] set at >10%. Peptides identified with ≥95 confidence were selected to calculate the fold change from the cumulative peptide intensities of the respective proteins. Cumulative peptide intensities of the identified proteins were normalized with respect to parasite actin I before determining Log_2_FC values. The mass spectrometry proteomics data of LC-MS/MS have been deposited to the ProteomeXchange Consortium via the PRIDE partner repository with the dataset identifier PXD032797. Functional classification of the proteins was performed based on gene ontologies available at PlasmoDB.

### Nucleotide analyzes

ATP, GTP, CTP, UTP, AMP, GMP, CMP, and UMP levels of untreated control and MSO-treated *Pf*3D7 parasites were assessed from three independent experiments. For this, 20 ml of synchronized *Pf* cultures having ~3% parasitemia were treated with 50 μM MSO for 12 h when the parasites were predominantly in the late ring stages. Parasites were isolated by saponin lysis and nucleotides were extracted by adding 10 volumes of 0.5 M perchloric acid to the parasite pellets, followed by vortexing and incubating on ice for 20 min. The extracts were neutralized with 2.5 M potassium hydroxide and incubated for 20 min in ice. The extracts were then centrifuged at 16,000 *g* for 15 min at 4 °C and the supernatants were filtered through Amicon Ultra (0.5 mL-10 kDa) centrifugal filter at 16,000 *g* for 15 min at 4 °C^81,82^. The filtrate (~100 μl) was immediately frozen in liquid nitrogen and stored at −80 °C. LC-MS analyzes were performed by injecting 10 µL of the sample into a Shimadzu Prominence-I HPLC (Shimadzu Corporation, Kyoto, Japan) interfaced with Shimadzu triple quadrupole LCMS-8045 mass spectrometer (Shimadzu Corp.). For LC separation, samples were run on Shim-pack GIST column (C18, 75 mm × 4.6 mm, 5 μm) at a flow rate of 0.8 mL/min with slight modifications^83^. The solvents used were 0.1 % formic acid in water (A) and 100% acetonitrile (B) with a 100–80% gradient of A from 0–8 min, followed by 80–20% from 8–15 min, 20% from 15–18 min, 20–80% from 18–22 min, 80–100% from 22–24 min, and 100% from 24–26 min. The electrospray ionization (ESI) was performed in both positive and negative ion modes using following parameters: nebulizing gas flow = 3 L/min, heating gas = 10 L/min, interface temperature = 300 °C, drying gas flow = 10 L /min and DL temperature = 250 °C. Full-scan mass spectra were acquired over a mass range of m/z 50–1900. Processing, visualization, and analysis of mass spectrometry data were carried out using MZmine Version 2.53. Mass detection noise level was set to 1.0E1 in centroid mode and peak lists were generated with a minimum time span of 0.05 min and m/z tolerance of 0.01 m/z. Chromatogram deconvolution was achieved with a minimum peak height of 1.0E1, a peak duration range of 0.02 − 10 min, and a baseline level of 1.0E1. The duplicate peaks were filtered with m/z tolerance of 0.1 m/z (or 5 ppm) and a retention time tolerance of 2.0 min for the peak list. KEGG compound database (https://www.genome.jp/kegg/compound/) was used for metabolite identification of the individual peaks with m/z tolerance 0.1–0.3.

### Inhibition studies with *Pf* and *Pv* clinical samples

The collection of clinical samples was carried out with the approval of the Institutional Ethics Committee (IEB)/ Institutional Review Board (IRB) (94/HEC/19; IEC:248/2019) from febrile patients who visited Ispat General Hospital, Rourkela, India, and KMC Hospital, Mangalore, India, after obtaining their informed consent. *Pf* and *Pv* infections were confirmed by examining Giemsa-stained thick and thin blood smears under the light microscope, and by performing rapid diagnostic test and PCR for *18* *S rRNA*. The infected blood was collected in heparinized vacutainers and the subsequent procedures were carried out in BSL-2 facility under sterile conditions. The infected blood was centrifuged to remove the plasma and buffy coat, followed by two washes with RPMI-1640 medium containing 10% heat-inactivated human O^+^ serum. The packed cells were then resuspended in the same medium and the hematocrit was adjusted to 5%. The cultures were treated with different concentrations of MSO/PPT and ^3^H-hypoxanthine addition was carried out after 3 h of MSO/PPT addition to assess the parasite growth. The cultures were incubated following the standard conditions of *Pf* maintenance and the experiments were performed for each clinical isolate in RPMI^-gln^ as well as in RPMI^Pgln^. All these experiments were typically carried out in a final culture volume of 1.2 ml for the respective concentrations of MSO or PPT. Parasite growth assessment was also verified by examining the Giemsa-stained smears.

### Ring-stage survival assays with ART-resistant *Pf*Cam3.I^R539T^ strain

*Pf*Cam3.I^R539T^ strain was maintained under standard conditions. When the parasitemia reached around 2% with predominant ring stages, the cultures were subjected to two rounds of tight synchronization within a span of 3 h in the previous cycle, followed by one additional synchronization in the subsequent cycle when they are in early rings. The parasitemia was adjusted to 0.5–1.0% in 2% hematocrit and ring-stage survival assays were carried out by the addition of 700 nM ART (Sigma-Aldrich, 361593) or DHA (Sigma-Aldrich, D7439)^84^. For the combination of ART with MSO/PPT and DHA with MSO/PPT, MSO or PPT was added along with ART/DHA. The exposure of rings to ART/DHA or ART/DHA in combination with MSO/PPT was precisely carried out for 6 h, followed by the removal of supernatant and washing the infected-RBCs twice with RPMI-1640 medium. The infected RBCs were then resuspended in RPMI-1640 medium and maintained continuously. The medium change was carried out at 48 and 72 h, and after 96 h, the culture parasitemia was examined by Giemsa-stained smears and flow cytometry. For growth assessment by ^3^H-hypoxanthine uptake, the label was added at 72 h and the cells were harvested at 96 h. The experiments were carried out independently in RPMI^-gln^ and RPMI^Pgln^ medium.

### Other procedures

Western blot analyzes were carried out following the standard protocols and the blots were developed either with Pierce ECL Western blotting substrate (Thermo Fischer Scientific, 32209) for horse radish peroxidase or with 5-bromo-4-chloro-3-indolyl phosphate (MP Biomedicals, 215004283) and P-nitro blue tetrazolium chloride (MP Biomedicals, 210041690) for alkaline phosphatase. Rabbit polyclonal anti-GFP antibodies (Abcam, ab290) was used at 1:5000 dilution. The secondary antibodies - goat anti-mouse IgG H&L (HRP) (Abcam, ab97023) and goat anti-rabbit IgG H&L (HRP) (Abcam, ab97051) were used at 1:10,000 dilution, and goat anti-mouse IgG H&L (AP) (Abcam, ab97051) was used at 1:5,000 dilution. Subcellular fractionation of cytosol and membrane fraction was carried out by resuspending the *Pf*GS^cKS+Lyn^ parasite pellet in 150 µl of 50 mM Tris pH 7.5 containing protease inhibitors, followed by incubation in ice for 20 min and centrifugation at 20,000 *g*. The supernatant (cytosol) was separated and the pellet (membrane fraction) was resuspended in the same buffer. Plasma samples from *Pb*-infected mice were collected at similar parasitemia on day 8 post-infection when the parasites were predominantly in trophozoite stages using heparin as an anticoagulant. Plasma ammonia levels were measured using an ammonia assay kit (Abcam, ab83360). Plasma ATP levels were quantified using Luminescent ATP detection kit (Abcam, ab113849). Glutathione assays with parasite pellets were carried out using Glutathione colorimetric detection kit (Thermo fisher Scientific, EIAGSHC). Polyclonal sera against r*Pf* and r*Pb* GS were raised using Freund’s complete (Sigma-Aldrich, F5881) and incomplete (Sigma-Aldrich, F5506) adjuvants.

### Statistical analyzes

GraphPad Prism Version 7.00 software was used to plot the graphs. Statistical analyzes were carried out using unpaired Welch’s t-test (two-sided), two-way ANOVA, and log-rank (Mantel-Cox) test. Tukey test was performed for two-way ANOVA and multiple comparisons were corrected by statistical hypothesis testing. n.s - not significant, *P < 0.05, **P < 0.01, ***P < 0.001. The non-linear regression fit for the inhibitor versus response curve was performed and R-squared values were calculated using GraphPad Prism 7.00.

### Reporting summary

Further information on research design is available in the Nature Portfolio Reporting Summary linked to this article.

## Supplementary information

Supplementary Information

Description of Additional Supplementary Files

Supplementray Data 1

Supplementary Data 2

Reporting Summary

## Data Availability

Source data are provided with this paper. The raw files and extracted chromatograms of nucleotide mass spectrometry analyzes are available in figshare^85^. 10.6084/m9.figshare.23553822.v1. Reference proteomes of *Plasmodium falciparum* (UP000001450, Taxonomy: 36329; UP000030688, Taxonomy: 57266; UP000054282, Taxanomy: 57267; UP000054289, Taxonomy: 137071; UP000030673, Taxonomy: 5843; UP000019103, Taxonomy: 57270; UP000232684, Taxonomy: 5843) available at Uniprot (https://www.uniprot.org/) were used for proteomics analyzes. Proteomics data have been deposited to the ProteomeXchange Consortium via the PRIDE partner repository with the dataset identifier PXD032797. Structures of *Pf* (PDB ID: 6PEW), *St* (PDB ID: 1F1H), *Mt* (PDB ID: 2WGS) and *Hp* GS (PDB ID: 5ZLP) were accessed from RCSB Protein Data Bank (https://www.rcsb.org/). Source data are provided with this paper.

## Source data

Source Data
