# Supplementary material for: Distinct evolution of type I glutamine synthetase in Plasmodium and its species-specific requirement
Source: Nat Commun. 2023 Jul 14;14:4216. doi: 10.1038/s41467-023-39670-4 (PMC10349072; doi:10.1038/s41467-023-39670-4)
Supplement: Supplementary file 1 — Supplementary Information [file 41467_2023_39670_MOESM1_ESM.pdf]

Supplementary Information

Distinct Evolution of Type I Glutamine Synthetase in *Plasmodium* and its Species-specific Requirement

Sourav Ghosh, Rajib Kundu, Manjunatha Chandana, Rahul Das, Aditya Anand, Subhashree Beura, Ruchir Chandrakant Bobde, Vishal Jain, Sowmya Ramakant Prabhu, Prativa Kumari Behera, Akshaya Kumar Mohanty, Mahabala Chakrapani, Kapaettu Satyamoorthy, Amol Ratnakar Suryawanshi, Anshuman Dixit, Govindarajan Padmanaban and Viswanathan Arun Nagaraj

Supplementary Discussion

Supplementary Figures

Supplementary References

Supplementary Discussion

Sequence comparison of *Plasmodium* GS with GS of other organisms

Glutamine synthetase (GS) has evolved as three distinct types - I, II and III. GS I and GS III enzymes exist as dodecamers and are predominantly present in bacteria and archaea. GS II enzymes are decamers that are commonly present in eukaryotes including humans and plants. The existence of more than one type of GS and multiple isoforms of a particular type in different organisms is also known (Supplementary References 1-4). The sequence length of GSI is close to 470 amino acids whereas, GS II and III are around 370 and 700 amino acids, respectively (Supplementary Reference 5). GS I can be classified into Iα and Iβ based on the regulatory mechanisms. GS Iβ has a characteristic ~25 amino acid insertion representing 146-170 residues in *St*GS (149-176 residues in *Mt*GS and 155-180 residues in *Hp*GS) and an adenylylation site with a conserved tyrosine residue (NLY^{398}DLP in case of *St*GS and DLY^{406}ELP in case of *Mt*GS) (Fig. 1a) that are absent in type Iα GS (Supplementary Fig. 1a). Despite having a 25-amino acid insertion, *Hp*GS lacks the tyrosine residue that undergoes adenylylation (Supplementary Reference 3) and is replaced with phenylalanine (NLF^{407}KLT) (Fig. 1a). Although *Plasmodium* GS shows ~50% similarity and ~30% identity with bacterial type I GS, it can neither be classified as Iα nor Iβ. Further, bottom-up structural proteomics performed with near-atomic-resolution cryo-electron microscopy (cryoEM) for the sucrose gradient fraction of *Pf* lysates with an *ab initio* cryoID program has identified parasite GS as a type I enzyme with a classical dodecameric structure (Supplementary Reference 6).

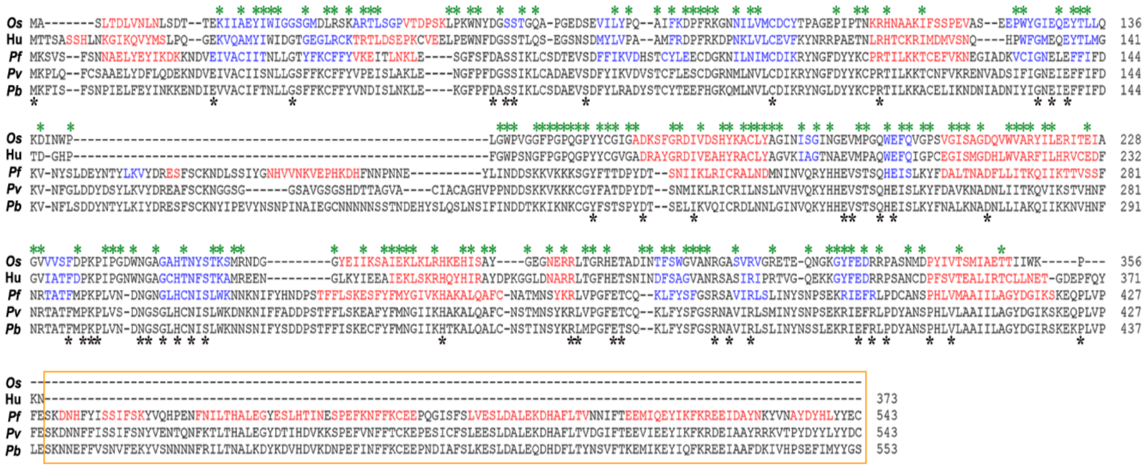

Multiple sequence alignment of *Oryza sativa* (*Os*) and human (Hu) type II GS with *Plasmodia* GS. The absence of long C-terminal extension in type II GS is highlighted in yellow box. Identical residues across the species are highlighted with black asterisks. Identical residues within type II GS are highlighted with green asterisks. The sequence alignment was performed with Clustal Omega (https://www.ebi.ac.uk/Tools/msa/clustalo/).

The alignment of parasite GS with GS II of human and *Oriza sativa* shows around 28% similarity and 16% identity. The presence of the long C-terminal extension protruding from each subunit and entering the second hexamer (Supplementary Reference 7) that is characteristic of GS I dodecamers including *Plasmodia* GS is absent in GS II. Similar alignment with GS III of *Ruminococcus albus* and *Bacteroides fragilis* shows around 25% similarity and 14% identity, and the signature motifs reported for GS III (Supplementary Reference 8) are absent in *Plasmodia* GS. Thus, parasite GS belongs to a unique type I enzyme of prokaryotic origin.

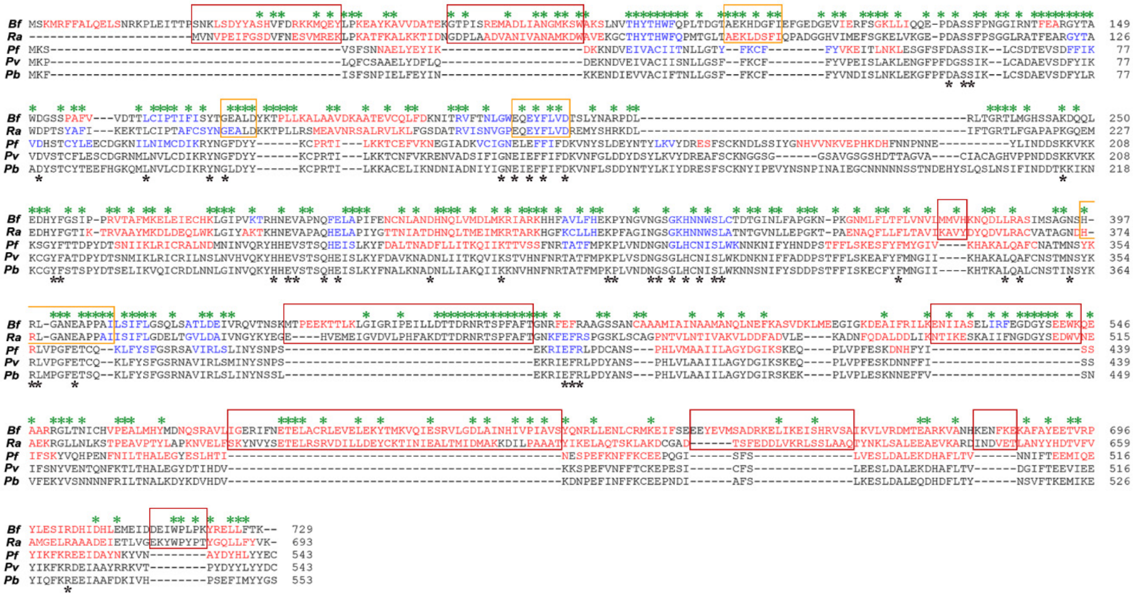

Multiple sequence alignment of *Bacteroides fragilis* (*Bf*) and *Ruminococcus albus* (*Ra*) type III GS with *Plasmodia* GS. The signature motifs and the additional stretches present in type III GS are highlighted in yellow and brown boxes, respectively. Identical residues across the species are highlighted with black asterisks. Identical residues within type III GS are highlighted with green asterisks. The sequence alignment was performed with Clustal Omega (https://www.ebi.ac.uk/Tools/msa/clustalo/).

Conditional knockdown of *Pf*GS

In addition to conditional knock sideways, we attempted for conditional knockdown of *Pf*GS by two independent approaches: fusing HA-tagged destabilization domain (DD) at the N-/C-terminus of *Pf*GS for Shield-1-based proteosomal degradation and introducing glmS ribozyme in the 3'-UTR of *Pf*GS for glucosamine inducible ribozyme activation (Supplementary Reference 9). We could only succeed in generating the transgenic parasites (*Pf*GS$^{HA\text{-}DD}$) having HA-tagged destabilization domain (DD) at the C-terminus of *Pf*GS (Supplementary Figure 5a).

The integration was confirmed by performing PCR analyses for the genomic DNA and RNA isolated from *Pf*GS$^{HA\text{-}DD}$ parasites (Supplementary Figure 5b,c). Western analysis carried out with GS antibody showed the expression of 81 kDa fusion protein in *Pf*GS$^{HA\text{-}DD}$ parasites (Supplementary Figure 5d). As observed for *Pf*GS^{cKS} parasites, *Pf*GS$^{HA\text{-}DD}$ parasites displayed a compromised growth in RPMI^{Pgln} medium. With respect to the parental *Pf*3D7 strain, there was almost 50% reduction in parasite growth (Supplementary Figure 5e). Further, *Pf*GS$^{HA\text{-}DD}$ parasites failed to grow in RPMI^{-gln} medium and no viable parasites could be detected after 24 h of glutamine removal. To rule out the emergence of any viable parasite at a later point of time, *Pf*GS$^{HA\text{-}DD}$ parasite cultures maintained continuously in RPMI^{-gln} medium were examined for three weeks. These results once again confirmed that the intervention of endogenous *Pf*GS activity affects parasite growth, and Hb-derived and/or extracellular glutamine are inadequate for the optimal growth of *Pf*. However, *Pf*GS$^{HA\text{-}DD}$ transgenic parasites did not support Shield-1-based GS knockdown and there was no significant decrease in the GS-HA-DD levels after removing Shield-1. In agreement, the growth of *Pf*GS$^{HA\text{-}DD}$ parasites did not change significantly after the removal of Shield-1. This was verified for a prolonged duration of Shield-1 removal and normalizing the decrease in the parental *Pf*3D7 parasite growth that happens due to Shield-1 addition. We feel that the complex dodecameric structure of *Pf*GS and its abundant expression in the asexual stages probably limit the exposure of DD for proteosomal degradation and Shield-1 binding. In case of conditional knock sideways, the high expression of plasma membrane-targeting Lyn-FRB-mCherry driven by episomal plasmid could have helped to successfully mislocalize *Pf*GS. Since the bifunnel active site is formed by the adjacent monomers of upper and lower hexamers, Lyn-targeting to the plasma membrane through FKBP-FRB interaction between GS-FKBP-GFP and Lyn-FRB-mCherry might have disrupted the oligomer formation resulting in the loss of GS activity.

Supplementary Figures

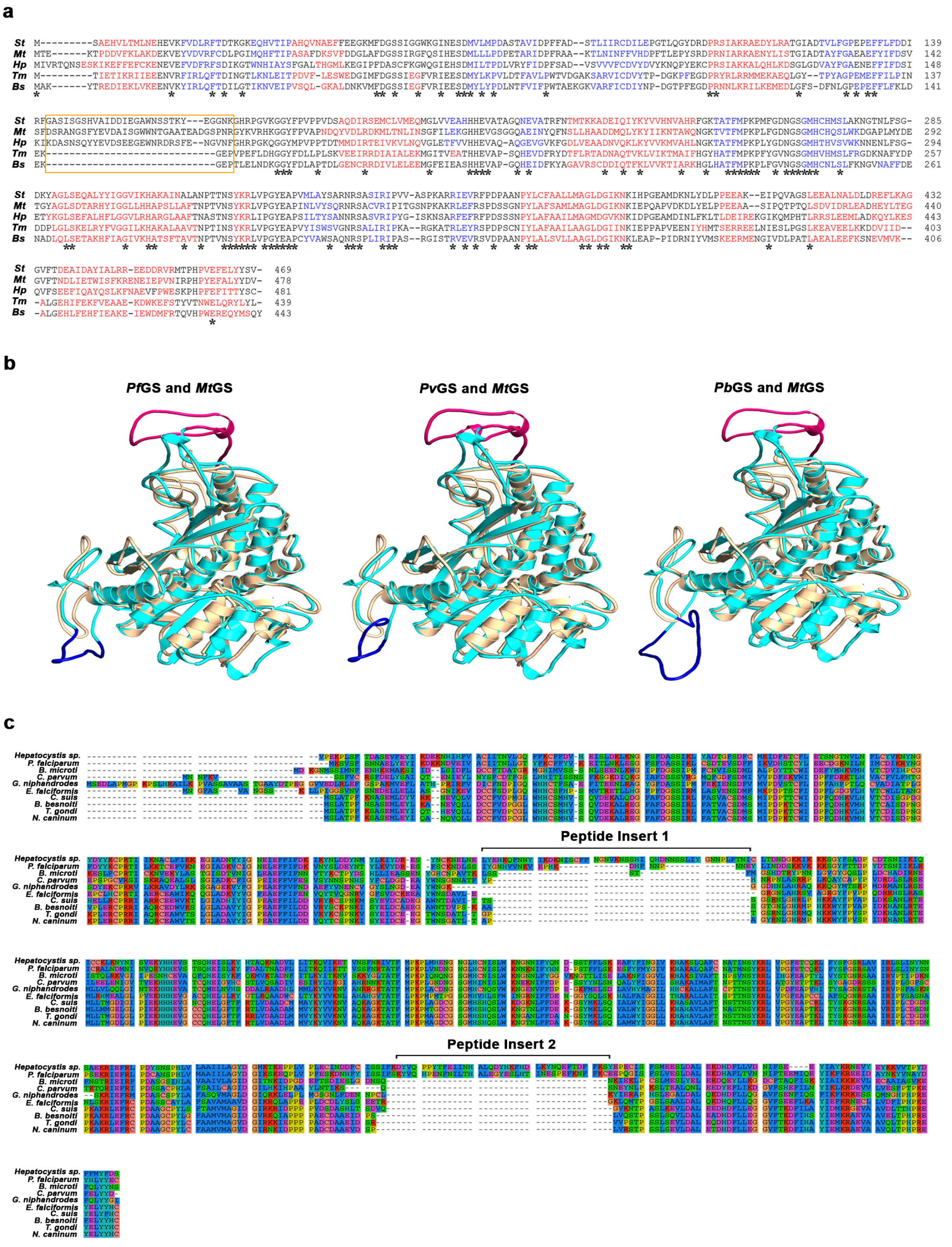

Supplementary Figure 1: Sequence and structural analysis of *Plasmodia* GS. a, Alignment of GS Iα (*Thermotoga maritima* (*Tm*) and *Bacillus subtilis* (*Bs*)) with GS Iβ (*St*, *Mt* and *Hp*). The absence of ~25 amino acid insertion in type Iα GS is highlighted in yellow box. Identical residues across the species are highlighted with black asterisks. The sequence alignments were

performed with Clustal Omega (https://www.ebi.ac.uk/Tools/msa/clustalo/). **b,** Superimposition of *Pf*, *Pv* and *Pb* GS monomers with *Mt*GS monomer. *Plasmodia* GS are shown in cyan and *Mt*GS is shown in wheat colors. *Plasmodia* GS-specific peptide inserts are represented in blue (Insert 1) and red (Insert 2). **c,** Sequence comparison of GS from the phylum Apicomplexa. GS sequences of *Hepatocystis Sp.*(HEP_00472600; PlasmoDB), *Babesia microti* (BMR1_03g00626; PiroplasmaDB), *Cryptosporidium parvum* (cgd6_4570; CryptoDB), *Gregarina niphandrodes* (GNI_119090; CryptoDB), *Eimeria falciformis* (EfaB_MINUS_15576.g1336; ToxoDB), *Cystoisospora suis* (CSUI_003150; ToxoDB), *Besnoitia besnoti* (BESB_007290; ToxoDB), *Toxoplasma gondi* (TGGT1_273490; ToxoDB) and *Neospora caninum* (Ncaninum_LIV_000051800; ToxoDB) are aligned with *Pf*GS using SeaView Version 3.2 (http://pbil.univ-lyon1.fr/software/seaview3). The peptide inserts 1 and 2 are absent in the GS of other apicomplexan parasites except for *Hepatocystis sp*. that has similar peptide inserts like *Plasmodia*.

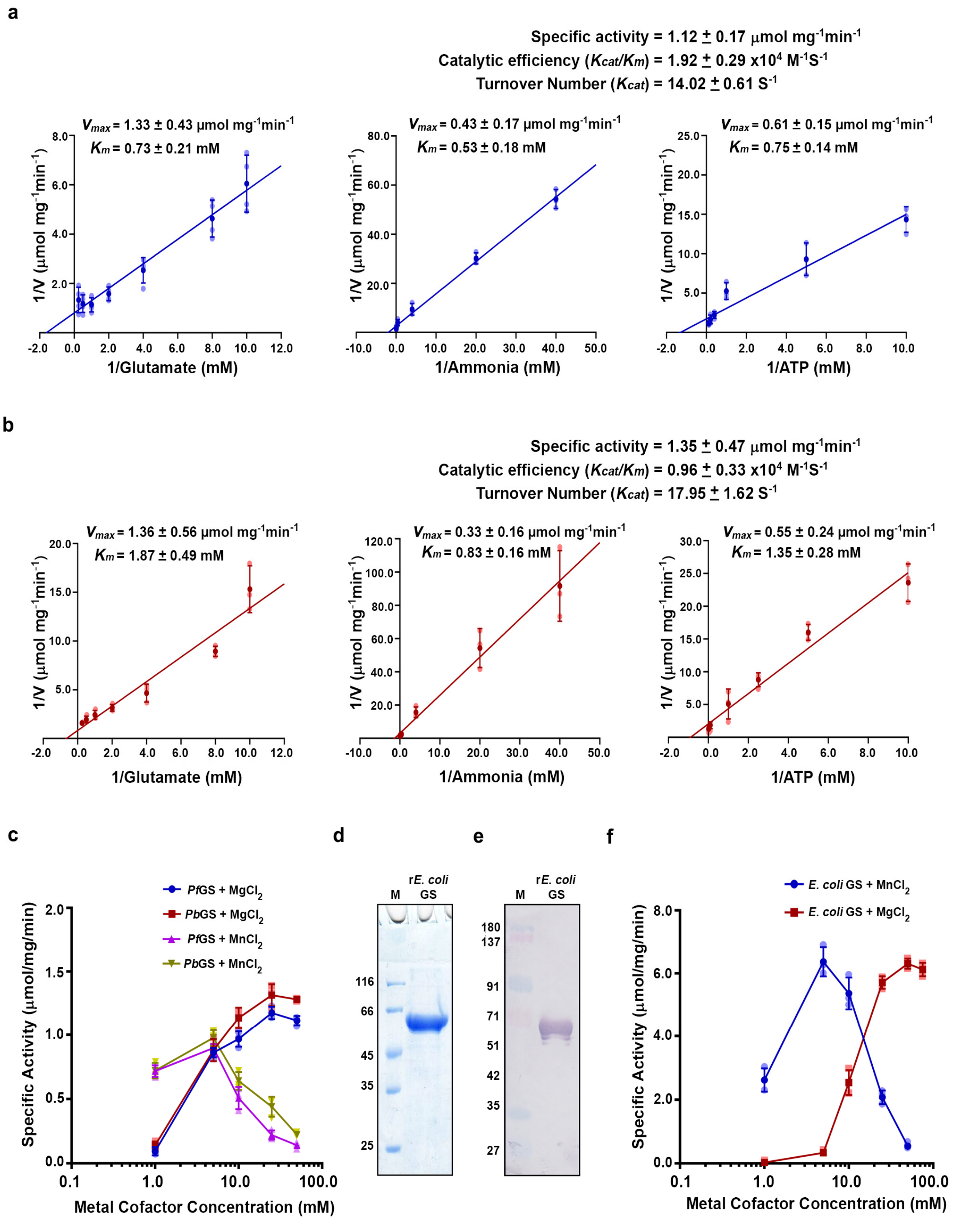

Supplementary Figure 2: Kinetic characterization and metal ion cofactor requirement of r*Pf*GS and r*Pb*GS. a, Lineweaver-Burk plots showing V_{max} and K_m values of r*Pf*GS for glutamate, ammonia and ATP. **b,** Lineweaver-Burk plots showing V_{max} and K_m values of r*Pb*GS for glutamate, ammonia and ATP. V_{max} and K_m values (mean ± SD) were derived from Lineweaver-Burk plots and represent at least three different protein preparations. r*Pf*GS and r*Pb*GS showed K_m values of 0.73 ± 0.21 and 1.87 ± 0.49 mM for glutamate, 0.53 ± 0.18 and 0.83 ± 0.16 mM for ammonia and 0.75 ± 0.14 and 1.35 ± 0.28 mM for ATP, respectively. The K_m values of glutamate for parasite GS are comparable with the values reported for *St* (~2.1 mM), *Mt* (~3 mM), *E. coli* (~5.5 mM), *Arabidopsis* (~0.67 mM) and human GS (~3.5 mM).

This is also the case for the K_m values of ammonia and ATP (Supplementary References 10-14). The specific activities of r*Pf*GS and r*Pb*GS were determined at saturating concentrations of the substrates. The K_{cat}/K_m values of parasite GS are comparable with the values reported for *E. coli* type I GS (2.8 x 10^4 $M^{-1}S^{-1}$) and *Arabidopsis* type II GS (6.5 x 10^3 $M^{-1}S^{-1}$) (Supplementary References 13,15). **c,** Specific activities of r*Pf*GS and r*Pb*GS obtained with different concentrations of $MgCl_2$ and $MnCl_2$. The data represent mean $\pm$ SD from three different experiments. **d,** Coomassie gel picture of purified r*E.coli* GS. Lane M: Protein molecular weight marker (kDa). **e,** Western analysis of purified r*E.coli* GS with his-tag antibody. Lane M: Protein molecular weight marker (kDa). For **d** and **e**, n = 3 independent experiments. **f,** Specific activity of r*E.coli* GS obtained with different concentrations of $MgCl_2$ and $MnCl_2$. The data represent mean $\pm$ SD from three different experiments. For (**a**,**b**,**c**,**f**), individual data points are shown with the respective light shaded colors. Source data are provided as a Source Data file.

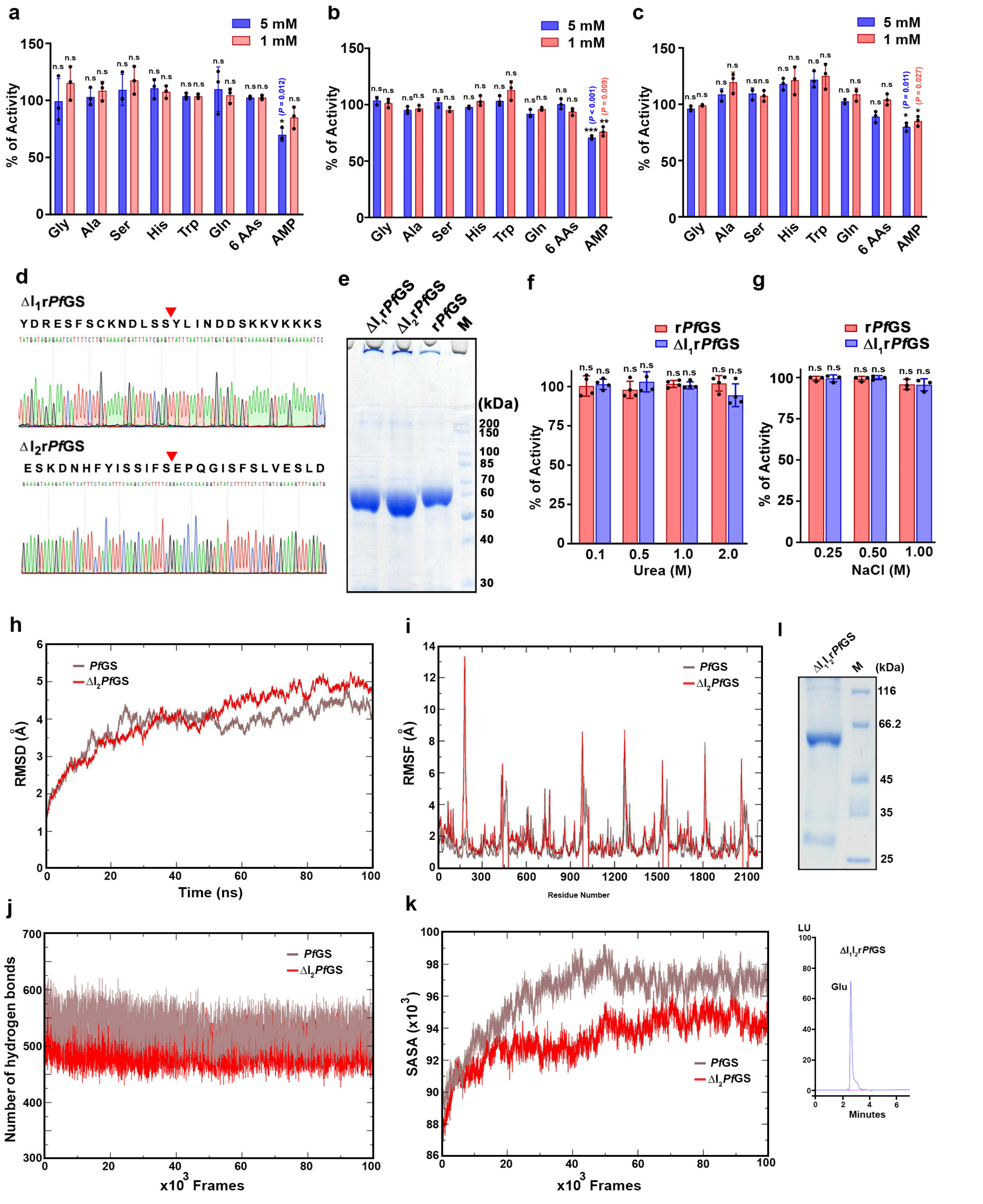

Supplementary Figure 3: Feedback inhibition of *Plasmodia* GS, effect of urea and salt on ΔI_1r*Pf*GS activity, MD simulations of ΔI_2*Pf*GS, and $\Delta I_1 I_2$r*Pf*GS activity. a, Feedback inhibition of r*Pf*GS in the presence of $MnCl_2$ at 1 mM and 5 mM concentrations of amino acids and AMP. **b,c,** Feedback inhibition of r*Pb*GS at 1 mM and 5 mM concentrations of amino acids and AMP in the presence of $MgCl_2$ and $MnCl_2$, respectively. "6AAs" represents mixture of all the six amino acids. For "6AAs" of 5 mM concentration, tryptophan alone was used at 2.5 mM concentration because of its limited solubility and the rest were used at 5 mM concentration. For **a-c**, percentage of activities (mean ± SD) with respect to control (without feedback inhibitor) are shown. n = 3 independent protein preparations. (n.s - not significant, $^*P<0.05$,

$P<0.01$, unpaired t-test; two-sided). **d, DNA sequencing confirmation for ΔI_1r*Pf*GS and ΔI_2r*Pf*GS plasmids. Amino acids corresponding to the DNA sequence are shown and inverted red triangles indicate the deleted portions. **e,** Coomassie gel pictures of r*Pf*GS, ΔI_1r*Pf*GS and ΔI_2r*Pf*GS expressed and purified from *E. coli*. Lane M: Protein molecular weight marker (kDa). n = 3 independent experiments. **f,g,** Effect of urea and NaCl on ΔI_1r*Pf*GS activity, respectively. Percentage of activity (mean $\pm$ SD) with respect to control (without urea or NaCl) is shown for the respective enzymes. n = at least 3 independent protein preparations (n.s - not significant, unpaired t-test; two-sided). **h,** C_α backbone RMSD values of *Pf*GS and ΔI_2*Pf*GS with respect to their starting structures in MD simulations performed for 100 ns. **i,** Residue-wise comparison of RMSF for *Pf*GS and ΔI_2*Pf*GS. **j,** Number of hydrogen bonds in MD simulations of *Pf*GS and ΔI_2*Pf*GS. **k,** SASA of *Pf*GS and ΔI_2*Pf*GS in MD simulations. For **h**-**k**, MD simulations were performed with four subunits - two each from upper and lower hexamers. **l,** Coomassie gel picture of $\Delta I_1 I_2$r*Pf*GS expressed in *E. coli* and purified using Ni^{2+}-NTA resin. Lane M: Protein molecular weight marker (kDa). n = 3 independent experiments. HPLC chromatogram of enzyme assay performed with $\Delta I_1 I_2$r*Pf*GS is shown below. Lack of enzyme activity in $\Delta I_1 I_2$r*Pf*GS was also verified with $MnCl_2$ and for a prolonged duration of 6 h. n = 3 independent protein preparations. Source data are provided as a Source Data file.

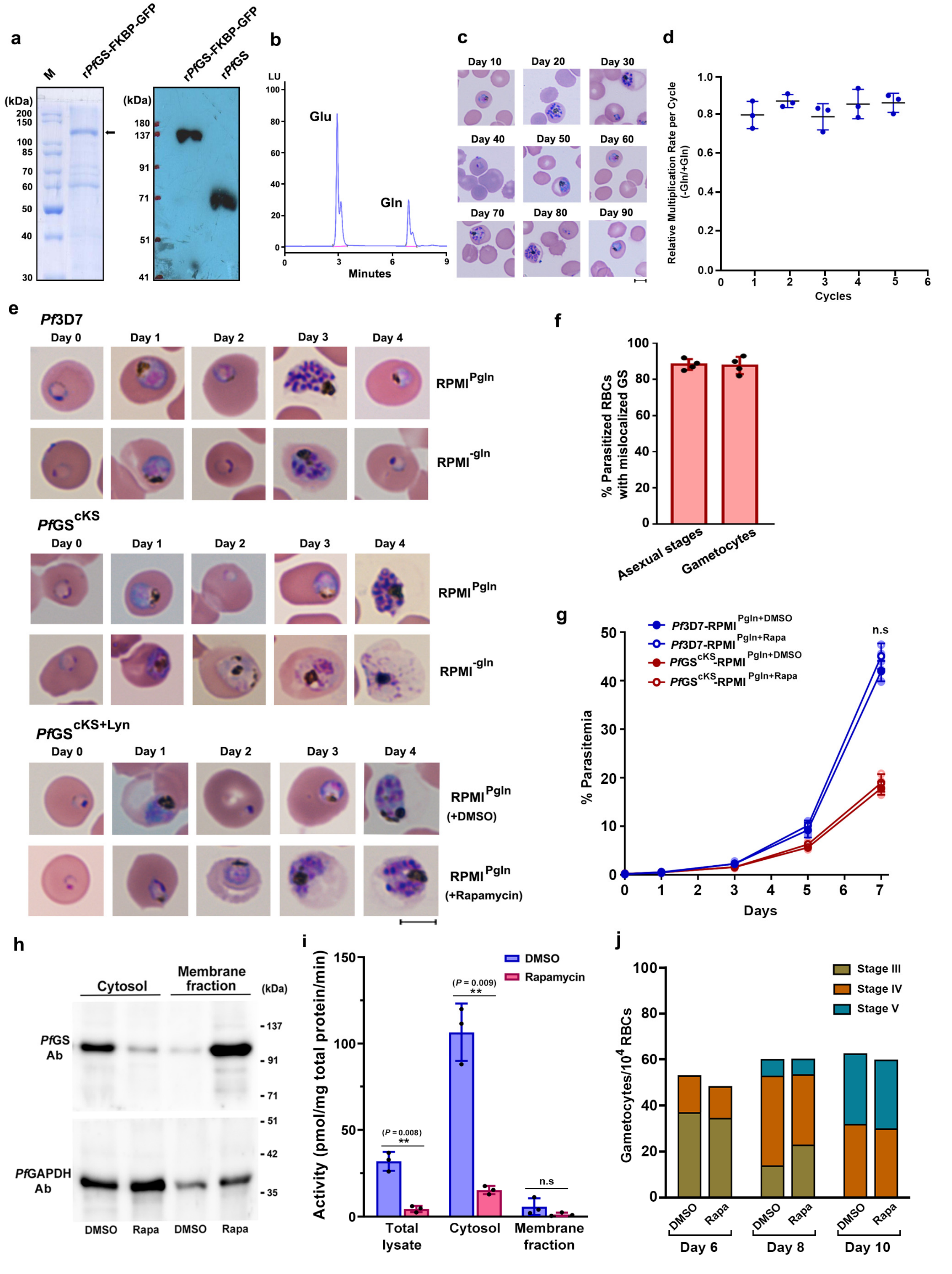

Supplementary Figure 4: Purification of r*Pf*GS-FKBP-GFP and growth analysis of *Pf*3D7, *Pf*GS^{cKS} and *Pf*GS$^{cKS+Lyn}$ parasites. a, Coomassie gel picture (left) of r*Pf*GS-FKBP-GFP expressed in *E. coli* and purified using Ni^{2+}-NTA resin. Lane M: Protein molecular weight marker (kDa). Black arrow represents the purified r*Pf*GS-FKBP-GFP (~120 kDa). Since there were a few additional protein contaminants, 60% of the total protein estimated for the purified fraction was used to calculate the specific activity in the enzyme assays. Western analysis

(right) of r*Pf*GS-FKBP-GFP with anti-his tag antibodies. r*Pf*GS was used as control. n = 2 independent experiments. **b,** HPLC chromatogram of r*Pf*GS-FKBP-GFP enzyme assay showing the less formation of glutamine. **c,** Giemsa-stained images of *Pf*3D7 parasites maintained continuously in $RPMI^{-gln}$ medium. Images were captured using 100x objective. Scale bar = 5 μM. **d,** *In vitro* growth assessment of *Pf*3D7 parasites in $RPMI^{-gln}$ medium. The multiplication rate for each cycle was calculated based on the parasitemia determined by FACS for that particular cycle with respect to the previous cycle. Relative multiplication rates (mean ± SD) for the respective cycles were determined by dividing the -Gln multiplication rate by +Gln multiplication rate. n = 3 different experiments. **e,** Representative Giemsa-stained images of *Pf*3D7, *Pf*GS^{cKS} and *Pf*$GS^{cKS+Lyn}$ parasites on various days. Images were captured using 100x objective. Scale bar = 5 μM. n = 3 independent experiments. **f,** Percentage of parasitized RBCs (mean ± SD) showing mislocalization of GS in *Pf*$GS^{cKS+Lyn+Rapa}$ cultures. Parasitized RBCs from 100 different fields were examined using 100x objective for GS mislocalization. n = 4 independent experiments. **g,** Effect of rapamycin on asexual stage growth of *Pf*3D7 and *Pf*GS^{cKS} parasites in $RPMI^{Pgln}$ medium. (mean ± SD; n.s - not significant, Two-way ANOVA). n = 3 independent experiments. **h,** Western analysis of GS mislocalization in *Pf*$GS^{cKS+Lyn}$ parasites after 48 h of rapamycin treatment. n = 2 independent experiments. **i,** GS activity in the total lysate, cytosol and membrane fraction of *Pf*$GS^{cKS+Lyn}$ parasites. n = 3 independent experiments. The data represent mean ± SD. **j,** Effect of rapamycin on gametocyte maturation of *Pf*3D7 parasites in $RPMI^{Pgln}$ medium. n = 2 independent experiments. The data represent mean values. Rapa - rapamycin. Source data are provided as a Source Data file.

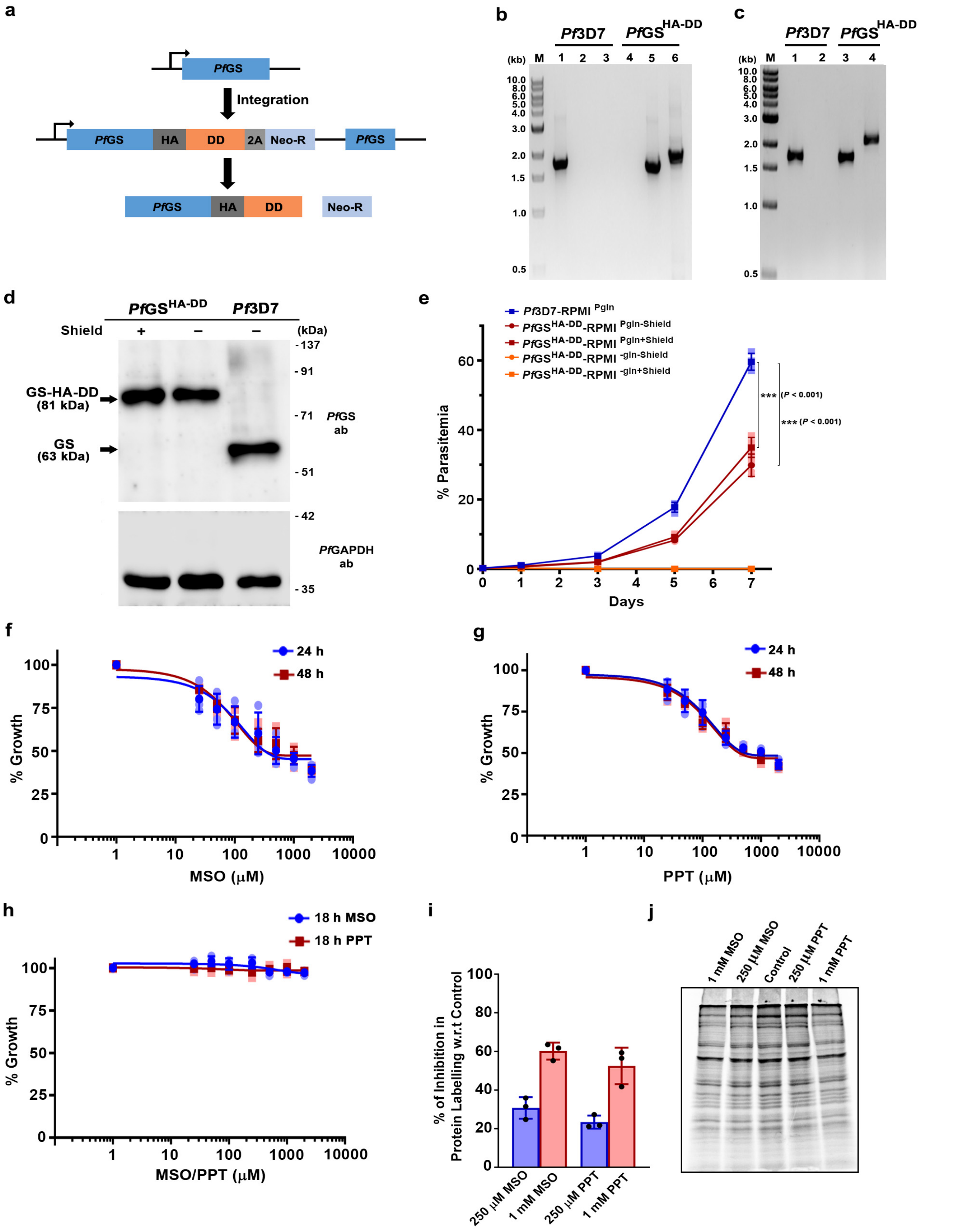

Supplementary Figure 5: Conditional knockdown of GS in *Pf*, differential inhibition of *Pf* and *Pb* parasites by MSO and PPT in RPMI^{Pgln} and *in vitro* metabolic labelling of *Pf* cultures in RPMI^{Pgln} medium. a, Schematic representation of the recombination approach followed to generate *Pf*GS$^{HA\text{-}DD}$ parasites. **b,** Genomic DNA PCR confirmation for *Pf*GS$^{HA\text{-}DD}$ parasites. Lane 1 and 4: 1.83 kb product amplified with GS-specific forward and 3' UTR-specific reverse primers to confirm the loci-specific integration. Lane 2 and 5: 1.86 kb product amplified with GS-specific forward and HA-specific reverse primers to confirm the in-frame

fusion. Lane 3 and 6: 2.2 kb product amplified with GS-specific forward and DD-specific reverse primers to confirm the in-frame fusion. Lane M: 1 kb ladder. **c,** RT-PCR confirmation for *Pf*GS$^{HA\text{-}DD}$ parasites. Lane 1 and 3: 1.63 kb product amplified with GS-specific forward and reverse primers**.** Lane 2 and 4: 2.07 kb product amplified with GS-specific forward and DD-specific reverse primers. Lane M: 1 kb ladder. **d,** Western blot detection of GS-HA-DD fusion in *Pf*GS$^{HA\text{-}DD}$ parasites. Upper panel: Detection of 81 kDa fusion protein in *Pf*GS$^{HA\text{-}DD}$ parasites with *Pf*GS antibody in the presence and absence of Shield-1. Lower panel: Parasite GAPDH as a loading control. For **b-d**, n = 3 independent experiments **e,** Asexual stage growth analysis of *Pf*3D7 parasites in RPMI^{Pgln}, and *Pf*GS$^{HA\text{-}DD}$ parasites in RPMI^{Pgln} and RPMI^{-gln} medium. (mean $\pm$ SD; ****P*<0.001, Two-way ANOVA). n = 5 independent experiments. **f,** Effect of MSO on *in vitro* cultures of *Pf* in RPMI^{Pgln}. n = 4 independent experiments. **g,** Effect of PPT on *in vitro* cultures of *Pf* in RPMI^{Pgln}. n = 3 independent experiments. **h,** Effect of MSO and PPT on *in vitro* single-cycle cultures of *Pb* maintained in RPMI^{Pgln}. n = 3 independent experiments. Growth assessment was carried out based on ^{3}H-hypoxanthine uptake and verified by Giemsa-stained smears. For **a-c**, the data (mean $\pm$ SD) represent percentage of growth with respect to the control. **i,** *In vitro* metabolic labelling of *Pf* cultures for 12 h with [^{35}S]-Methionine and -Cysteine in RPMI^{Pgln} medium. Percentage of inhibition (mean $\pm$ SD) observed for MSO and PPT with respect to solvent control is shown. n = 3 independent experiments. **j,** SDS-PAGE analysis of protein labelling for *Pf* proteins. Phosphorimager scan was performed after overnight exposure. n = 2 independent experiments. Source data and silver-stained gel representing phosphorimager scan are provided as a Source Data file.

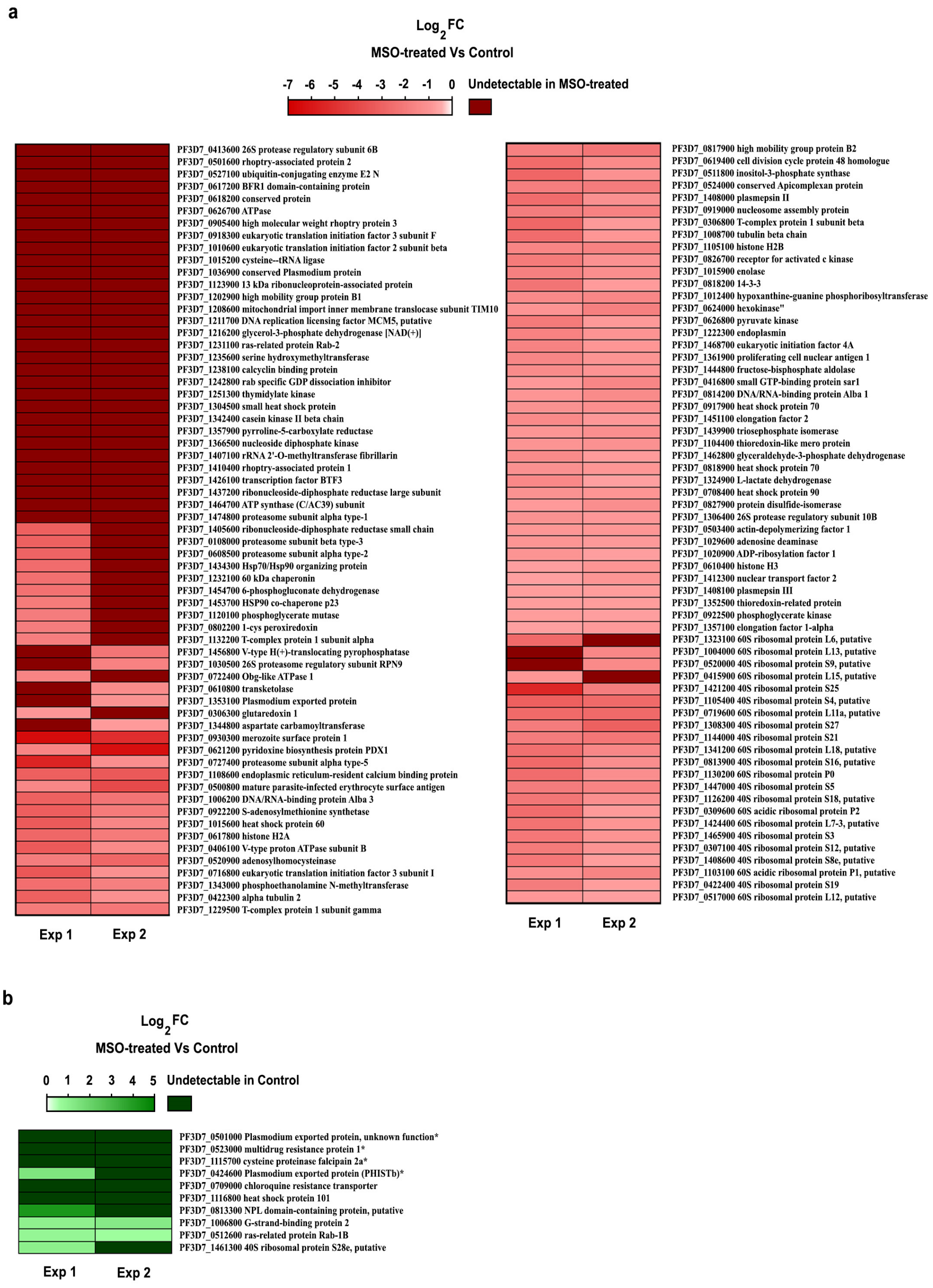

Supplementary Figure 6: Proteomic analysis of MSO-treated *Pf*3D7 parasites. a, List of downregulated proteins in MSO-treated *Pf*3D7 parasites. The list excludes asparagine-rich proteins that are provided in Fig. 9j. *Pf*3D7 cultures synchronized for late rings were treated with 50 µM MSO for 12 h. Proteins identified in both the untreated controls of two independent experiments and either undetectable or significantly downregulated (≥1.5 fold) in MSO-treated *Pf*3D7 parasites are represented. **b,** List of upregulated proteins in MSO-treated *Pf*3D7 parasites. Proteins significantly upregulated (≥1.5 fold) in both the MSO-treated parasites of

two independent experiments and/or undetectable in the untreated controls are represented. Asparagine-rich proteins are highlighted with asterisks. Proteins that contained $\geq$10% asparagine or at least one asparagine repeat containing 5 or more asparagine residues were considered as asparagine-rich.

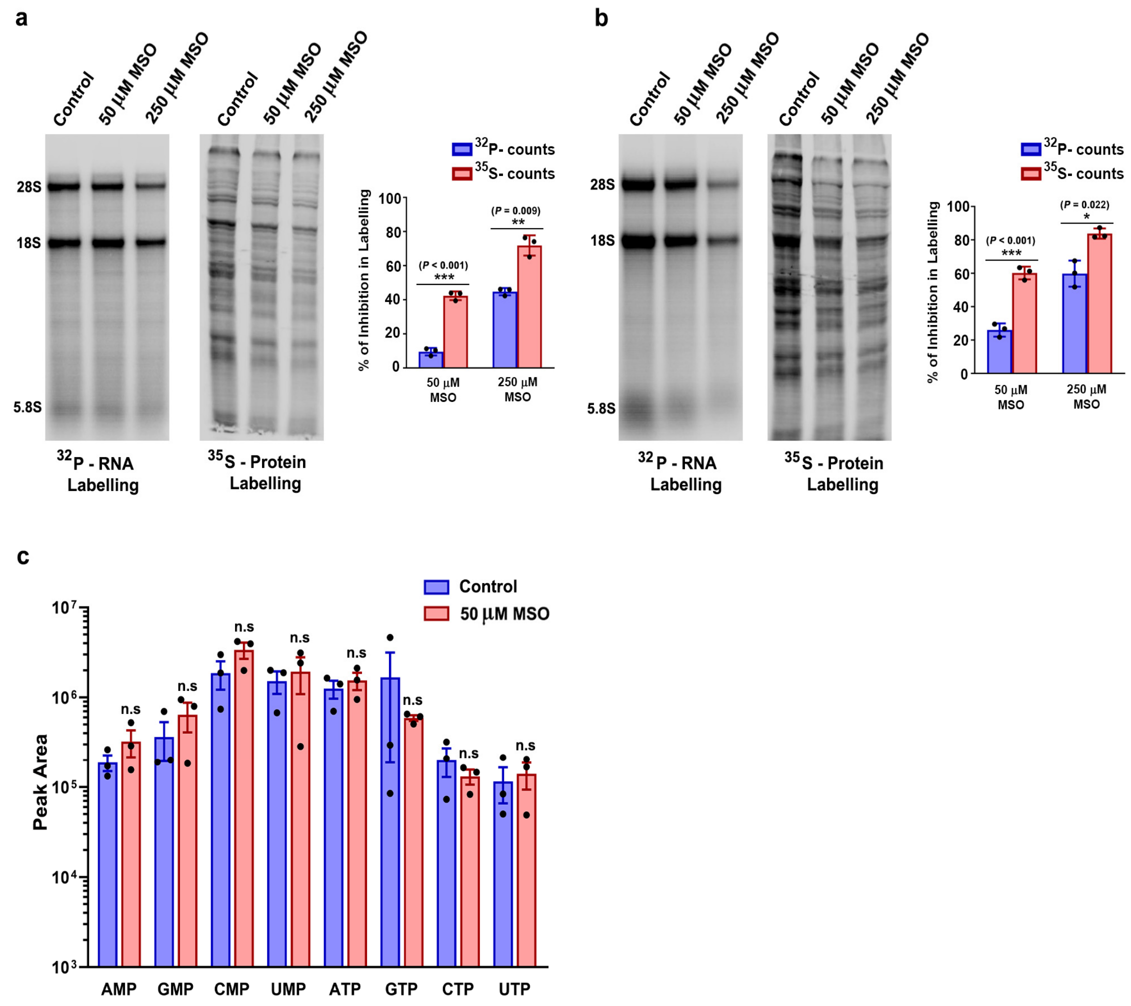

Supplementary Figure 7: Assessment of RNA synthesis and nucleotide levels in *Pf* cultures treated with MSO. a,b, Assessment of RNA synthesis by ^{32}P-orthophosphoric acid radiolabelling and protein synthesis by [^{35}S]-Methionine and -Cysteine radiolabelling for *Pf* ring (**a**) and trophozoite (**b**) stages, respectively. MSO treatment was performed for 12 h in RPMI^{-gln} medium. For **a** and **b**, phoshorimager scans of RNA transferred from 1.2% agarose gel to nylon membrane and proteins resolved in 10% SDS-PAGE are shown after overnight exposure. In addition, the percentage of inhibition (mean $\pm$ SD) for total ^{32}P and ^{35}S counts of radiolabelled RNA and proteins, respectively, in MSO-treated cultures with respect to solvent control are shown. (**P*<0.05, ***P*<0.01, ****P*<0.001, unpaired t-test; two-sided). n = 3 independent experiments. **c,** Assessment of nucleotide levels in *Pf* cultures treated with MSO. *Pf*3D7 cultures synchronized for late rings were treated with 50 µM MSO for 12 h. Nucleotides were extracted and mass spectrometry analyses were performed. Peak areas (mean $\pm$ SEM) obtained for the respective nucleotides are shown. (n.s - not significant, unpaired t-test; two-sided). n = 3 independent experiments. Source data and silver-stained gels representing phosphorimager scans are provided as a Source Data file.

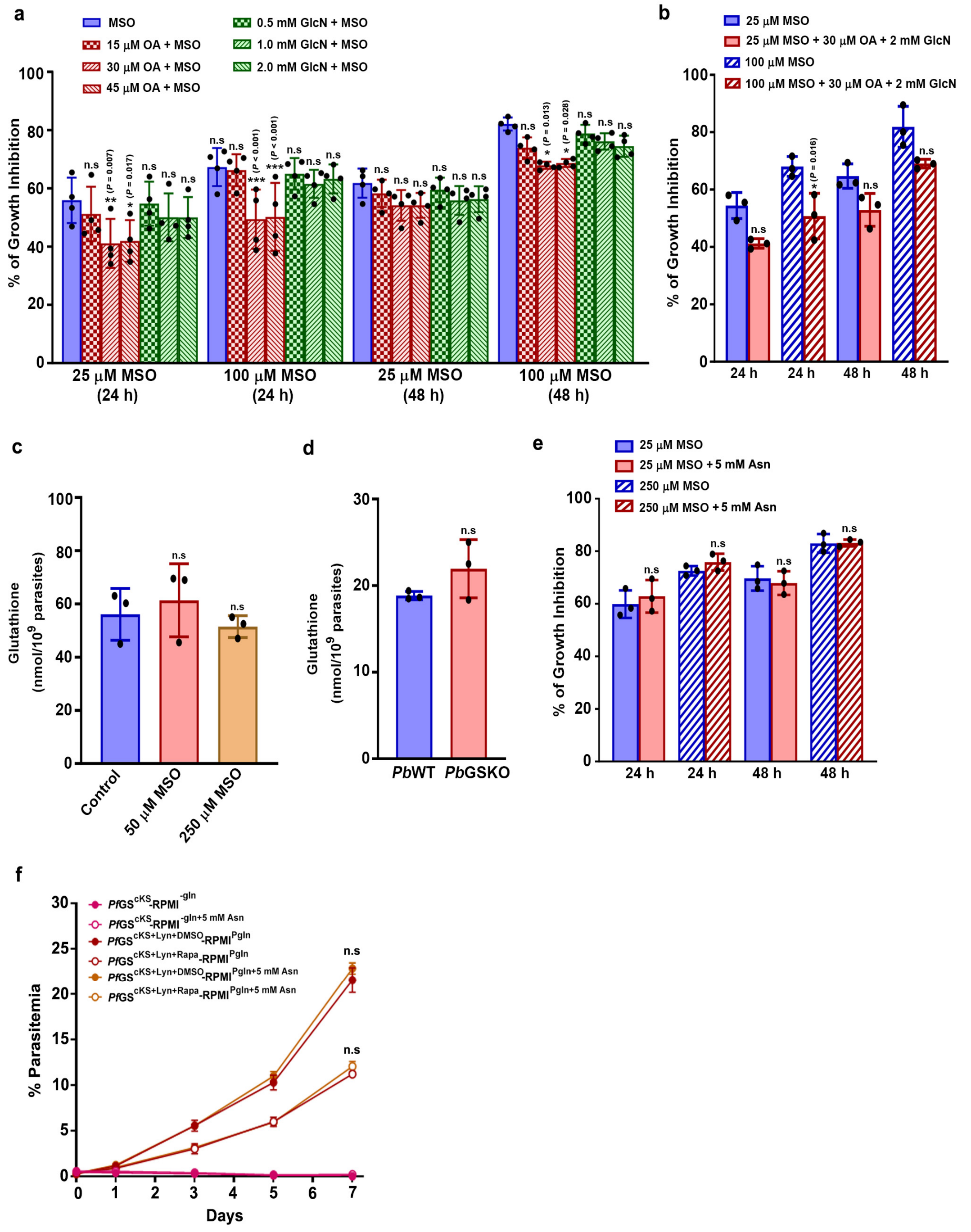

Supplementary Figure 8: Assessment of glutathione synthesis, and OA, GlcN and Asn supplementation. a, Percentage of *Pf* growth inhibition with respect to untreated control for 25 and 100 μM concentrations of MSO in the presence and absence of different concentrations of OA and GlcN. n = 4 independent experiments. (mean $\pm$ SD; **P*<0.05, ***P*<0.01, ****P*<0.001, Two-way ANOVA). **b,** Percentage of *Pf* growth inhibition with respect to untreated control for 25 and 100 μM concentrations of MSO supplemented with the combination of OA and GlcN. n = 3 independent experiments. (mean $\pm$ SD; **P*<0.05, Two-way ANOVA). Growth assessment in RPMI^{-gln} medium was carried out for 24 h and 48 h. The

supplementation experiments were carried out with less MSO concentrations since the purpose was to check the restoration of parasite growth. **c,** Total glutathione levels in MSO-treated *Pf* parasites. MSO treatment was carried out for 12 h in RPMI^{-gln} medium. **d,** Total glutathione levels in *Pb*WT and *Pb*GSKO parasites. Parasites predominantly in trophozoites were isolated from infected Balb/c mice. Parasitemia and the total number of infected RBCs were determined. n = 3. (mean $\pm$ SD; n.s - not significant, unpaired t-test). **e,** Percentage of *Pf* growth inhibition with respect to the untreated control for 25 and 250 μM MSO in the presence and absence of 5 mM asparagine in RPMI^{-gln} medium. n = 3 independent experiments. (mean $\pm$ SD; n.s - not significant, unpaired t-test). **f,** Effect of asparagine supplementation on the asexual growth of *Pf*GS^{cKS} parasites in RPMI^{-gln} medium and *Pf*GS$^{cKS+Lyn}$ parasites in RPMI^{Pgln} medium. (mean $\pm$ SD; n.s - not significant, Two-way ANOVA). Rapa - rapamycin. n = 3 independent experiments. OA - orotic acid; GlcN – glucosamine; Asn - asparagine. Source data are provided as a Source Data file.

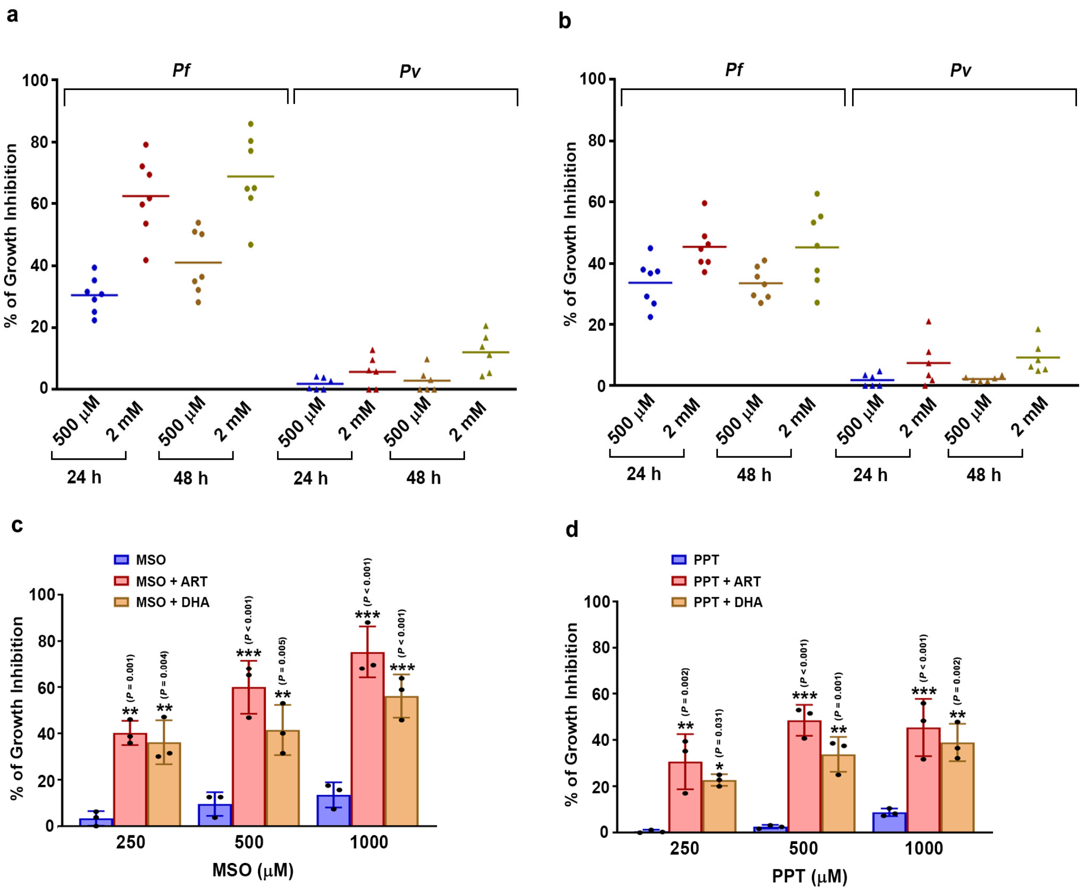

Supplementary Figure 9: Effect of MSO and PPT on *Pf* and *Pv* clinical isolates and ART-resistant *Pf*Cam3.I^{R539T} strain in RPMI^{Pgln}. a, Effect of MSO on *in vitro* growth of *Pf* (n=7) and *Pv* (n=6) clinical isolates in RPMI^{Pgln}. **b,** Effect of PPT on *in vitro* growth of *Pf* (n=7) and *Pv* (n=6) clinical isolates in RPMI^{Pgln}. Growth assessment was carried out based on ^{3}H-hypoxanthine uptake and verified by Giemsa-stained smears. **c,** Effect of ART/DHA and MSO combination on the growth of ART-resistant *Pf*Cam3.I^{R539T} parasites in RSA performed with RPMI^{Pgln} medium. Percentage of growth inhibition of ART/DHA and MSO combination treatment was determined at 96 h with respect to ART/DHA-treated parasites. Percentage of growth inhibition of MSO treatment alone was determined at 96 h with respect to untreated parasites. ART/DHA and MSO exposures were carried out for 6 h at ring stages. **d,** Effect of ART/DHA and PPT combination on the growth of ART-resistant *Pf*Cam3.I^{R539T} parasites in RSA performed with RPMI^{Pgln} medium. Percentage of growth inhibition of ART/DHA and PPT combination treatment was determined at 96 h with respect to ART/DHA-treated parasites. Percentage of growth inhibition of PPT treatment alone was determined at 96 h with respect to untreated parasites. ART/DHA and PPT exposures were carried out for 6 h at ring stages. Growth assessment was carried out based on ^{3}H-hypoxanthine uptake and verified by Giemsa-stained smears and flow cytometry. n = 3 independent experiments. (mean ± SD; *P<0.05, **P<0.01, ***P<0.001, Two-way ANOVA). Source data are provided as a Source Data file.

Supplementary References

1) Gill, H. S. & Eisenberg, D. The crystal structure of phosphinotricin in the active site of glutamine synthetase illuminates the mechanism of enzymatic inhibition. *Biochemistry* **40,** 1903-1912 (2001).

2) Krajewski, W. W., Jones, T. & Mowbray, S. L. Structure of *Mycobacterium tuberculosis* glutamine synthetase in complex with a transition-state mimic provides functional insights. *Proc. Natl. Acad. Sci. USA* **102,** 10499-10504 (2005).

3) Joo, H. K., Park, Y. W., Jang, Y. Y. & Lee, J. Y. Structural Analysis of Glutamine Synthetase from *Helicobacter pylori*. *Sci. Rep.* **8,** 11657 (2018).

4) van Rooyen, J. M., Abratt, V. R., Belrhali, H. & Sewell, T. Crystal structure of Type III glutamine synthetase: surprising reversal of the inter-ring interface. *Structure* **19,** 471-483 (2011).

5) Rydzak, T. et al. Deletion of Type I glutamine synthetase deregulates nitrogen metabolism and increases ethanol production in *Clostridium thermocellum*. *Metab. Eng.* **41,** 182-191 (2017).

6) Ho, C-M. et al. Bottom-up structural proteomics: cryoEM of protein complexes enriched from the cellular milieu. *Nat. Methods* **17,** 79-85 (2020).

7) Krajewski, W. W. et al. Crystal structures of mammalian glutamine synthetases illustrate substrate-induced conformational changes and provide opportunities for drug and herbicide design. *J. Mol. Biol.* **375,** 217-228 (2008).

8) Amaya, K. R., Kocherginskaya, S. A., Mackie, R.I. & Cann, I. K. O. Biochemical and Mutational Analysis of Glutamine Synthetase Type III from the Rumen Anaerobe *Ruminococcus albus* 8. *J. Bacteriol.* **187,** 7481–7491 (2005).

9) Prommana, P. et al. Inducible knockdown of *Plasmodium* gene expression using the glmS ribozyme. *PLoS One* **8,** e73783 (2013).

10) Miller, E.S. & Brenchley, J.E. L-Methionine SR-sulfoximine-resistant glutamine synthetase from mutants of *Salmonella typhimurium*. *J. Biol. Chem.* **256,** 11307-11312 (1981).

11) Harth, G., Clemens, D.L. & Horwitz, M.A. Glutamine synthetase of *Mycobacterium tuberculosis*: extracellular release and characterization of its enzymatic activity. *Proc. Natl. Acad. Sci. USA* **91(20),** 9342-9346 (1994).

12) Alibhai, M. & Villafranca, J.J. Kinetic and mutagenic studies of the role of the active site residues Asp-50 and Glu-327 of *Escherichia coli* glutamine synthetase. *Biochemistry* **33,** 682-686 (1994).

13) Ishiyama, K., Inoue, E., Yamaya, T. & Takahashi, H. Gln49 and Ser174 residues play critical roles in determining the catalytic efficiencies of plant glutamine synthetase. *Plant Cell Physiol.* **47,** 299-303 (2006).
14) Listrom, C.D. et al. Expression, purification, and characterization of recombinant human glutamine synthetase. *Biochem. J.* **328,** 159-163 (1997).
15) Dhalla, A.M. et al. Regeneration of catalytic activity of glutamine synthetase mutants by chemical activation: exploration of the role of arginines 339 and 359 in activity. *Protein Sci.* **3,** 476-481 (1994).
