# Supplementary material for: Distinct evolution of type I glutamine synthetase in Plasmodium and its species-specific requirement
Source: Nat Commun. 2023 Jul 14;14:4216. doi: 10.1038/s41467-023-39670-4 (PMC10349072; doi:10.1038/s41467-023-39670-4)
Supplement: Supplementary file 2 — Description of Additional Supplementary Files [file 41467_2023_39670_MOESM2_ESM.pdf]

Description of Additional Supplementary Files

Supplementary Data 1: Analyses of hydrogen bonds in *Pf*GS and ΔI$_2$*Pf*GS. Hydrogen bonds unique for *Pf*GS and ΔI$_2$*Pf*GS are listed for all the four subunits used in MD simulations. Hydrogen bonds having $\geq$40% occupancy are considered. Hydrogen bonds of the key residues associated with enzyme activity from the adjacent subunits (subunit A and B) are represented to highlight the variations between *Pf*GS and ΔI$_2$*Pf*GS. Hydrogen bonds that were either absent or with significantly reduced occupancies (<40%) are highlighted in blue. The entire list of hydrogen bonds are also provided for *Pf*GS and ΔI$_2$*Pf*GS.

Supplementary Data 2: Cumulative peptide intensities and normalized FC and Log$_2$FC values of downregulated and upregulated proteins in MSO-treated *Pf*3D7 parasites. The cumulative peptide intensities of the proteins that were commonly downregulated and upregulated in two independent sets of experiments are shown. For cumulative peptide intensities, peptides identified with $\geq$95% confidence for the respective proteins were considered. Fold change (FC) values normalized with respect to parasite actin I are given along with Log$_2$FC values. For downregulated proteins, proteins identified in both the untreated controls of two independent experiments and either undetectable or significantly downregulated ($\geq$1.5 fold) in MSO-treated *Pf*3D7 parasites are represented. For upregulated proteins, proteins significantly upregulated ($\geq$1.5 fold) in both the MSO-treated parasites of two independent experiments and/or undetectable in the untreated controls but detectable in the MSO-treated parasites are represented. Asparagine-rich proteins are highlighted in bold. The entire peptide summary for the respective controls and MSO-treated samples are also provided.
