# Supplementary material for: Distinct evolution of type I glutamine synthetase in Plasmodium and its species-specific requirement
Source: Nat Commun. 2023 Jul 14;14:4216. doi: 10.1038/s41467-023-39670-4 (PMC10349072; doi:10.1038/s41467-023-39670-4)
Supplement: Supplementary file 5 — Reporting Summary [file 41467_2023_39670_MOESM5_ESM.pdf]

nature portfolio

Corresponding author(s): Viswanathan Arun Nagaraj

Last updated by author(s): Jun 1, 2023

Reporting Summary

Nature Portfolio wishes to improve the reproducibility of the work that we publish. This form provides structure for consistency and transparency in reporting. For further information on Nature Portfolio policies, see our Editorial Policies and the Editorial Policy Checklist.

Statistics

For all statistical analyses, confirm that the following items are present in the figure legend, table legend, main text, or Methods section.

n/a	Confirmed	
☐	☒	The exact sample size (*n*) for each experimental group/condition, given as a discrete number and unit of measurement
☐	☒	A statement on whether measurements were taken from distinct samples or whether the same sample was measured repeatedly
☐	☒	The statistical test(s) used AND whether they are one- or two-sided<br>*Only common tests should be described solely by name; describe more complex techniques in the Methods section.*
☐	☒	A description of all covariates tested
☐	☒	A description of any assumptions or corrections, such as tests of normality and adjustment for multiple comparisons
☐	☒	A full description of the statistical parameters including central tendency (e.g. means) or other basic estimates (e.g. regression coefficient) AND variation (e.g. standard deviation) or associated estimates of uncertainty (e.g. confidence intervals)
☐	☒	For null hypothesis testing, the test statistic (e.g. *F*, *t*, *r*) with confidence intervals, effect sizes, degrees of freedom and *P* value noted<br>*Give P values as exact values whenever suitable.*
☒	☐	For Bayesian analysis, information on the choice of priors and Markov chain Monte Carlo settings
☒	☐	For hierarchical and complex designs, identification of the appropriate level for tests and full reporting of outcomes
☒	☐	Estimates of effect sizes (e.g. Cohen's *d*, Pearson's *r*), indicating how they were calculated

Our web collection on statistics for biologists contains articles on many of the points above.

Software and code

Policy information about availability of computer code

Data collection	No software was used.
Data analysis	GraphPad Prism Version 7.00 software was used to plot the graphs. Statistical analyses were carried out using unpaired Welch's t-test (two-sided), two-way ANOVA and log-rank (Mantel-Cox) test. Tukey test was perfomed for two-way ANOVA and multiple comparisons were corrected by statistical hypothesis testing. n.s - not significant, *P<0.05, **P<0.01, ***P<0.001. The non-linear regression fit for inhibitor versus response curve was performed and R-squared values were calculated using GraphPad Prism 7.00. Homology modeling was performed using MODELLER v10.1. and PyMOL Molecular Graphics System, Version 1.2r3pre, Schrödinger, LLC. For MD simulations, preliminary topologies and coordinates for the protein complexes were generated in VMD 1.9.3 using CHARMM34 force field. MD simulations were done using NAMD 2.9. Particle Mesh Ewald (PME) sum algorithm was used to calculate the long-range electrostatic interactions with fixed periodic boundary conditions. The covalent interactions involving hydrogens were constrained using SHAKE algorithm. Version numbers are not applicable for PME sum and SHAKE algorithms. Analyst TF1.7.1. software was used for proteomics mass spectrometry data acquisition. Paragon algorithm (ProteinPilot Software Version 5.0.2, SCIEX) was used to annotate MS/MS data. Processing, visualization and analysis of mass spectrometry data for nucleotides were carried out using MZmine Version 2.53.

For manuscripts utilizing custom algorithms or software that are central to the research but not yet described in published literature, software must be made available to editors and reviewers. We strongly encourage code deposition in a community repository (e.g. GitHub). See the Nature Portfolio guidelines for submitting code & software for further information.

Data

Policy information about availability of data

All manuscripts must include a data availability statement. This statement should provide the following information, where applicable:

- Accession codes, unique identifiers, or web links for publicly available datasets
- A description of any restrictions on data availability
- For clinical datasets or third party data, please ensure that the statement adheres to our policy

Source data are provided with this paper. Reference proteomes of Plasmodium falciparum (UP000001450, Taxonomy: 36329 https://www.uniprot.org/uniprotkb?query=UP000001450; UP000030688, Taxonomy: 57266 https://www.uniprot.org/uniprotkb?query=UP000030688; UP000054282, Taxanomy: 57267 https://www.uniprot.org/uniprotkb?query=UP000054282; UP000054289, Taxonomy: 137071 https://www.uniprot.org/uniprotkb?query=UP000054289; UP000030673, Taxonomy: 5843 https://www.uniprot.org/uniprotkb?query=UP000030673; UP000019103, Taxonomy: 57270 https://www.uniprot.org/uniprotkb?query=UP000019103; UP000232684, Taxonomy: 5843 https://www.uniprot.org/uniprotkb?query=UP000232684) available at Uniprot (https://www.uniprot.org/) were used for proteomics analyses. Proteomics data have been deposited to the ProteomeXchange Consortium via the PRIDE partner repository with the dataset identifier PXD032797 https://www.ebi.ac.uk/pride/archive/projects/PXD032797. The structures of PfGS (PDB ID: 6PEW; https://www.rcsb.org/structure/6PEW), St (PDB ID: 1F1H; https://www.rcsb.org/structure/1F1H), Mt (PDB ID: 2WGS; https://www.rcsb.org/structure/2WGS) and Hp GS (PDB ID: 5ZLP; https://www.rcsb.org/structure/5ZLP) were accessed from RCSB Protein Data Bank (https://www.rcsb.org/).

Human research participants

Policy information about studies involving human research participants and Sex and Gender in Research.

Reporting on sex and gender	Sex- and gender-based analyses are not applicable since the study involves in vitro experiments with clinical isolates and does not include any data from human research participants.
Population characteristics	This work does not include any data from human research participants. We have only used the clinical isolates of Pf and Pv that were collected from malaria-infected patients for performing in vitro experiments. Covariate-relevant population characteristics do not influence the results of this study since the clinical isolates were used only for in vitro culture treatment studies. The age group was between 10-60 years (Male: 19, 19, 24, 26, 45, 48, 50 and 52 and Female: 20, 25, 27, 35 and 51).
Recruitment	Blood samples were collected from febrile patients aged between 10-60 years (male and female) who visited Ispat General Hospital, Rourkela, India, and KMC Hospital, Mangalore, India. Pf and Pv infections were confirmed by examining Giemsa-stained thick and thin blood smears under light microscope, and by performing rapid diagnostic test and PCR for 18S rRNA. The infected blood was collected in heparinized vacutainers and the subsequent procedures were carried out in BSL-2 facility under sterile conditions. Patients were recruited based on the confirmation for infections by light microscopy, rapid diagnostic test and PCR. We have excluded children below 10 years of age and adults above 60 years, mixed infection with another Plasmodium species, patients with human immunodeficiency virus (HIV) infection or other immunosuppressive disorders, hepatitis, evidence of clinically significant cardiovascular, pulmonary, metabolic, gastrointestinal, neurological, endocrine diseases, malignant disorders and pregnancy. There were no self-selection bias or other biases that impact this recruitment.
Ethics oversight	The collection of clinical samples was carried out with the approval of Institutional Ethics Committee (IEB/IEC)/ Institutional Review Board (IRB) (94/HEC/19, Institute of Life Sciences, Bhubaneswar; IEC:248/2019, KMC Hospital, Mangalore). Informed consent was taken from the participants.

Note that full information on the approval of the study protocol must also be provided in the manuscript.

Field-specific reporting

Please select the one below that is the best fit for your research. If you are not sure, read the appropriate sections before making your selection.

☒ Life sciences ☐ Behavioural & social sciences ☐ Ecological, evolutionary & environmental sciences

For a reference copy of the document with all sections, see nature.com/documents/nr-reporting-summary-flat.pdf

Life sciences study design

All studies must disclose on these points even when the disclosure is negative.

Sample size	There is a vast amount of literature available and three independent protein preparations or parasite preparations are adequate to ensure the statistical significance and reproducibility. The experiments involving glutamine synthetase (GS) enzyme assays, kinetic characterization, feedback regulation, adenylylation etc were carried out at least with three indpendent preparations of recombinant proteins. The endogenous GS activity in P. falciparum (Pf) and P. berghei (Pb) lysates was confirmed with at least three different parasite preparations. The results obtained for adenylylation with recombinant parasite GS were verified with endogenous GS present in the Pf and Pb lysates. Recombinant E. coli GS was also used as a control wherever it was appropriate. Immunofluorescence studies confirming GS expression and

localization in the asexual stages, sexual stages and exo-erythrocytic stages of P. falciparum and P. berghei were performed with at least two independent batches to ensure the reproducibility. In addition, the specificity of the antibodies raised against parasite GS was confirmed by the absence of protein and by the absence of fluorescence signal in the GS knockout parasites. The results pertaining to the activities of parasite GS lacking peptide inserts were verified with at least three different independent preparations. The conditional gene-targeted parasites generated for PfGS were verified by genomic DNA PCR, RT-PCR, Western, live fluorescence and mislocalization analyses. The data provided for PfGS cKO phenotype in the asexual and gametocyte stages represent three different batches. All rapamycin treatment cKO experiments were performed with the background strain as a control. The phenotype of PbGS deletion was verified with two independent clones. The data provided for HA-DD PfGS parasites represent at least three independent experiments. The animal experiments in mice were also carried out with at least two independent batches. The number of mice were chosen taking into consideration of the mortality that occur due to cerebral malaria and anemia, and based on the available literature. A vast literature is available for malaria experiments in mice. We also performed power analysis using G*Power 3.1.9.7 with the mean and standard deviation values obtained from pilot studies for the respective parameters to determine the number of mice. The effect sizes were determined and an alpha value of 0.05 and a power of 0.80 were used to determine the group size. The data for mosquito experiments represent three independent batches. The number of mice and mosquitoes used were provided in the figure legends. Inhibition studies carried out with methionine sulfoximine (MSO) and phosphinothricin (PPT), metabolic labelling, amino acid quantification and nucleotide analyses represent at least three different experiments. Proteomics data represent two independent experiments. To provide stringent criteria for downregulated proteins, we considered only those proteins that were detected in both the controls and were either undetectable or 1.5 fold less in MSO-treated samples. Peptides with or above 95% confidence were included for analysis. Normalization was also performed with respect to parasite actin. Nucleotide analyses were performed with three independent preparations. Pf and Pv clinical sample studies were performed with at least six different patient samples to verify MSO and PPT inhibition. Artemisinin sensitivity experiments in Pf Cambodia strain were performed in three independent batches. The sample size for the data are provided in the respective figure legends. These approaches were sufficient to perform statistical comparison, and ensure statistical significance and reproducibility of the observations made.

Data exclusions

No data were excluded from the analyses.

Replication

The reproducibility of the experiments was verified by (i) performing the experiments in multiple independent batches (ii) examining the results with independent mice and (iii) confirming the findings independently by at least two different authors. The exact details of the number of mice, experiments etc are provided in the respective figure legends. The reproducibility of recombinant protein based experiments and parasite lysates were confirmed with independent preparations. All attempts at replication were successful.

Randomization

Mice, mosquitoes and parasite cultures were allocated randomly for the experiments. Randomization does not apply for the experiments performed with recombinant proteins.

Blinding

The experiments performed were carried out with recombinant proteins, cultures and by infecting mice with wildtype and knockout parasites. Therefore, blinding was not possible. However, the experiments were verified by at least two independent authors or wherever it was appropriate, the experiments were initiated by one author and the results were collected by another author who did not know about the groups. The authenticity of the knockout/conditional gene-targeted parasites were routinely verified by PCR and Western analyses to ensure that the results obtained were for the knockout/conditional gene-targeted parasites. These confirmations were carried out by author who was not aware of the groups and results. For experiments involving recombinant protein enzyme assays, blinding is not possible since the target assays specific for GS have to be carried out.

Reporting for specific materials, systems and methods

We require information from authors about some types of materials, experimental systems and methods used in many studies. Here, indicate whether each material, system or method listed is relevant to your study. If you are not sure if a list item applies to your research, read the appropriate section before selecting a response.

Materials & experimental systems

n/a	Involved in the study
☐	☒ Antibodies
☐	☒ Eukaryotic cell lines
☒	☐ Palaeontology and archaeology
☐	☒ Animals and other organisms
☒	☐ Clinical data
☒	☐ Dual use research of concern

Methods

n/a	Involved in the study
☒	☐ ChIP-seq
☒	☐ Flow cytometry
☒	☐ MRI-based neuroimaging

Antibodies

Antibodies used

Primary Antibodies
Mouse monoclonal His-tag antibody (1:4000 dilution, Sigma-Aldrich, H1029); https://www.sigmaaldrich.com/deepweb/assets/sigmaaldrich/product/documents/297/978/h1029dat.pdf
Rabbit polyclonal anti-GFP antibodies (1:5000 dilution, Abcam, ab290); https://www.abcam.com/gfp-antibody-ab290.html
Anti-UIS4 antibody (1:1000 dilution, Origene, AB0042-200); https://www.origene.com/catalog/antibodies/primary-antibodies/ab0042-200/uis4-goat-polyclonal-antibody
Phosphorylated eIF2α (1:1000 dilution, Cell Signaling Technology, #3398); https://www.cellsignal.com/products/primary-antibodies/phospho-eif2a-ser51-d9g8-xp-rabbit-mab/3398
eIF2α (1:1000 dilution, Cell Signaling Technology, #9722); https://www.cellsignal.com/products/primary-antibodies/eif2a-antibody/9722

Secondary Antibodies
Goat Anti-Mouse IgG H&L (HRP) (1:10000 dilution, Abcam, ab97023); https://www.abcam.com/goat-mouse-igg-hl-hrp-ab97023.html
Goat Anti-Rabbit IgG H&L (HRP) (1:10000 dilution, Abcam, ab97051); https://www.abcam.com/goat-rabbit-igg-hl-hrp-ab97051.html
Goat Anti-Mouse IgG H&L (AP) (1:5000 dilution, Abcam, ab97020); https://www.abcam.com/goat-mouse-igg-hl-alkaline-phosphatase-ab97020.html
FITC-conjugated donkey anti-mouse IgG (1:250 dilution, Thermo Fisher Scientific, A24501); https://www.thermofisher.com/antibody/product/Donkey-anti-Mouse-IgG-H-L-Secondary-Antibody-Polyclonal/A24501
Rabbit anti-goat AF594 (1:400 dilution, Thermo Fisher Scientific, A-11080); https://www.thermofisher.com/antibody/product/Rabbit-anti-Goat-IgG-H-L-Cross-Adsorbed-Secondary-Antibody-Polyclonal/A-11080

Anti-AMP-tyrosine antibody (clone 1G11) was provided by Prof. Aymelt Itzen, Center for Experimental Medicine, Biochemistry and Signal Transduction, UKE, Hamburg, Germany. Polyclonal sera against recombinant parasite GS and parasite GAPDH were raised in the present study.

Validation

For commercial antibodies, validation details are available in the manufacturers' page. The relevant webpage links have been provided above. For Anti-AMP-tyrosine antibody, the relevant reference is provided in the manuscript and the validation details are available at https://doi.org/10.1016/j.isci.2020.101800. Polyclonal sera validation results for the parasite GS and GAPDH are provided in the manuscript itself.

Eukaryotic cell lines

Policy information about cell lines and Sex and Gender in Research

Cell line source(s)

Pb ANKA (MRA-311); PfCam clinical isolate (IPC 5202) (MRA-1240); Pf3D7 strain (MRA-1001); HC-04 cell line (MRA-975). All of them were provided by Malaria Research and Reference Reagent Resource Center (MR4), ATCC, Manassas, Virginia (https://www.beiresources.org/Home.aspx).

Authentication

1) Pb ANKA (MRA-311) - Authenticated strain from Malaria Research and Reference Reagent Resource Center (MR4), ATCC, Manassas, Virginia. We further authenticated it by sequencing at least 10 genes from this strain including GS, GAPDH, ALAS, FC, 18srRNA. In addition, cerebral malaria phenotype was confirmed.
2) PfCam clinical isolate (IPC 5202) (MRA-1240) - Authenticated strain from Malaria Research and Reference Reagent Resource Center (MR4), ATCC, Manassas, Virginia. We further authenticated it by verifying artemisinin resistance.
3) Pf3D7 strain (MRA-1001) - Authenticated strain from Malaria Research and Reference Reagent Resource Center (MR4), ATCC, Manassas, Virginia. We further authenticated it by sequencing at least 10 genes from this strain including GS, GAPDH, ALAS, FC, 18srRNA. In addition, choloroquine and artemisinin sensitivities were confirmed.
4) HC-04 cell line (MRA-975) - Authenticated strain from Malaria Research and Reference Reagent Resource Center (MR4), ATCC, Manassas, Virginia. We authenticated it by examining its morphology.

Mycoplasma contamination

Negative

Commonly misidentified lines
(See ICLAC register)

No commonly misidentified lines were used.

Animals and other research organisms

Policy information about studies involving animals; ARRIVE guidelines recommended for reporting animal research, and Sex and Gender in Research

Laboratory animals

Balb/c male and female mice: 7-8 weeks old; C57BL/6 male and female mice: 7-8 weeks old. Breeding and maintenance of mice were carried out at the animal house facility of Institute of Life Sciences, Bhubaneswar, under standard conditions of 25 +/- 3ºC temperature, 40-50% relative humidity, and 12 h light / 12 h dark cycle. 5-7 days old Anopheles stephensi mosquitoes were used to perform the infection studies. The following organisms were procured from Malaria Research and Reference Reagent Resource Center (MR4), ATCC Manassas Virginia - Pb ANKA (MRA-311) deposited by Thomas F. McCutchan, PfCam clinical isolate (IPC 5202; MRA-1240) deposited by Didier Menard, Pf3D7 strain (MRA-1001) deposited by Megan G. Dowler, and A. stephensi vector (MRA-128) deposited by Mark Q. Benedict.

Wild animals

No wild animals were used in the study.

Reporting on sex

The findings do not apply to one sex. Both male and female mice were used in this study. For Figure 6g, 13 mice (7 male and 6 female) were used for both PbWT and PbGSKO. For Figure 6h, 6j and 6k, 13 mice (7 male and 6 female) were used for PbWT and 12 mice were used for PbGSKO (6 male and 6 female) were used for PbGSKO. For Figure 6i, 5 mice (3 male and 2 female) were used for both PbWT and PbGSKO. For Figure 6l, 6m, 6n and 6o, 3 mice (male) were used for both PbWT and PbGSKO. For Figure 7a, 6 mice (3 male and 3 female) were used for both PbWT and PbGSKO. For Figure 7b, 7 mice (3 male and 4 female) were used for both PbWT and PbGSKO. For Figure 7c and 7j, 3 mice (male) were used for both PbWT and PbGSKO. For Figure 8f and Supplementary figure 8d, 3 mice (male) were used for both PbWT and PbGSKO.

Field-collected samples

No field collected samples were used in the study.

Ethics oversight

The studies involving mice were approved by Institutional Animal Ethics Committee (ILS/IAEC-69-AH/AUG-16) and the experiments were carried out according to the national guidelines framed by "The Committee for the Purpose of Control and Supervision of Experiments on Animals (CPCSEA)".

Note that full information on the approval of the study protocol must also be provided in the manuscript.
